# Fanconi anemia associated protein 20 (FAAP20) plays an essential role in homology-directed repair of DNA double-strand breaks

**DOI:** 10.1038/s42003-023-05252-9

**Published:** 2023-08-24

**Authors:** Anna Palovcak, Fenghua Yuan, Ramiro Verdun, Liang Luo, Yanbin Zhang

**Affiliations:** 1 Department of Biochemistry & Molecular Biology, University of Miami Miller School of Medicine, Miami, FL 33136 USA; 2 Department of Medicine, University of Miami Miller School of Medicine, Miami, FL 33136 USA; 3 Sylvester Comprehensive Cancer Center, University of Miami Miller School of Medicine, Miami, FL 33136 USA

**Keywords:** Double-strand DNA breaks, DNA

## Abstract

FAAP20 is a Fanconi anemia (FA) protein that associates with the FA core complex to promote FANCD2/FANCI monoubiquitination and activate the damage response to interstrand crosslink damage. Here, we report that FAAP20 has a marked role in homologous recombination at a DNA double-strand break not associated with an ICL and separable from its binding partner FANCA. While FAAP20’s role in homologous recombination is not dependent on FANCA, we found that FAAP20 stimulates FANCA’s biochemical activity in vitro and participates in the single-strand annealing pathway of double-strand break repair in a FANCA-dependent manner. This indicates that FAAP20 has roles in several homology-directed repair pathways. Like other homology-directed repair factors, FAAP20 loss causes a reduction in nuclear RAD51 Irradiation-induced foci; and sensitizes cancer cells to ionizing radiation and PARP inhibition. In summary, FAAP20 participates in DNA double strand break repair by supporting homologous recombination in a non-redundant manner to FANCA, and single-strand annealing repair via FANCA-mediated strand annealing activity.

## Abstract (web summary)

FAAP20 has roles in several homology-directed pathways of DNA double-strand break repair that are both independent and dependent on its binding partner FANCA and FANCA-mediated single strand annealing.

## Introduction

Homology-directed repair (HDR) of genomic DNA is required for cancer cell proliferation and resistance to clastogenic therapies used for treatment^1–6^. Homologous recombination (HR) is the major HDR pathway that uses high-fidelity templated repair to preserve genomic stability at DNA double-strand breaks and strained replication forks^7–10^. Based on these characteristics, a tumor’s capacity to complete HR repair can guide therapy-response and provide unique, targetable vulnerabilities. This is especially seen in the cases of BRCA1/2-deficient cancers that are highly sensitive to blockage of “backup” HDR repair mechanisms such as the mutagenic single-strand annealing (SSA) and microhomology-mediated end-joining (MMEJ) pathways. This has spurred the development of small molecule inhibitors specific for the protein factors that mediate HDR pathways, such as PARP1 and RAD52^11–18^. While this strategy has had some success, the shortage of identifiable cancer patients susceptible to these treatments, coupled with the robustness of the DNA damage response that generates resistance mechanisms, has served as obstacles to these seemingly promising approaches. Accordingly, there is a need to expand the characterization of cancer gene expression profiles that lend well to exploitation of known synthetic lethality relationships, while simultaneously delineating potential avenues of treatment resistance.

FAAP20, or Fanconi anemia-associated protein 20, is a 20 kDa protein that is a member of the Fanconi anemia (FA) pathway of DNA repair. FAAP20 is a particularly elusive member of the FA pathway compared with other FA proteins due to a lack of structural data and assigned repair roles. However, FAAP20 has an established prognostic value associated with its protein expression in multiple cancer types, which warrants a closer look at its roles in genome maintenance and stability. According to The Human Protein Atlas, FAAP20 has differential prognostic status, depending on the cancer tissue type of origin, where its overexpression is unfavorable in liver cancer but favorable in pancreatic cancer^19^. FAAP20’s ability to influence cancer outcome in a cell-type-dependent manner indicates an unrealized and potentially powerful cellular role of FAAP20.

Although there is no structural data for FAAP20, it is known that FAAP20 contains a conserved C-terminal ubiquitin-binding zinc finger (UBZ) domain that binds polyubiquitin chains^20,21^. FAAP20 is then recruited to damaged chromatin via RNF8-mediated ubiquitination^21^ One established role of FAAP20 recruitment to damage sites is further FA core complex recruitment that leads to FA pathway activation at interstrand crosslink (ICL) damage. Within the FA core complex, FAAP20 can both promote ubiquitination of the FANCD2/FANCI heterodimer and translesion synthesis (TLS) through a direct interaction with ubiquitinated REV1 polymerase^22,23^. FAAP20 also directly interacts with FA core complex member FANCA^20,24^. It has been previously proposed that FAAP20 forms a subcomplex within the FA core complex known as “AG20” where it binds FANCA which also simultaneously binds FANCG. However, there is no structural evidence for the AG20 complex, and the role of AG20 in promoting ID2 ubiquitination has not yet been determined^25^. In addition to FANCD2/FANCI ubiquitination, FA core complex proteins appear in downstream repair processes through largely uncharacterized mechanisms. FA core complex proteins like FANCA have emerged as major mediators of ICL-coupled HR events^26^. In canonical HR that is not coupled to ICLs FA proteins such as FANCA, FANCC and FANCD2 demonstrate a minor role^27–30^, yet it is still not clear how and when FA proteins are able to support HR repair.

Although the majority of FAAP20 research thus far is related to its roles within the core complex and in ICL repair, there are indications that FAAP20 may participate in DNA double-strand break (DSB) repair that is separable from ICLs. RNF8-mediated ubiquitination, which is necessary for FAAP20 chromatin recruitment, is known as a major mechanism for recruitment and assembly of protein repair complexes at DSBs^31–35^. Also, the interaction of FAAP20 with REV1 suggests a role for FAAP20 in DSB repair because REV1 has been previously implicated in HR repair^36^. Lastly, the interaction between FAAP20 and FANCA may facilitate involvement of FAAP20 in FANCA-associated roles of DSB repair which include the single-strand annealing (SSA) pathway^27,30^. It has also been shown that FAAP20 is necessary for FANCA recruitment to site-specific laser-induced DSBs in cells^21^, but no follow-up studies so far have further elucidated this occurrence. Based on these previous findings and the limited knowledge of FAAP20 functions, we aimed to determine if FAAP20 had a role in DNA DSB repair that was not linked to ICL damage.

We found that FAAP20 has a surprisingly large role in all HDR pathways of DSB repair that we tested, and its roles in SSA likely involve FANCA whereas its roles in HR are not redundant with FANCA. However, FAAP20’s observed biochemical and cellular roles are not enhanced by FANCG, suggesting that FAAP20 does not need to form the AG20 subcomplex in order to participate in HDR. We also see that FAAP20 supports cellular colony formation and proliferation, which corresponds with its strong HR function and may underlie the ability of FAAP20 to affect cancer outcomes and therapy resistance.

## Results

### FAAP20 is required for dsDNA and ssDNA/RNA-templated DSB repair

To study the contribution of FAAP20 to HR repair we utilized the established DR-GFP reporter assay in U2OS cells using the 282-U2OS cell line^37^. In these cells, GFP expression occurs when an I-SceI-induced DSB is repaired through HR, which is then measured using flow cytometry (fluorescence-activated cell sorting (FACS)). One pervasive limitation of this assay has been the variation in I-SceI-plasmid transfection efficiency, which directly affects the levels of DSB-induction in between experimental groups and from experiment to experiment. To overcome this limitation, we inserted an active mCherry expression cassette under a constitutive CMV promoter within the same expression plasmid as the I-SceI nuclease (Supplementary Fig. 1a). We then report GFP+ cells as a % of the mCherry+ cell population, allowing for exclusion of non-transfected cells during analysis. This mCherry/I-SceI reporter plasmid is used for all reporter assays requiring I-SceI expression within this manuscript.

**Fig. 1:**
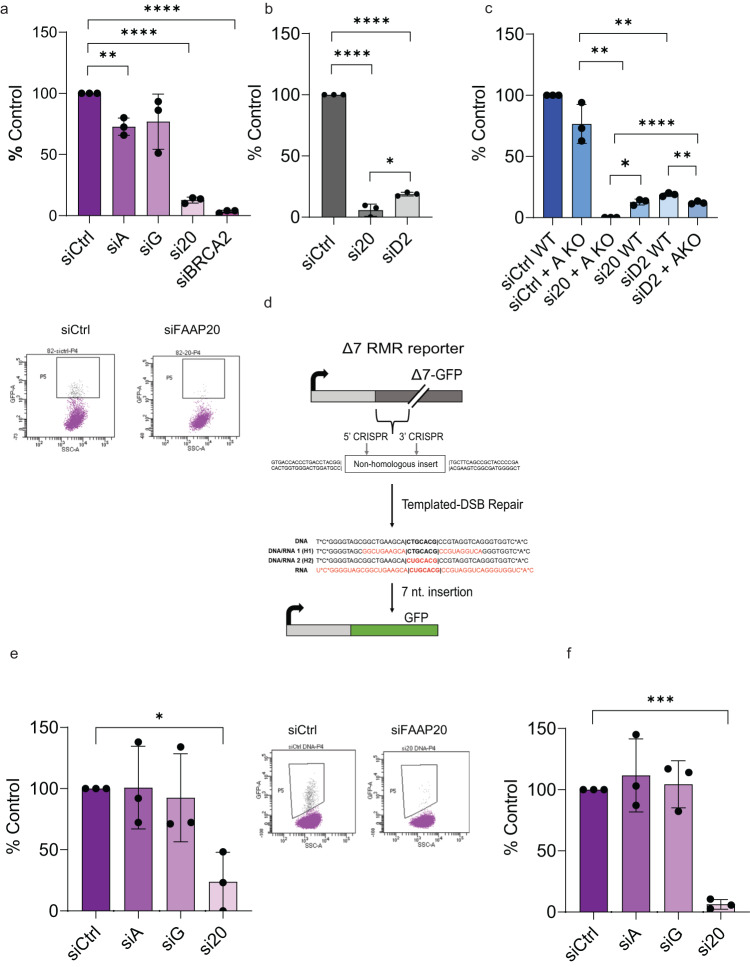
FAAP20 supports HR repair of DSBs through dsDNA and ssDNA/RNA-templated repair. **a** Measure of HR repair events at an I-SceI DSB in 282-U2OS reporter cell lines with the indicated knockdown conditions performed prior to DSB induction. Events are reported as % of siCtrl. BRCA2 knockdown is shown as a “HR factor control.” Representative dot plots are shown for effect. **b** Measure of HR events in 282-U2OS cells with FANCD2 siRNA knockdown compared with FAAP20 siRNA knockdown **c** Measure of HR events in 282-U2OS cells with indicated protein siRNA knockdown in FANCA knock out background generated by CRISPR-Cas9. Comparison with 282-U2OS WT cells shows non-redundant relationship with FANCA during HR for FAAP20 and FANCD2. **d** Schematic of Δ7 RMR-U2OS reporter cell experiments to measure SSTR using short regions of homology. A non-homologous insert is removed from an interrupted GFP cassette through expression of CRISPR and dual sgRNAs that target the 5’ and 3’ recognition sites surrounding the sequence. GFP is restored through a targeted insertion at the break site using an exogenous single-strand oligonucleotide repair template with varying degrees of RNA content. For all RMR experiments, cells are first gated on mCherry due to a constitutively active mCherry construct present within the CRISPR expression plasmids, then as a % GFP within the mCherry (+) cell population. They are then reported as a % of a control condition (siCtrl or vector) that has been normalized to 1. **e** RMR using 100% DNA template with the indicated knockdown conditions. **f** RMR using a DNA/RNA hybrid repair template (H2) with 7 bp of RNA spanning the targeted insertion region needed to restore GFP. Indicated knockdown conditions are shown. All graphs in this figure show bars as mean values and error bars as SD. All statistical tests were two-tailed Student’s *t* test where *P < 0.05, **P < 0.01, ***P < 0.001, ****P < 0.0001; *n* = 3 for all experiments.

To assess the effect of FAAP20 protein on HR of DSBs, we performed siRNA knockdown of FAAP20 and the FANCA and FANCG subunits of the proposed AG20 subcomplex in 282 cells (Fig. 1a). We also performed siRNA knockdown of BRCA2 as a positive control to validate the cell line and provide a comparison with a bona fide HR factor. A siRNA pool consisting of (4) individual RNA sequences was used for each protein tested, as well as for the non-targeting siCtrl. After knockdown of each factor (Supplementary Fig. 1b), and subsequent I-SceI transfection and FACS analysis, we saw a near elimination of GFP+ cells after FAAP20 KD (Fig. 1a). Consistent with previous reports we observed a mild reduction with FANCA KD but we did not see a significant decrease in HR after FANCG KD. This conflicts with some other reports that show FANCG depletion affects HR^27,38^, but this can be explained when utilizing the information provided by the mCherry/I-SceI dual plasmid. If we were to observe the effect of FANCG KD on GFP+ cells based on the entire cell population, we observe a significant decrease in HR events (Supplementary Fig. 1c). However, when looking at the transfection efficiency of the I-SceI plasmid across all knockdown conditions by mCherry+ signal, FANCG KD causes a significant decrease in transfection efficiency compared with the siCtrl that obscures the true effect of FANCG loss on HR (Supplementary Fig. 1d). When transfection efficiency is normalized by gating GFP+ cells on mCherry+ cells, FANCG KD no longer creates a significant decrease in HR, showing that the transfection efficiency was creating a false positive result. On the other hand, FAAP20 KD also causes a significant decrease in transfection efficiency (Supplementary Fig. 1d), but when GFP+ signal is normalized to the mCherry+ signal, a significant and substantial decrease in HR is still observed, showing that the effect of FAAP20 KD on HR is truly due to the loss of protein and not I-SceI transfection efficiency. In fact, FAAP20 KD causes ~80–90% decrease in HR which is appreciable to the effect of BRCA2 KD on HR (90–100% loss of HR). To support that this HR loss is due to a specific effect of FAAP20, we also observed a slight arrest of 282 cells in G1 phase of the cell cycle that occurs only with FAAP20 KD (Supplementary Fig. 1e). These data indicate that FAAP20 is a major HR factor.

Next, we wanted to know if FAAP20’s effect on HR was comparable to FANCD2’s effect on HR, because FAAP20 can act upstream of FANCD2 to promote its ubiquitination and foci formation^20^. To test, we performed siRNA knockdown for FANCD2 using a siRNA pool in 282 cells, then transfected I-SceI, followed by FACS analysis and compared with FAAP20 KD under the same conditions. We see that FANCD2 depletion does cause a substantial decrease in HR events (~75–80% decrease), but HR events are still significantly higher in FANCD2 KD vs FAAP20 KD (Fig. 1b). While it is likely that FAAP20 acts in concert with FANCA and FANCD2 during certain HR events, our results suggest that FAAP20 has a slightly more prominent role than its FA partners.

Due to the profound decrease in HR observed with FAAP20 KD compared with FANCA, (Fig. 1a), it appears that FAAP20 and FANCA are not redundant in HR repair. However, previous studies have shown that FAAP20 and FANCG stabilize FANCA protein in cells while FANCA reciprocally stabilizes FAAP20 and FANCG^20, 24,39^). In our western blots, we can see reduced but considerable levels of FANCA in 282 cells with FAAP20 KD (Supplementary Fig. 1b). In the case of FAAP20, we detect residual FAAP20 in cells with FANCA KD, but the levels of FAAP20 are greatly reduced compared with the control (Supplementary Fig. 1b). Because of FAAP20’s known dependence on FANCA for protein stability, we wanted to further confirm that the role of FAAP20 in HR is truly non-redundant with FANCA and is not a result of insufficient FANCA KD with siRNA. To test, we utilized 282-U2OS cells with biallelic genomic FANCA knockout (KO) generated by CRISPR-Cas9. We first probed FAAP20 protein expression levels in 282 FANCA KO cells by western blot in siCtrl or siFAAP20-transfected cells. We were able to observe FAAP20 protein in FANCA KO cells that was completely depleted upon FAAP20 siRNA treatment, although bands representing FAAP20 protein are quite faint (Supplementary Fig. 2a). But nevertheless, we can still see a population of FAAP20 that exists in the absence of FANCA, and we were able to quantify this band relative to FANCA KO cells with FAAP20 KD using densitometry analysis (Supplementary Fig. 2a). Based on our densitometry analysis, we see a greater than 100-fold difference in FAAP20 protein levels between FANCA KO cells treated with siCtrl vs FANCA KO cells with FAAP20 KD. We then tested the effect of FAAP20 KD on HR events in 282 cells with FANCA KO and saw that FAAP20 KD causes a significant decrease in HR compared with siCtrl-treated FANCA KO cells, confirming that the effect of FAAP20 KD on HR is not equivalent to FANCA (Fig. 1c). In fact, we saw that FAAP20 KD in FANCA KO cells completely abolishes HR events, highlighting the essential nature of both FANCA and FAAP20 to this pathway. We also tested the effect of FANCD2 KD on HR in 282 FANCA KO cells to see if FANCD2 is redundant with FANCA in HR repair, as FANCD2 acts downstream of FANCA in the FA pathway. Similar to FAAP20 KD, we saw that FANCD2 KD causes a significant decrease in HR in 282 FANCA KO cells, even though FANCD2 KD cells have significantly higher amounts of HR compared with FAAP20 KD in 282 FANCA KO cells (Fig. 1c). These effects of FAAP20 and FANCD2 KD in 282 FANCA KO cells were not due to cell cycle arrest according to our cell cycle analysis (Supplementary Fig. 2b). These results further confirm that the roles of FAAP20 and FANCD2 are not redundant with FANCA in HR repair.

Recently, increasing studies are showing that HDR events can occur using exogenous single-stranded nucleic acid templates, and these targeted insertion events are dependent on slightly differing repair factors than canonical HR. This single-stranded template repair (SSTR) is emerging as a distinct subtype of HR and is particularly relevant for CRISPR-based genome editing^40,41^, Previously, it has been shown that FANCA is important for SSTR in K562 cells^40^, so we wanted to know if the HR role of FAAP20 extended into this pathway. To test, we utilized a U2OS cell-based GFP reporter system in the cell line DK71G-U2OS created in the lab of Dr. Jeremy Stark^37,42^. This system measures templated repeat-mediated repair (RMR) using a reporter construct with a GFP expression cassette interrupted by a non-homologous insert. This insert is excised using two CRISPR constructs, one with sgRNA targeting a 5’ site to the insert, and one with sgRNA targeting the 3’ site. To control for transfection efficiency, we added a constitutively expressed mCherry gene in both CRISPR-expressing plasmids. After removal of the non-homologous insert, the resulting DSB can be repaired with a co-transfected single-stranded nucleic acid repair template with 20 bp of homology on each arm to the repetitive sequence flanking the DSB site. The repair oligonucleotide also contains a 7 bp internal sequence that restores GFP expression after templated insertion (Fig. 1d). This allows for analysis of SSTR using FACS analysis. To test FAAP20’s role in this type of RMR, we performed FAAP20 KD and FANCG/FANCA KD for comparison. We then transfected both CRISPR/mCherry plasmids with the 47 bp ssDNA template and analyzed the % GFP cells. We observed that FAAP20 KD caused a large, significant decrease in templated repair using ssDNA, where FANCA and FANCG KD had no effect (Fig. 1e). Western blots in DK71G cells after FANCA KD show detectable levels of FAAP20, further supporting a specific role for FAAP20 in this pathway (Supplementary Fig. 2c). We also wanted to know if overexpression of FAAP20 affects the frequency of SSTR using a short homologous ssDNA template, so we repeated the RMR assay after transient overexpression of FAAP20 and did not observe its ability to upregulate SSTR above control levels (Supplementary Fig. 2d). This RMR reporter system has also been used to show that RNA-templated repair can effectively restore GFP expression by inserting ribonucleotides within the SSTR template^43^. Because cells can perform RNA-templated repair at DSBs, we wanted to test whether FAAP20 had any role in SSTR using ssRNA. We then designed (3) RNA-containing templates that were identical to the ssDNA template in both length and sequence composition but differed in the amount of RNA content as follows: 100% RNA, and H1 or H2 DNA/RNA hybrids with 9 bp RNA on each side of the 7 bp insertion sequence (18 bp RNA total) or with RNA as the entire 7 bp insertion sequence, respectively (Fig. 1d). First, we tested the efficiency of repair with each RNA-containing template and saw that only SSTR using H2 yielded measurable about of RNA templated repair events. H1 and 100% RNA templates demonstrated little and no repair events respectively (Supplementary Fig. 2e). Using the H2 template, we tested the effect of FAAP20 on RNA-templated repair by performing the RMR assay after FAAP20 siRNA KD. We observed that FAAP20 KD almost completely abolished RNA-templated repair using the H2 template (Fig. 1f). This likely means that the repair step coordinated by FAAP20 is important for both pathways of HR.

### FAAP20 promotes the single-strand annealing (SSA) subset of HDR through FANCA

**Fig. 2:**
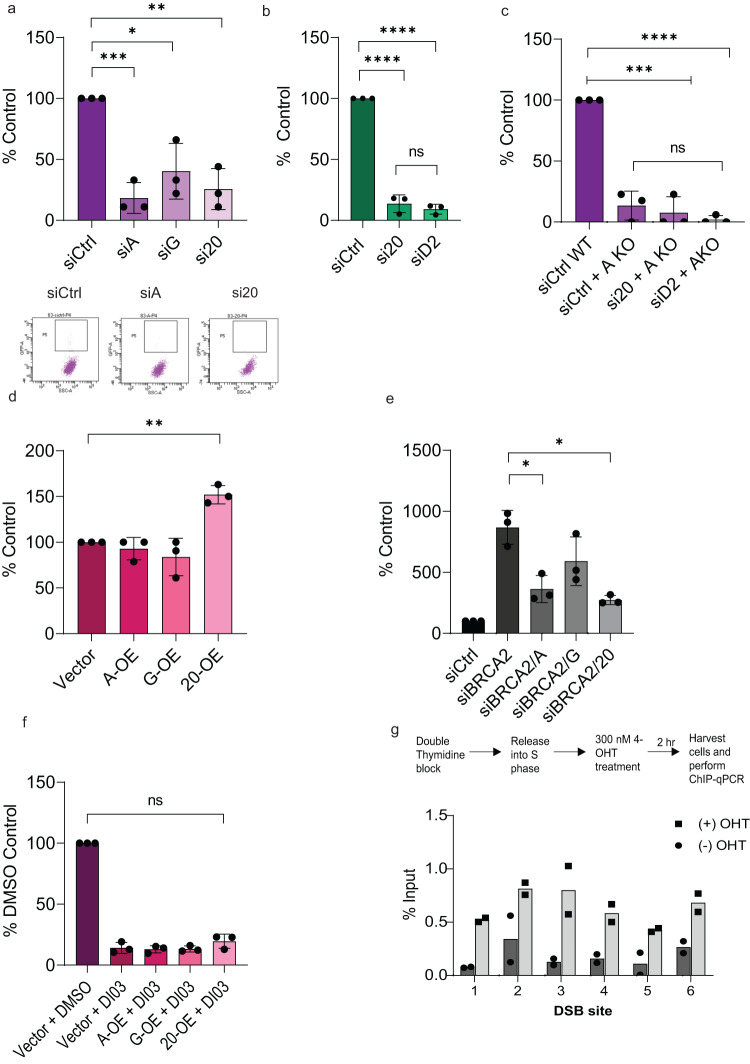
FAAP20 promotes the single-strand annealing (SSA) subset of HDR through FANCA. **a**, **b** Measure of SSA events at a defined DSB using 283-U2OS (SA-GFP) reporter cells with the indicated knockdown conditions. Events are reported as a % of siCtrl and representative dot plots are also shown. **c** Measure of SSA events in 283-U2OS WT cells compared with 283-U2OS FANCA KO cells that also were treated with the indicated siRNA knockdown conditions. **d** Identical to 4a except showing effects of the indicated overexpression conditions. Events are reported as a % of Vector control. **e** Measure of SSA events in 283-U2OS cells with knockdown conditions occurring simultaneously with BRCA2 knockdown. Events are reported as a % of siBRCA2 GFP %. **f** Measure of SSA events in 283-U2OS cells after overexpression of the indicated proteins, and subsequent treatment with a RAD52-specific chemical inhibitor DI03. Cells transfected with Vector and treated with drug vehicle (DMSO) were used as a control, but statistical comparisons for each overexpression group were done against Vector+DI03. 75 μM DI03 was used for each treatment group and was determined experimentally to inhibit most SSA events but preserve cell viability for downstream analysis. For graphs (**a**–**f**), bars represent means with error bars showing SD. For all experiments, two-tailed Student’s *t* tests were used for statistical analysis where ns = not significant, *P < 0.05, **P < 0.01, ***P < 0.001, ****P < 0.0001; *n* = 3 for all experiments **g** ChIP-qPCR in DIvA-U2OS cells with FANCA IP in untreated cells or treated with 4-OHT to induce DSB formation. Six (6) annotated AsisI recognition sites were chosen for amplification after FANCA pulldown using primers ~80 bp upstream from the predicted cut site. Gray bars indicate qPCR products from cells where DSB damage was induced with 4-OHT, where white bars represent products from cells without DSB induction. % Input values were calculated using the equation: % Input = 100/2^ ΔCt^ and were normalized to 5% chromatin input. One representative graph of 3 experimental repeats is shown here. Dots represent 2 technical repeats.

HR and SSA are both considered HDR pathways where HR consists of high-fidelity repair while SSA is error-prone and mutagenic. We then wanted to investigate if FAAP20 was involved in the SSA pathway of HDR in addition to HR and SSTR, especially since many HR factors also participate in SSA. Also, FAAP20 forms a strong, direct interaction with FANCA^20,24^ which has an established role in SSA of DSBs^44^ and could indicate that FAAP20 also has a role in this pathway. To test FAAP20’s role in SSA repair, we employed another established cell-based reporter (SA-GFP) that measures SSA events at an I-SceI-induced DSB in the cell line 283-U2OS. In these cells, we performed FAAP20 KD as well as KD of its binding partners FANCA and FANCG (Supplementary Fig. 3a). We observed that FANCA, FANCG, and FAAP20 KD conditions all caused a significant decrease in SSA events that did not differ significantly from each other (Fig. 2a). These KD effects on SSA were also not due to alterations in cell cycle distribution based on our analysis (Supplementary Fig. 3b). We also tested the effects of FANCD2 KD on SSA compared with FAAP20 KD and observed that FANCD2 KD resulted in comparable decreases in SSA that were not significantly different from the effect of FAAP20 KD (Fig. 2b). To confirm that the effects of FAAP20 and FANCD2 KD on SSA were not redundant with FANCA, we also tested FAAP20 and FANCD2 KD in 283 cells with a FANCA biallelic genomic KO generated by CRISPR-Cas9, similar to our experiments in 282 FANCA KO cells (Supplementary Fig. 3c). In 283 FANCA KO cells, we do not see any further decrease in SSA events with simultaneous FAAP20 or FANCD2 KD, confirming that the roles of FANCA, FAAP20, and FANCD2 appear to be common among each other in SSA repair (Fig. 2c).

We then wanted to know if overexpression of AG20 proteins could upregulate SSA repair, which may prove useful when studying tumors who rely on the SSA pathway for chemo/radiotherapy-resistance. To test, we overexpressed all FANCA, FANCG, or FAAP20 protein in 283 cells and saw that increased FAAP20 alone but not FANCA or FANCG causes a significant increase in SSA repair events (Fig. 2d). Although FANCA and FAAP20 have equivalent roles in SSA, but only FAAP20 expression can increase FANCA, this may be explained by the previous finding that high expression levels of FAAP20 can boost amounts of cellular FANCA through protein stabilization^45^. Consistently, we see that FAAP20 overexpression increases FANCA protein level, while FANCA overexpression does not cause a readily detectable change in FAAP20 (Supplementary Fig. 3d). Additionally, overexpressed FAAP20 protein is stable and able to translocate to the nucleus even in the absence of FANCA, as seen by nuclear fractionation and western blot in 283 FANCA KO cells with FAAP20 overexpression (Supplementary Fig. 3e). Because FANCA is not necessary for FAAP20 nuclear recruitment, only a small increase in FANCA may be needed to support the role of FAAP20 in SSA.

Because we observed that FAAP20 and its binding partners directly participate in SSA, we then wanted to know how these proteins contribute to upregulated SSA repair in BRCA2-deficient cells. This mechanism is relevant in BRCA2-deficient cancers that have been shown to increase SSA pathway use due to abrogation of HR^46^. To test how FAAP20 and its binding partners contribute to upregulated SSA in BRCA2-deficient cells, we first performed BRCA2 KD in 283-U2OS cells and observed the characteristic SSA increase that is 8-10-fold over the control (Fig. 2e). We then performed KD of FANCA, FANCG, and FAAP20 simultaneously with BRCA2 KD to see if loss of AG20-mediated SSA causes the SSA increase caused by BRCA2 loss. From this, we see that FANCA or FAAP20 KD results in a significant SSA decrease, whereas FANCG KD does not (Fig. 2e). These results show that FAAP20 and FANCA contribute to the upregulation of SSA events in BRCA2-deficient cell backgrounds.

Cellular BRCA2 loss is synthetically lethal with the loss of RAD52’s single-strand annealing activity^18,47,48^, Because FAAP20/FANCA-mediated annealing and RAD52 both have such strong roles in SSA, we wanted to see if FAAP20/FANCA could serve as a potential compensatory mechanism for RAD52-mediated annealing. To test, we employed the RAD52 small molecule inhibitor DI03 developed by Dr. Alexander Mazin’s lab that specifically inhibits the biochemical annealing of RAD52 but does not affect damage-induced RAD51 loading^18^. By treating 283 cells with DI03 when DSBs are induced, we see that DI03 does reduce SSA repair (Fig. 2f). To see if AG20 proteins could overcome SSA loss from RAD52 inhibition, we overexpressed each protein in 283-U2OS cells prior to DI03 treatment and damage induction. However, overexpression of FANCA, FANCG or FAAP20 proteins was not able to cause a significant increase in SSA repair above vector control during DI03 treatment (Fig. 2f). This is likely not due to a trapping effect of RAD52 because DI03 inhibits RAD52 nuclear foci^18^. We also know that the results are not caused by off-target inhibition of FANCA’s annealing activity, as the addition of DI03 compound to in vitro single-strand annealing assays does not affect FANCA’s annealing activity (Supplementary Fig. 4a). These results suggest that during SSA repair, swapping out one annealing protein for another cannot occur by overexpression of a biochemically similar protein.

So far, we have only shown indirect evidence for FANCA’s participation in DSB repair through cell-based reporters, but not direct interaction with DSB damage in cells. To provide stronger evidence that recombinase activity of FANCA is used directly on broken DNA ends in a chromatin context, we performed ChIP-qPCR in the DIvA-U2OS cell system. In these cells, ~200 AsiSI enzyme recognition sites are stably integrated throughout the genome, allowing for inducible DSB damage in chromatin at known genomic locations. This allows measurement of FANCA binding at DSB sites by immunoprecipitating FANCA with a specific antibody and performing qPCR with primers that amplify genomic regions 80 bp upstream from the predicted AsiSI cute site (primers designed by Dr. Gaelle Legube’s group^49^). We also performed this experiment after synchronizing the cells with double thymidine block and releasing into S phase in order to maximize the capture of FANCA on chromatin, as the FA pathway is highly active during S/G2 phases of the cell cycle^50^. We observed that FANCA association with these designated chromatin regions was enhanced in a DSB damage-induced manner (Fig. 2g), compared with FANCA’s association with undamaged chromatin. This trend was apparent when analyzing qPCR data by two different types of calculations (% Input and fold enrichment) (Supplementary Fig. 4b–g). Together, we see from these results that the activities of FAAP20 in the SSA repair pathway require FANCA and may involve direct mediation of repair steps based on FANCA’s ability to bind broken DNA ends on chromatin.

### FAAP20 supports SSA in part through stimulation of FANCA’s biochemical activity

**Fig. 3:**
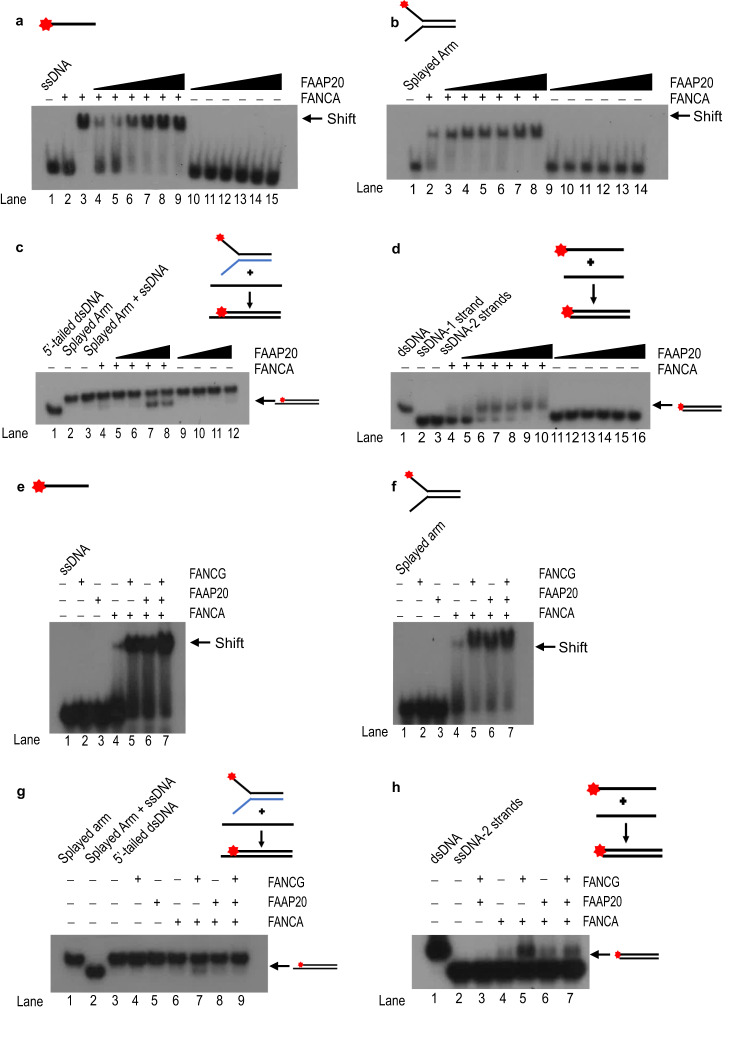
FAAP20 supports SSA in part through stimulation of FANCA’s biochemical activity. **a** ssDNA EMSA of recombinant FAAP20 protein titrated against fixed, suboptimal FANCA protein or alone and 1 nM substrate. Lane 1: No protein (PBS/10%BSA); lane 2: suboptimal, 10 nM FANCA alone; lane 3: positive control of high concentration-80 nM FANCA, lanes 4–9: FAAP20: 9–24 nM in 3 nM increments, and FANCA: 10 nM; lanes 10–15: FAAP20 only: 9–24 nM. **b** Splayed arm EMSA analysis of recombinant FAAP20 titrated against fixed, suboptimal FANCA protein using 1 nM substrate (0.5 nM each strand). Lane 1: No protein; lane 2: FANCA only: 10 nM; lanes 3–8: FAAP20 :9–24 nM in 3 nM increments, and FANCA: 10 nM; lanes 9–14: FAAP20 only: 9–24 nM. **c** Strand exchange assay with FAAP20 recombinant protein titrated against fixed, suboptimal FANCA. Strand exchange of splayed arm substrate occurs with ssDNA that is complementary to the 5’ ^32^P-labeled strand, resulting in a 5’-tailed dsDNA product. Lane 1: No protein positive control with 5’-tailed dsDNA product, lane 2: No protein negative control with splayed arm substrate, lane 3: No protein control with labeled splayed arm and cold ssDNA complementary strand, lane 4: suboptimal, 10 nM FANCA only, lanes 5–8: FAAP20: 5–30 nM (5, 10, 20, 30 nM), and FANCA: 10 nM; lanes 9–12: FAAP20 only: 5–30 nM. **d** Single-strand annealing of two complementary oligonucleotides with FAAP20 protein titrated against a fixed, sub-optimal concentration of FANCA and a fixed concentration of DNA substrate (0.5 nM each strand). Lane 1: pre-annealed duplex DNA serving as a positive control; lane 2: ssDNA only; lane 3: No protein control with both strands; lane 4: FANCA only: 10 nM; lanes 5–10: FAAP20: 5–30 nM in 5 nM increments, and FANCA: 10 nM; lanes 11–16: FAAP20 only: 5–30 nM. **e** ssDNA EMSA comparing fixed and suboptimal equimolar concentrations of all three AG20 recombinant proteins. Lane 1: No protein; lane 2: FAAP20 only: 10 nM; lane 3: FANCG only: 10 nM; lane 4: FANCA only: 10 nM; lane 5: FANCA and FANCG: 10 nM each; lane 6: FANCA and FAAP20: 10 nM each; lane 7: FANCA, FANCG, and FAAP20: 10 nM each. 1 nM substrate was used for each reaction. **f** EMSA that is identical in experimental conditions and layout to Fig. 1e, except a splayed arm substrate is used instead of ssDNA. **g** Strand exchange assay using the same DNA substrates as Fig. 1c but tests the activity of equimolar concentrations of AG20 proteins. Lane 1: negative control; lane 2: positive control with splayed arm substrate; lane 3: No protein control with both substrates present; lane 4: FANCG only: 10 nM; lane 5: FAAP20 only: 10 nM; lane 6: FANCA only: 10 nM; lane 7: FANCA and FANCG: 10 nM each; lane 8: FANCA and FAAP20: 10 nM each; lane 9: FANCA, FANCG, FAAP20: 10 nM each. **h** Single-strand annealing assay similar to 1d, but testing equimolar concentrations of AG20 proteins. Lane 1: pre-annealed positive control; lane 2: No protein control with both oligonucleotides; lane 3: FANCG and FAAP20: 10 nM each; lane 4: FANCA only: 10 nM; lane 5: FANCA and FANCG: 10 nM each; lane 6: FANCA and FAAP20: 10 nM each; lane 7: FANCA, FANCG, and FAAP20: 10 nM each. For all experiments, *n* = 3 with one representative gel shown for each experiment.

Because FAAP20 works with FANCA to promote the SSA pathway and FANCA makes direct contact with genomic DSBs, we then hypothesized that part of the role of FAAP20 in SSA repair is to modulate the biochemical activity of FANCA. This hypothesis is also supported by the ability of FANCG, another binding partner of FANCA, to modulate FANCA’s biochemical activity^44^. Therefore, we aimed to test the effects of FAAP20 protein on FANCA’s biochemical nucleic acid binding, annealing, and strand exchange functions. To test, we purified human recombinant FANCA (Supplementary Fig. 5a) and FAAP20-6xHis protein (Supplementary Fig. 5b) from Hi5 insect cells using the bac-to-bac expression system. We then performed electromobility shift analysis (EMSA) by titrating recombinant FAAP20 protein against a fixed, suboptimal concentration of FANCA protein, and 1 nM ^32^P-labeled ssDNA substrate that is 61 bp in length. Reactions were run on a non-denaturing polyacrylamide gel, where it was observed that FAAP20 protein increases FANCA’s affinity for ssDNA in a concentration-dependent manner, whereas FAAP20 itself is not able to bind to ssDNA (Fig. 3a). We also performed an EMSA with a similar experimental setup using FAAP20 and FANCA protein, but with a splayed arm DNA substrate due to FANCA’s inherent binding preference for splayed arm structures^51^. FAAP20 was also able to stimulate FANCA’s binding to a splayed arm structure in a concentration-dependent manner (Fig. 3b). We also observed the ability of FAAP20 to stimulate FANCA binding of dsDNA (Supplementary Fig. 5c) and ssRNA (Supplementary Fig. 5d) showing that FAAP20 does not enforce any substrate specificity but increases FANCA’s inherent affinity for whichever nucleic acid substrate is available. We then wanted to know if FAAP20’s ability to stimulate substrate-binding of FANCA indicated FAAP20’s ability to stimulate the biochemical DNA processing activities of FANCA. To test, we performed a strand exchange assay in vitro using a splayed arm structure with one strand labeled with ^32^P on its 5’ end, and a cold oligonucleotide containing complementary sequence to that of the splayed arm. Strand exchange occurs when FANCA destabilizes the splayed arm substrate and reanneals the labeled strand with the complementary ssDNA, resulting in a smaller 5’-tailed dsDNA product that is visible as the lower band on a native polyacrylamide gel. Titration of FAAP20 protein against a fixed, suboptimal concentration of FANCA showed that FAAP20 was able to stimulate strand exchange activity of FANCA, while FAAP20 itself showed no activity (Fig. 3c). We also performed a single-strand annealing assay in vitro using complementary DNA oligonucleotides that are 74 bp in length, with one ^32^P-labeled strand and one cold strand. When annealed, these strands result in a larger duplex DNA product that migrates more slowly on a native gel than ssDNA. In a manner similar to the strand exchange assay, increasing concentrations of FAAP20 were able to stimulate the single-strand annealing activity of a fixed, suboptimal concentration of FANCA while FAAP20 does not perform this activity itself (Fig. 3d). Together, these results indicate that FAAP20 has an important role in supporting the recombinase activities of FANCA that are utilized during DNA repair in cells.

Because FAAP20 stimulates FANCA’s biochemical activities in a manner similar to FANCG and because FAAP20 is proposed to form the AG20 sub-complex with FANCA we wanted to know if the presence of all three AG20 proteins would synergistically enhance DNA binding and processing activities of FANCA when present in the same reaction mixture. We first tested this possibility using EMSA with a ssDNA substrate, and incubated with sub-optimal, equimolar concentrations of AG20 compared with A20, AG, or FANCA alone. We observed that AG and A20 had increased ssDNA affinity over FANCA alone as expected (Fig. 3e lanes 4–6), the addition of all three AG20 proteins did not drastically increase binding above AG or A20 (Fig. 3e, lane 7). The modest increase in DNA binding in the AG20-containing reaction mixture appears to be slightly additive rather than synergistic in nature. We also tested EMSA with a splayed arm structure using the same reaction conditions as the EMSA with ssDNA, and still did not observe any synergistic activity from AG20 proteins (Fig. 3f). Using the same strand exchange and annealing assays used to test A20, we tested whether the presence of all three AG20 proteins resulted in a synergistic increase in biochemical activity. Yet like the EMSA experiments, equimolar concentrations of AG20 proteins were not able to stimulate strand exchange (Fig. 3g) or strand annealing activity (Fig. 3h) above levels seen with A20 or AG alone. The inability of AG20 proteins to further stimulate FANCA’s biochemical activity beyond the capacity of either individual binding partner may indicate that simultaneous binding of FANCA to FANCG and FAAP20 is not required for its DNA processing roles. These data suggest that FAAP20 has repair roles with FANCA that are separable from FANCA’s repair roles with FANCG, and the repair roles of FAAP20 with FANCA seem to be preferentially utilized in the SSA pathway.

### FAAP20’s role in HDR occurs downstream of BRCA1’s and 53BP1’s pathway decision step

**Fig. 4:**
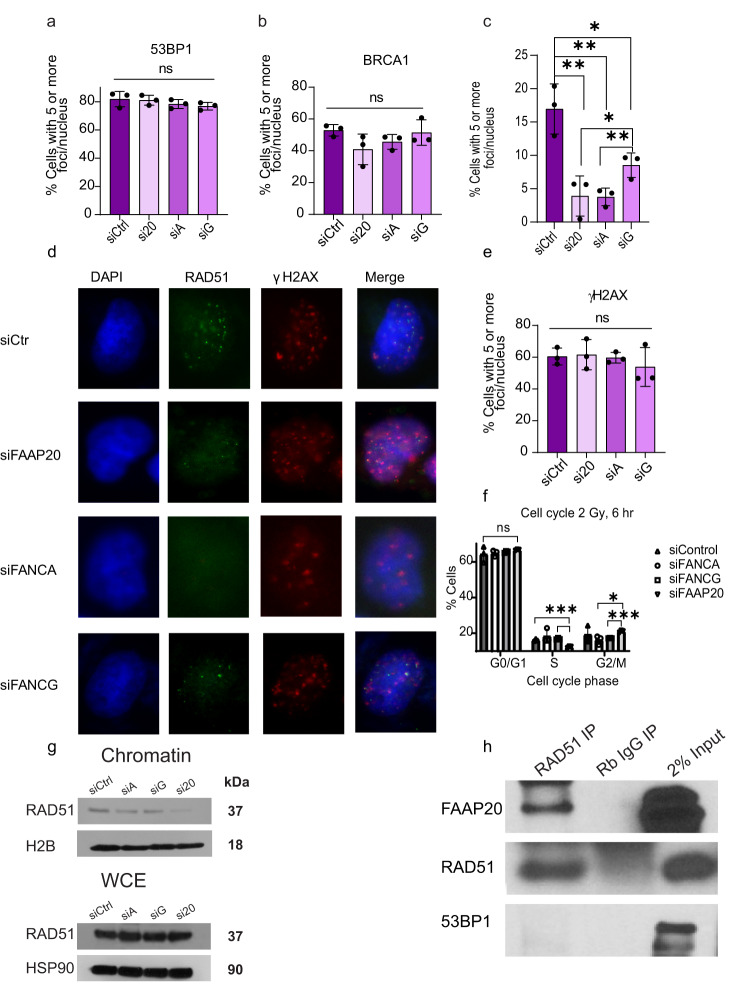
FAAP20’s role in HDR occurs downstream of BRCA1’s and 53BP1’s pathway decision step. **a** Quantification of 2 Gy IR-treated cells with indicated knockdown conditions containing 5 or more 53BP1 foci reported as a % of total cells analyzed. **b** Identical to 5a but showing quantification of BRCA1 IRIF foci. **c** Identical to Fig. 5a, b but showing quantification of RAD51 IRIF foci. **d** Representative cell images from immunofluorescent (IF) staining in U2OS cells. Cells were transfected with the indicated siRNAs and then treated with 2 Gy IR 6 h before harvesting and staining. RAD51 is recognized by anti-rabbit Alexa-488; γH2AX antibody is recognized by anti-mouse Alexa-594; and cell boundaries were determined with DAPI signal. Scale bars are shown at 10 μM. **e** Identical to Fig. 6a, b, d but showing quantification of γH2AX IRIF. **f** Cell cycle distribution analysis by propidium iodide (PI) staining in U2OS cells with the same treatment conditions as IF staining conditions shown in Fig. 6a–e. For graphs (**a**–**c**, **e**, **f**), bars show mean with error bars as SD. Two-tailed Student’s *t* test were used for statistical analysis where ns = not significant, *P < 0.05, **P < 0.01, ***P < 0.001, *n* = 3 for all experiments. **g** Chromatin fractionation and western blotting of chromatin fraction vs whole cell extract for RAD51 with the indicated protein knockdown. Cell conditions matched those used in IF staining experiments (2 Gy IR-treated U2OS cells, harvested after 6 h). 40 μg protein was loaded for each sample. H2B protein was used as a loading control for the chromatin-bound fraction, while HSP90 was used as a loading control for whole cell extract. One representative gel from three individual experiments is shown. **h** Coimmunoprecipitation in HEK293t whole cell extracts that overexpress WT FAAP20, using anti-RAD51 IgG and with Rb IgG isotype control. 53BP1 was probed as a negative control. *n* = 3.

HR and SSA share common early steps, but then diverge into their own respective pathways after the initiation of end resection^52^. We then wanted to know more mechanistic detail about when FAAP20 exerts its role in promoting HDR repair. To test, we performed immunofluorescent (IF) staining for established DSB repair factors in cells with FAAP20 KD 6 h after IR treatment. We first performed staining for BRCA1 and 53BP1 IRIF because these proteins have antagonistic roles with each other in either promoting end resection-based pathways via BRCA1, or non-homologous end joining (NHEJ) via 53BP1. This means that an alteration in the levels of each protein that corresponds to FAAP20 loss could indicate that FAAP20 participates in the early steps of promoting HDR over NHEJ^53–55^. Upon staining for these factors in each KD condition, we did not observe any significant differences in 53BP1 or BRCA1 IRIF after quantification of cells with 5 or more foci (Fig. 4a, b, respectively). This indicates that the major role of FAAP20 and its binding partners in DSB repair is likely not in early DSB pathway decision steps.

Because we did not see changes in 53BP1 or BRCA1 IRIF with FAAP20 KD, we then wanted to see if downstream HR repair was affected. We performed IF staining for RAD51 IRIF, a specific HR marker that is mutually exclusive to SSA. All KD conditions (A/G/20) showed significantly reduced RAD51 IRIF compared with the control, whereas FAAP20 and FANCA knockdown had a slightly more significant effect than FANCG knockdown (Fig. 4c, d). The reduction in RAD51 foci was not due to differences in damage quantity or signaling, as γH2AX IRIF was not significantly changed among the various knockdown conditions (Fig. 4e). The RAD51 IRIF decrease also cannot be accounted for based on cell cycle differences that would block HR repair, because propidium iodide staining of U2OS cells under the same experimental conditions did not show any arrest or accumulation of cells in G1/G0 that would prevent HDR pathways, although FAAP20 KD did result in a significant decrease in S phase cells (Fig. 4f). To verify the observed reduction of RAD51 IRIF occurring with AG20 knockdown, we performed chromatin fractionation and western blotting of the chromatin-bound fraction for RAD51 under identical treatment conditions as IF staining experiments. Consistent with our IF staining results, western blotting of U2OS chromatin-fractions showed that knockdown of each AG20 protein causes a detectable decrease in RAD51, particularly in the case of FAAP20 KD. However, RAD51 global protein levels in whole cell extracts from the same treatment conditions are not affected by knockdown of FAAP20, FANCA, or FANCG (Fig. 4g). To further substantiate a direct relationship between FAAP20 and RAD51, we performed coimmunoprecipitation (co-IP) by pulling down RAD51 using a specific anti-RAD51 antibody in HEK293t cells. FAAP20 protein was not detectable in our parental HEK293t input samples due to low protein abundance and excessive dilution, so to probe FAAP20 interaction we overexpressed FAAP20 protein in HEK293t cells prior to co-IP. Western blotting showed that FAAP20 was present in RAD51 IP’d fractions and was not present in rabbit IgG isotype control fractions (Fig. 4h). We also probed for 53BP1 in RAD51 IP’d fractions as a negative control and did not see any 53BP1 pull down with anti-RAD51 antibody, as expected (Fig. 4h). This result shows that FAAP20 protein interacts with RAD51 in cells, further validating that FAAP20 works together with RAD51 in a common function.

### HDR functions of FAAP20 support cell proliferation

**Fig. 5:**
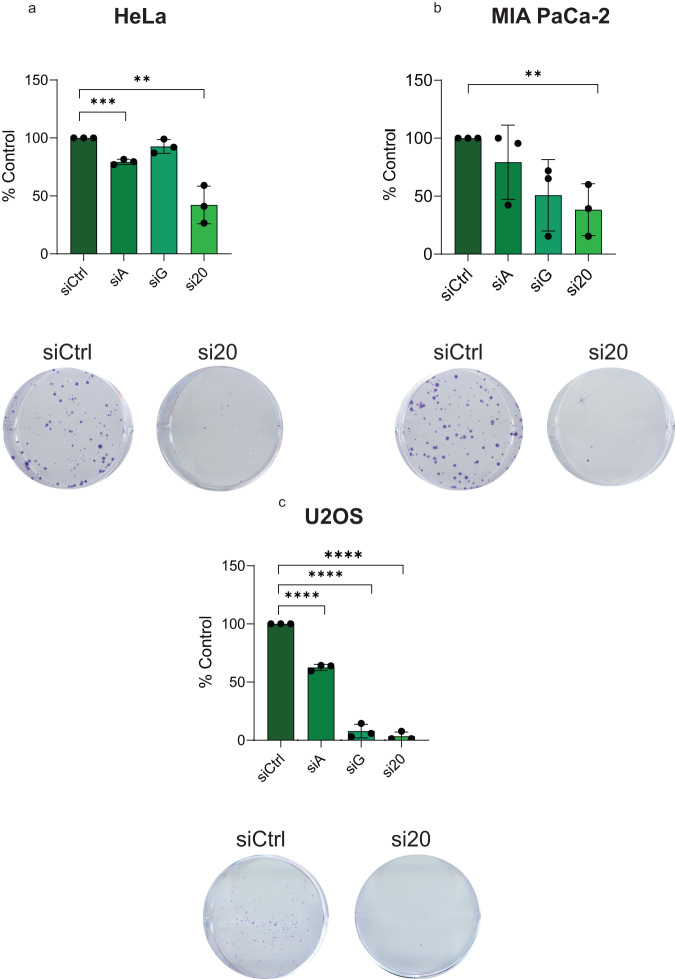
HDR functions of FAAP20 support cell proliferation. **a** Quantification of colonies in untreated HeLa cells with the indicated proteins knocked down, shown as a % of siCtrl. Images of siFAAP20 vs. siCtrl are shown. **b** Quantification of colonies in untreated MIA PaCa-2 cells with the indicated proteins knocked down, shown as % siCtrl. Images of siFAAP20 vs. siCtrl are shown. **c** Quantification of colonies for indicated proteins knocked down in U2OS cells, shown as a % of siCtrl. For (**a**–**c**), bars in graphs represent the mean with error bars showing SD. Statistical analysis for all graphs was done by two-tailed Student’s *t* test where **P < 0.01, ***P < 0.001, ****P < 0.0001; *n* = 3 for all experiments.

Repair factors that participate in HDR pathways are important for ensuring complete genome duplication and cellular progression through S/G2 cell cycle phases. This role is particularly important for cancer cell proliferation where DNA replication must progress through an unstable genome^9,56^. To see how FAAP20’s role in HDR contributes to cancer cell proliferation, we performed clonogenic survival assays in three cancer cell lines: HeLa, MIA PaCa-2, and U2OS. First, we performed FAAP20 siRNA KD in unperturbed HeLa cells and observed ~50% less colony growth compared with siCtrl-treated HeLa cells (Fig. 5a). Interestingly, FANCA siRNA KD showed a small but significant decrease in colony formation while FANCG siRNA KD did not create a significant difference (Fig. 5a) which closely mimics the effects of these KD conditions on HR in 282-U2OS cells (Fig. 1a). We then performed the same KD of FAAP20, FANCA, and FANCG in U2OS and MIA PaCa-2 cells followed by colony formation. Across all three cell lines we see that FAAP20 KD causes a significant reduction in colony formation, while the effects of FANCA and FANCG KD are inconsistent across multiple cell lines (Fig. 5b, c). These results highlight the prominent role of FAAP20 in HR that supports DNA replication and cell proliferation.

### Loss of FAAP20 results in cellular radiosensitivity and PARPi sensitivity

**Fig. 6:**
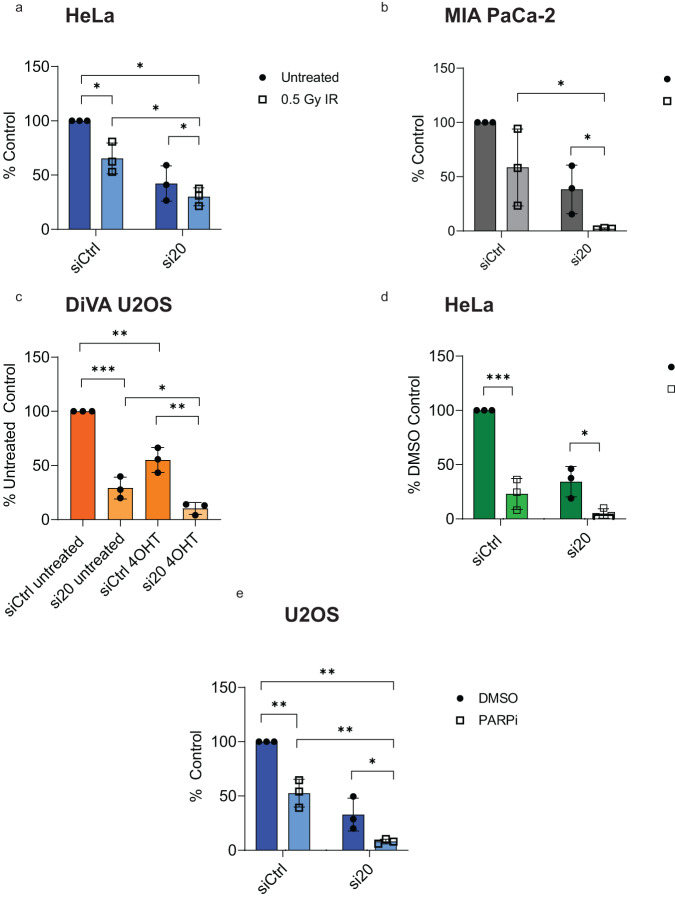
Loss of FAAP20-mediated HDR roles results in cellular radiosensitivity and PARPi sensitivity. **a** Quantification of colonies in HeLa cells with indicated siRNA knockdown, comparing untreated cells with 0.5 Gy IR treatment, shown as % of untreated siCtrl. **b** Comparison of MIA PaCa-2 colony formation in untreated cells vs 0.5 Gy IR with the indicated siRNA knockdown conditions and reported as % of untreated siCtrl. **c** Quantification of cell proliferation in DiVA-U2OS cells treated with 4-OHT compared with cells treated with vehicle control (Ethanol), and the indicated knockdown condition. Cell counts are shown as % of untreated siCtrl. **d** Quantification of HeLa cell colonies treated with PARP inhibitor AZD2461 compared with cells treated with DMSO vehicle. Results are reported as % of DMSO-treated control Quantification of cell proliferation in U2OS cells with the indicated knockdown conditions and treated with PARP inhibitor AZD2461. Results are reported as a % of DMSO-treated control. **e** Quantification of cell proliferation in U2OS cells treated with PARPi compared with cells treated with DMSO, and the indicated knockdown condition. Cell counts are shown as % of untreated siCtrl + DMSO. For all experiments shown here: bars show mean with error bars as SD. Two-tailed Student’s *t* test were used for statistical analysis where ns = not significant, *P < 0.05, **P < 0.01, ***P < 0.001, *n* = 3.

Cellular proficiency in DSB repair can guide radiotherapy response in cancer treatment while HDR repair proficiency can determine PARPi sensitivity^57–59^. Due to these immediate clinical implications associated with FAAP20’s repair roles, we wanted to know how the ability of FAAP20 to mediate HDR of DSBs contributed to both radiosensitivity and PARPi sensitivity. To test, we measured cell proliferation in HeLa, MIA-PaCa-2, and U2OS cells in response to IR treatment and PARPi. First, we wanted to know if the loss of FAAP20 protein causes sensitization to treatment with IR due to its apparent role in DSB repair. We performed siRNA KD (Supplementary Fig. 6a–c) prior to treatment with 0.5 Gy IR that has little effect on WT cells (siCtrl). However, with KD of FAAP20 we observed an additional loss of colony formation in cells treated with 0.5 Gy IR, when comparing colony number to treatment groups with FAAP20 KD alone in both HeLa cells (Fig. 6a) and MIA PaCa-2 cells (Fig. 6b). This result shows that the loss of FAAP20 can cause sensitization to IR treatment. Because U2OS cells were hypersensitive to FAAP20 KD in our traditional colony formation assay format, we seeded at higher density for this cell line to measure proliferation. Also, we found that our U2OS cells were hypersensitive to IR treatment and caused almost complete loss of cell growth, which made it impossible to test any further effects of FAAP20 KD. To overcome this and test the effect of FAA20 KD on DSB damage sensitivity in U2OS cells, we performed the same proliferation assay in DiVA-U2OS where we could induce widespread DSB damage with 4-OHT treatment. In DiVA-U2OS cells, we saw that FAAP20 KD (Supplementary Fig. 6d) caused significant reduction in cell proliferation when treated with 4-OHT compared with DiVA-U2OS cells with FAAP20 KD and treated with ethanol vehicle control (Fig. 6c). These results across multiple cell lines show that the loss of FAAP20 causes sensitivity to DSBs in cancer cells. Next, we wanted to know how the role of FAAP20 in HR would translate into PARP inhibitor (PARPi) sensitivity because the loss of HR proteins is synthetically lethal with PARPi^60–62^. So far, the impact of PARPi on core complex FA proteins is not well-studied but generally indicates sensitivity^63–66^. To test the effects of PARPi on FAAP20, we performed clonogenic survival in HeLa cells and cell proliferation assays in U2OS cells after siRNA KD of FAAP20, and subsequent treatment with AZD2461, a PARP1/2 inhibitor^67^. We were not able to perform this experiment in MIA PaCa-2 cells, as too much cell growth was lost with FAAP20 KD + DMSO, so that additional cell loss with PARPi could not be determined. In HeLa and U2OS cells, however, we were able to see that FAAP20 KD nearly abolished colony formation when treated with PARPi (Fig. 6d, e). By comparing DMSO-treated cells with each individual KD condition, we see that the KD alone is not responsible for the complete loss of cell growth. We also saw that treatment with RAD52 inhibitor DI03 caused additional loss of cell growth in U2OS cells with FAAP20 KD, compared with DMSO + FAAP20 KD (Supplementary Fig. 7a). This further strengthens a role for FAAP20 in HR as loss of other HR factors are also shown to cause sensitivity to RAD52 inhibition (RAD52i) in cancer cells^47,48^. Nevertheless, our experiments in FAAP20-deficient cancer cells show that FAAP20’s ability to support HDR repair pathways leads to its ability to maintain cell growth and proliferation during IR treatment, PARPi, and RAD52i.

## Discussion

**Fig. 7:**
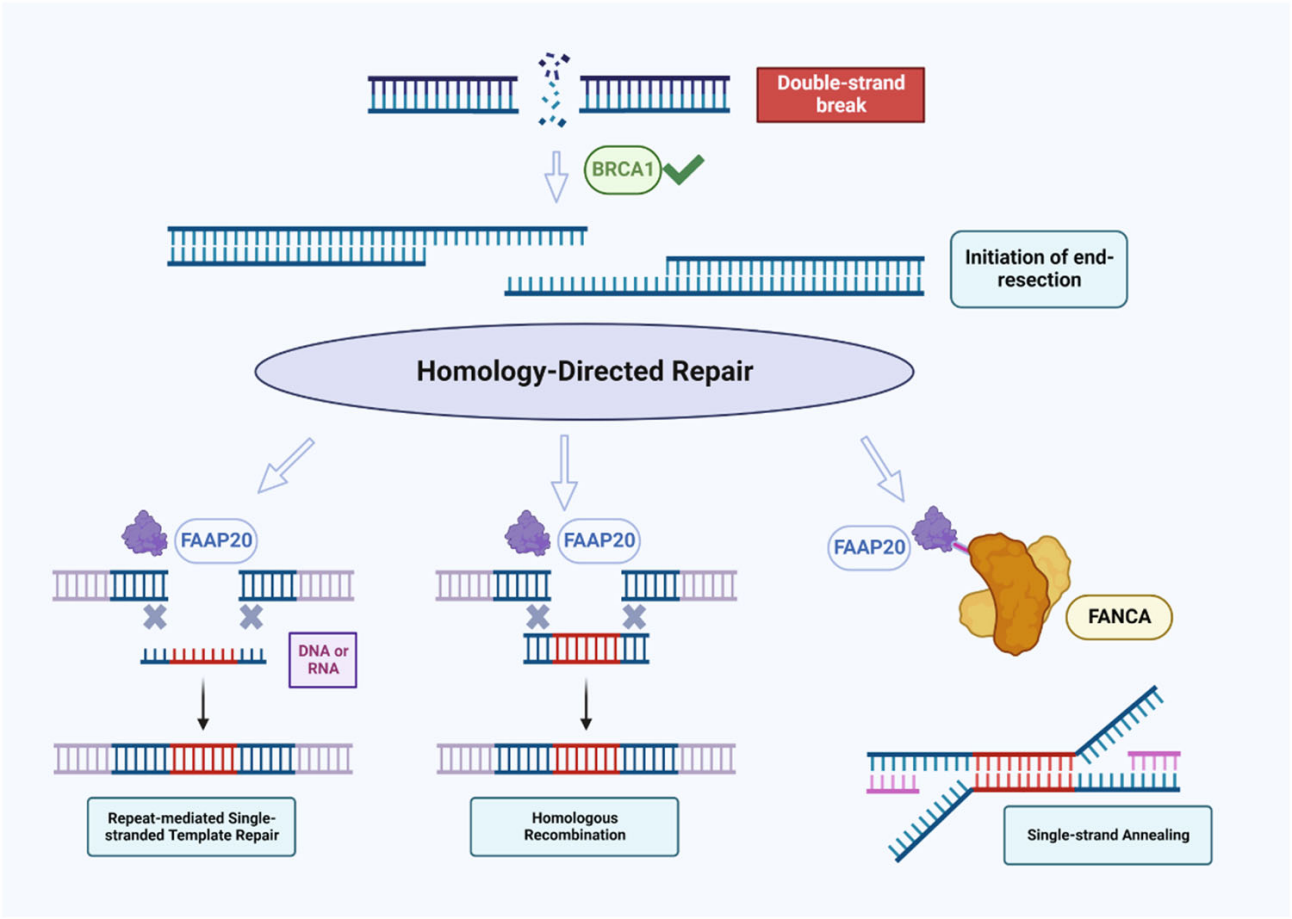
Model for FAAP20 participation in homology-directed repair of DNA double-strand breaks. After genomic DSB damage occurs, BRCA1 helps commit repair to HDR pathways by promoting end-resection. FAAP20 is then able to facilitate canonical HR using a double-strand DNA template, or SSTR using a ssDNA or ssRNA template. If HR is compromised in some way, FAAP20 can work with its FA binding partner FANCA to promote the error-prone SSA pathway. Image created with BioRender.com.

FAAP20 protein has previously been linked to ICL repair and promotion of FA pathway activation through ID2 monoubiquitination, but not to canonical DSB repair. Here, we demonstrate that major HDR DSB repair pathways are dependent on FAAP20. In the case of HR, this dependency seems to be non-redundant with FAAP20’s binding partner FANCA, while FAAP20’s role in the SSA pathway does appear to be shared with FANCA (Fig. 7). Prior work has shown that most FA proteins have a modest role in HR at DSBs^27, 28,30^, but we found the impact of FAAP20 loss on HR appears to be comparable to BRCA2 and much more substantial than its known FA core complex binding partners FANCA and FANCG. Unlike the roles of other FA proteins in HR that are heavily tied to replication and ICL-coupled HR^26^, we show FAAP20 does not require replication-coupling or ICL formation to influence HR outcomes. This points to a role for FAAP20 in HR that may act outside the canonical FA repair pathway. Yet from the emergence of new subpathways of HR^68–70^, and only partial requirements of certain factors to support HR events, it appears that HR repair cannot be rigidly defined. Based on the chromatin context, nucleic acid substrates, and repair factor availability, HR could be adaptable in utilizing different protein factors to tailor the biochemical steps needed to complete repair. This flexible view of HR could explain our finding that FAAP20, but not FANCA or BRCA2^42^ is important for SSTR in the RMR reporter system that uses a short repair template in U2OS cells. Perhaps both the template length and cell background are determinants of the exact repair factors chosen to participate in SSTR. Based on our observations that FAAP20 can support canonical HR, repeat mediated SSTR, and cell proliferation; it is possible that FAAP20’s role in HR may be common among these pathways. While the exact function of FAAP20 in these HDR processes will be the focus of future work, our data does provide some clues. From IF staining experiments in U2OS cells after IR treatment, the loss of FAAP20 does not alter BRCA1/53BP1 IRIF but reduces RAD51 IRIF. These results show a timing window of when FAAP20 supports HR: after the promotion of end resection-based repair, but prior to RAD51 loading. These steps were narrowed to the initiation of end-resection and RPA-exchange with RAD51, until we performed nuclear fractionation in DiVA-U2OS cells after FAAP20 KD and 4-OHT treatment and saw that FAAP20 KD did not affect nuclear levels of BRCA2 or RPA32 (Supplementary Fig. 7b). This even further narrows the window of FAAP20’s repair role to directly after RPA loading, and before its exchange with RAD51. Because FAAP20 binds to ubiquitin chains created by RNF8^21^, future studies that explore FAAP20’s associations with RNF8-ubiquitinated targets could provide further mechanistic details into FAAP20’s ability to support HR at DSBs.

Another observed FAAP20-specific function in SSTR involved the use of ssRNA as a repair template. Storici et al.^71^ have shown that RNA is used as a template for HDR particularly at sites of active transcription, though it is likely that RNA substrates are degraded at the damage site by RNase H1 and RNase H2 as previously proposed^72–76^. This is consistent with our initial substrate tests using the RMR system in U2OS cells, where it was apparent that repair efficiency was inversely proportional with the amount of RNA present in the single-stranded repair template. Still, we were able to demonstrate here that when FAAP20 was depleted, even the small extant cohort of RNA-templated repair events was completely ablated and most but not all ssDNA-templated repair events were lost. This suggests that FAAP20 is involved in facilitating HDR when a wide variety of templates are used. This also suggests that the loss of FAAP20 alters the repair dynamics at a DSB in such a way that SSTR becomes even less tolerant of RNA as a repair template. This role of FAAP20 may also relate to its direct interaction with REV1, as Meers et al.^72^ also showed in their study that RNA-templated repair is mediated by Pol ζ in yeast, which contains REV1 as a subunit. Future work will be needed to establish a link between FAAP20 and other potential factors such as Pol ζ in RNA-templated repair. It will also be necessary to determine whether this effect occurs in different cell backgrounds, with longer repair templates, and at DSBs that are not flanked by repetitive sequences on each broken end.

In addition to our finding that FAAP20 has a substantial role in HDR independent of its FA binding partners, we also found that FAAP20 has a significant role in certain HDR events with FANCA. We demonstrate at the biochemical level that FAAP20 drastically stimulates the strand annealing and exchange activities of FANCA by increasing FANCA’s binding affinity to nucleic acid substrates. This stimulation by FAAP20 is similar to our previously reported ability of FANCG to increase the efficiency of FANCA’s biochemical activities, but here we see that FAAP20 and FANCG do not work together to achieve synergistic enhancement of FANCA. This indicates that FAAP20’s stimulation of FANCA has a specific role while FANCG’s stimulation of FANCA’s annealing/exchange is preferred in a different repair context. Yet because FAAP20’s stimulation of FANCA’s biochemical activity is near identical to FANCG, it is still possible that FAAP20 can compensate for FANCG in stimulating FANCA when FANCG function is deficient or impaired.

Like other known HR factors, FAAP20 loss inhibits cell growth in response to DSB damage or PARPi. This is likely not due to the SSA function of FAAP20 because previous work has shown that simultaneous loss of RAD52 and PARP1 has a minimal effect on cell proliferation in a BRCA-proficient cell background^77^. It is more likely that PARPi sensitivity stems from the loss of HR specifically, and/or a canonical FA pathway function.

The strong role for FAAP20 in HR and cell proliferation compared to FANCA elucidates repair mechanisms that can be preferentially utilized by dividing cancer cells. Other studies have shown that FAAP20^−/−^ mice have milder MMC sensitivity and greater amounts of colony formation in normal hematopoietic precursor cells than FANCA^−^^/^^−^ mice^78^. This discrepancy emphasizes the influence of cell type when evaluating the importance of FAAP20’s repair roles and suggests the ability for selective therapeutic targeting of cancer cells over non-cancer cells by modulating FAAP20 activity. It is likely that the strong dependency on FAAP20 in clonogenic survival that we observe is specific to cancer cells that depend on alternative DSB repair subpathways to support HR and replication fork stability.

The identification of FAAP20 as a major HDR factor strengthens the notion that FAAP20 has significant diagnostic and prognostic value in cancer. In fact, a recent study has shown that patient cancers harboring FAAP20 mutations had the lowest median survival when compared with cancers harboring mutations in 22 other known HR genes^79^. Additionally, FAAP20 mutation in cancer causes HDR deficiency which is highly prognostic in itself^80–85^. Despite the significant clinical outcome associated with FAAP20-mutated cancers, genetic screening for FAAP20 patient variants is very uncommon, warranting increased attention and testing of FAAP20’s expression status in aggressive cancers. Not only does FAAP20’s role as an HDR factor implicate its prognostic value in cancer development but may also be predictive in tumorigenesis. While no FA patients have been discovered with a FAAP20 germline mutation so far^20,21^, a patient has been identified with a FANCA mutation that is proficient in FANCI/FANCD2 monoubiquitination but has completely lost the ability to interact with FAAP20^45^. This FANCA variant demonstrates that the interaction of FAAP20 and FANCA is not necessary for canonical FA pathway activation. Interestingly, this patient developed triple negative breast cancer (TNBC) at a young age, which is typically observed in BRCA1 mutation carriers^86,87^. This may indicate that the interaction of FAAP20 with FANCA functions as a tumor suppressor specifically in TNBC, which could involve the SSA function of FAAP20/FANCA that we have observed. Additionally, a recent study has demonstrated that FAAP20 is a strong prognostic risk factor for breast cancer development where high FAAP20 expression level was associated with higher T stage and worse overall prognosis in human breast cancer patients^88^. In this study, however, FANCA did not rank as a prognostic factor in breast cancer which strengthens the importance of FAAP20’s repair roles in cancer cells. The prognostic value of FAAP20 expression in breast cancer progression suggests that FAAP20 protein could be a promising target in breast cancer treatment.

While we have highlighted the importance of FAAP20’s participation in HDR, it is hopeful that future work will reveal the cellular contexts and protein factors that facilitate a dependency on FAAP20’s roles. Together, our work places a focus on FAAP20 in supporting repair of DSBs, which has future applications in understanding cancer growth and therapy resistance.

## Methods

### Protein expression and purification

Purification of human recombinant FANCA and FANCG was performed as previously described^44^. In summary, Human WT FANCA protein was expressed in Hi5 insect cells. After cell lysis with a dounce homogenizer, extracts were run on HiTrap Blue, Mono Q, and Superdex 200 gel filtration columns (GE Healthcare). Human WT FANCG with a 6× N-terminal His tag was also expressed in Hi5 insect cells which were lysed with a dounce homogenizer. Extracts were then run through a 5 ml HiTrap chelating nickel column and Superdex 200 gel filtration columns. Human WT FAAP20 protein with a 6× C-terminal His tag was expressed in SF9 insect cells using the Bac-to-bac plasmid/baculovirus expression system. Cells were harvested 48 h after baculovirus treatment and lysed in nuclear extraction buffer (50 mM Hepes KOH pH 7.9, 10% Sucrose, 1× protease inhibitor cocktail, 500 mM NaCl, 10 mM β-BME). Extracts were pelleted at 16,000 rpm 4 °C for 2 h and supernatant was passed through a 5 ml HiTrap chelating nickel column. Elution from the nickel column was done using a step gradient with increasing concentrations of Nickel B buffer (50 mM Na_2_HPO_4_, 500 mM imidazole, 300 mM NaCl, 10 mM BME, 1× protease), where FAAP20 eluted in 70% Nickel B buffer. The FAAP20-containing fraction was then diluted with Phosphate A buffer (50 mM K_3_PO_4_, 1 mM DTT, 5 mM EDTA, 1× PI, 10% glycerol) to 150 mM NaCl, then applied to MonoQ with the elution peak at 350 mM KCl. Eluted FAAP20 fractions were then applied to a Superdex-200 gel filtration with final elution into Phosphate A buffer, 220 mM KCl.

### Electrophoretic mobility shift assay (EMSA)

EMSA experiments were performed as previously described^51^. To summarize, oligonucleotide substrates were labeled with γ-^32^P-ATP and T4 polynucleotide kinase enzyme (New England Biolabs) according to the manufacturer’s instructions. Indicated recombinant protein and ^32^P-labeled-substrate concentrations were incubated together in 10 μl reaction mixtures that also contained 125 mM Tris pH 7.5, 5 mM EDTA, 6% glycerol, 1 mM DTT, and 100 mM NaCl. Reactions were incubated at RT for 45 min then stopped with 4 μl stop buffer (50% sucrose, 10 mM Tris pH 7.5). Products were run on 4% non-denaturing polyacrylamide gels for 40 min, 100 V in 1× TAE. Shifted bands were then visualized through autoradiography. Double-stranded DNA substrates and splayed arm substrates were prepared by annealing in a 95 °C water bath for 5 min and cooling overnight.

### DNA annealing and strand exchange assays

Biochemical annealing and exchange assays were carried out as previously described^44^. Briefly, indicated concentrations of recombinant protein were added to 10 μl reaction mixtures along with 0.5 nM each oligonucleotide, where one strand only is labeled at the 5’ end with γ-^32^P. Reaction mixtures also contained 25 mM Tris-HCl pH 8, 1 mM EDTA, 110 mM NaCl. Positive control reactions contained 1 nM annealed duplex DNA while negative control reactions contained 0.5 nM each free oligonucleotide, and both reactions contained 10% BSA + PBS as a protein control. The reactions were incubated for 40 min at RT and then stopped by addition of 1 μl 200 mM EDTA, 32% Glycerol, 1% SDS, 0.024% Bromophenol Blue, protease K, and 10 min incubation at room temperature. Products were then resolved on a 6% non-denaturing acrylamide gel at 100 V for 90 min in 1X TBE.

### ChIP-qPCR in DiVA-U2OS cells

DIvA-U2OS cells were kindly provided to us by Dr. Gaelle Legube from the University of Toulouse, France. To perform ChIP-qPCR, DIvA cells were synchronized and released into S phase prior to 4-OHT treatment (2 mM thymidine added, washed out 24 h later, re-added 8 h later, washed out 16 h later). Six hours after the second release, 300 nM 4-OHT was added to half of the cells, where the other half were left untreated. Cells were then crosslinked with formaldehyde and harvested 2 h later. ChIP was then performed using the SimpleChIP Plus Sonication ChIP Kit (Cell Signaling Technology) according to manufacturer’s instructions, using 2 μg anti-FANCA antibody (Bethyl LS-C823145) for each reaction, and 2 μg Normal Rabbit IgG (Cell Signaling 2729) was used for a negative control. IP efficiency was measured as a % of input immunoprecipitated DNA.

### Clonogenic survival assay

MIA PaCa-2 and HeLa cells were seeded at 5 × 10^5^ cells/60 mM dish. U2OS cells were seeded at either 5 × 10^5^ cells/60 mM dish, or 4 × 10^4^ cells/60 mM dish. Each dish was then transfected with either 800 ng/expression plasmid (FANCA-plvx IRES-Neo, FANCG-plvx-IRES-Neo, FAAP20-pcDNA, plvx-IRES-Neo empty vector) or 80 pmol targeting siRNA (FANCA, FANCG, FAAP20 Smartpool-Dharmacon), or scrambled siCtrl (ON-TARGET*plus* Non-targeting Control Pool-Dharmacon). In all, 3 μl Lipofectamine 2000/expression plasmid or 4 μl RNAimax/siRNA target was diluted in Optimem reduced serum-medium (Gibco), and added to indicated nucleic acids, and incubated at RT for 25 min. Transfection complexes were then added to cell dishes overnight, and media was changed 16 h later. Twenty-four hours later, cells were harvested, counted, and seeded into 6-well plates at 500 cells/well. Twenty-four hours after seeding wells, plates were either treated with 5 μM DI03 (Sigma), 10 uM AZD2461 (Sigma), 0.5 Gy IR, or left untreated. Plated cells grew for 14 days before staining with crystal violet.

### DSB reporter assays

All GFP-U2OS reporter cell lines used in this study (HR, NHEJ, Alt-EJ, SSA, Δ7 RMR- DK71G) were kindly shared by Dr. Jeremy Stark, City of Hope. For all reporters, cells were seeded at 5 × 10^5^ cells/60 mM dish. Cells were then transfected with indicated siRNAs (80 pmol + 4 μl RNAimax) or expression plasmids (800 ng + 4 μl Lipofectamine 2000). Two days later, DSB reporter cells (HR, NHEJ, Alt-EJ, or SSA) were transfected with 800 ng of either pBaSCEI-CMV-mCherry and 6 μl Lipofectamine 2000. In the case of Δ7 RMR cells, cotransfection of 800 ng each pRP(CRISPR-hCas9-mcherry-U6) with 3’ and 5’ targeting gRNA sequences and 2.5 pmol oligonucleotide repair templates was performed using 6 μl Lipofectamine 2000. Two days after nuclease transfection, cells were harvested and fixed with 2% formaldehyde and were analyzed by flow cytometry with an LSR-Fortessa-HTS. GFP-positive cells were counted as a percentage of the mCherry-positive cell population. Results are reported as a percentage of the siCtrl or empty vector-transfected control. mCherry constructs were added to pBaSCEI and CRISPR plasmids by VectorBuilder Inc.

### Coimmunoprecipitation

co-IP was performed in HEK293t whole-cell extracts 48 h after either FAAP20 overexpression or vector control expression, using 500 μg protein/sample. Extracts were treated with Micrococcal Nuclease (NEB) for 1 h prior to addition of antibody. 2% Input was reserved for Western blotting. 2 μg each antibody was added to extracts (RAD51, BioAcademia; Rb IgG, Sigma) which were rotated at 4 °C overnight. The next morning, 10 μl Protein A magnetic beads (Cell Signaling #73778 S) were added and samples were rotated at 4 °C for 2 h. Beads were washed 5× using a magnetic stand and 1× wash buffer (Cell Signaling #9803). Bound protein was eluted by boiling for 5 min. Immunoprecipitated protein samples and 2% Input samples were then analyzed by Western blotting using RAD51 (BioAcademia), FAAP20 (Sigma), and 53BP1 (Sigma) antibodies.

### Cell culture

U2OS, MIA PaCa-2, and HeLa cells were cultured in Dulbecco’s modified Eagle’s medium (DMEM, high glucose, Sigma) with 10% FBS and penicillin–streptomycin/antimycotic antibiotics (Thermo Fisher Scientific), in standard cell culture conditions of 5% CO_2_ with humidified atmosphere at 37 °C. These cells were obtained from ATCC where their identity was validated. Despite categorization as a commonly misidentified cell line, we have ensured the identity of HeLa cells which serve as an excellent model for studying effects on cancer cell biology in female reproductive tissue. DIvA-U2OS, 282, 283, 280, and 279-U2OS cells were supplemented with 1 μg/ml puromycin. For siRNA transfections, Dharmacon SMARTpool containing (4) individual targeting siRNAs were used for each gene knockdown. This includes FANCA, FAAP20, FANCG, FANCD2, and BRCA2 knockdowns as well as the ON-TARGETplus Non-targeting Control Pool for siCtrl. Cells were transfected with siRNA for 16 h followed by washing out with fresh media, and protein knockdown was validated by western blot 48 h after initial transfection.

### Chromatin fractionation

Chromatin fractionation was performed as reported previously^89^. Briefly, 48 h after siRNA transfection and 6 h after irradiation, U2OS cells were harvested and incubated in hypotonic buffer on ice for 15 min (10 mM HEPES, pH 7, 50 mM NaCl, 0.3 M sucrose, and 0.5% Triton X-100, 1× PI cocktail). Cells were then centrifuged for 5 min at 1500 × *g*, and the supernatant contained the cytosolic fraction. Then cells were incubated on ice with nuclear extraction buffer (10 mM HEPES, pH 7, 200 mM NaCl, 1 mM EDTA, 0.5% NP-40, and 1× PI cocktail), then centrifuged for 2 min at 13,000 rpm. Supernatant was then removed and stored, then cells were treated with lysis butter, sonicated at 15% amplitude, then centrifuged for 1 min at 13,000 rpm. Supernatants were saved as chromatin-bound extracts, which were later analyzed by immunoblotting.

### Immunoblot analysis

Cells were harvested and resuspended in protein extraction buffer (50 mM Na_3_PO_4_, 300 mM NaCl, 0.5% NP-40, 1 mM DTT, 1× PI cocktail). Cells were then sonicated, centrifuged at 4 °C, and supernatants were analyzed for protein concentration using the Bradford method. 20–80 μg protein were loaded in each well of a polyacrylamide gel after denaturing 5 min at 95 °C. Gels were run 1–1.5 h at 150 V then transferred to nitrocellulose membranes, blocked for 1 h with 5% milk, then probed with the following antibodies: FANCA (Bethyl-1:1000), FAAP20 (Sigma, Weidong Wang-1:250), FANCG (Santa Cruz-1:50), Actin (Santa Cruz-1:2000), H2B (Cell Signaling Technologies-1:1000), BRCA2 (Sigma, OHSU-1:500), RAD51 (BioAcademia-1:500), HSP90 (Santa Cruz-1:2000), FANCD2 (Proteintech-1:1000). Membranes were then washed 2× with TBST, probed with secondary antibody for 1 h (anti-Mouse HRP, anti-Rabbit HRP, Sigma), washed 3× with TBST, then developed using peroxide treatment and autoradiography.

### Immunofluorescent staining and analysis

In all, 1 × 10^6^ U2OS cells were seeded into 100 mM dishes and transfected with one of the indicated siRNAs (140 pmol siRNA + 8 μl RNAimax). 48 h later, cells were irradiated with 2 Gy IR using a cesium irradiator. Cells were harvested 6 hr after irradiation and fixed with 4% paraformaldehyde for 10 min. Cells were then washed 2× with PBS and ~50,000–100,000 cells were dropped onto poly-L-lysine-coated slides where they were further attached using 4% formaldehyde. Cells were then blocked for 30 min (1% BSA, 0.1% TritonX100, 5% horse serum in PBS), then treated with primary antibodies diluted in blocking solution for 1 h (γ-H2AX: 1:2000 Sigma H2A.X Ser139, clone JBW301; 53BP1: 1:2000 Sigma; BRCA1: 1:500 Sigma 07-434; RAD51: 1:1000 Bio-Academia 70-001). Then, cells were washed 2× with PBS and treated with either Alexa-488 anti-rabbit or Alexa-594 anti-mouse secondary antibodies diluted in blocking buffer at RT for 30 min (1:1000, ThermoFisher). Slides were then washed 3× with PBS and treated with DAPI for 5 min. Slides were then washed 3× more with Millipore water, dried, sealed with coverslips and analyzed the following day using a DMI6000B microscope with LASX software.

### Cell cycle analysis

U2OS cells were treated with the same conditions used for IF staining experiments (siRNA transfected, IR-treated, harvested 6 h after irradiation). Cells were then washed with PBS and fixed with ice cold 70% Ethanol for 30 min on ice. Cells were then washed 2× with PBS then treated with 5 μg RNAse. Cells were then resuspended in PBS with 25 μl propidium iodide (PI) solution (0.5 mg/ml). 30 min later, cells were analyzed by flow cytometry.

### Statistics and reproducibility

All Student’s two-tailed *t* tests were performed using GraphPad Prism 8.4.3 for Windows. The sample size used for each analysis is listed in the figure legend for each corresponding data set. All measurements used in statistical analyses were taken from distinct samples.

### Reporting summary

Further information on research design is available in the Nature Portfolio Reporting Summary linked to this article.

### Supplementary information

Peer Review File

Supplementary Information

Description of Additional Supplementary Files

Supplementary Data

Reporting Summary

## Data Availability

Data are available upon request. Data points for all graphs are available in Supplementary Data. All uncropped images are also provided in Supplementary Information as Supplementary Fig. 8. Oligonucleotide sequences are also available in Supplementary Information (Supplementary Table 1).
