# Supplementary material for: Fanconi anemia associated protein 20 (FAAP20) plays an essential role in homology-directed repair of DNA double-strand breaks
Source: Commun Biol. 2023 Aug 24;6:873. doi: 10.1038/s42003-023-05252-9 (PMC10449828; doi:10.1038/s42003-023-05252-9)
Supplement: Supplementary file 2 — Supplementary Information [file 42003_2023_5252_MOESM2_ESM.pdf]

Supplementary Information for

Fanconi anemia associated protein 20 (FAAP20) plays an essential role in homology-directed repair of DNA double-strand breaks

Anna Palovcak[1], Fenghua Yuan[1], Ramiro Verdun[2], Liang Luo[1], Yanbin Zhang[1]

[1]Department of Biochemistry & Molecular Biology, [2]Department of Medicine, University of Miami Miller School of Medicine, Miami, FL, 33136, USA.

This file includes:

Supplementary Figures 1-8

Supplementary Table 1

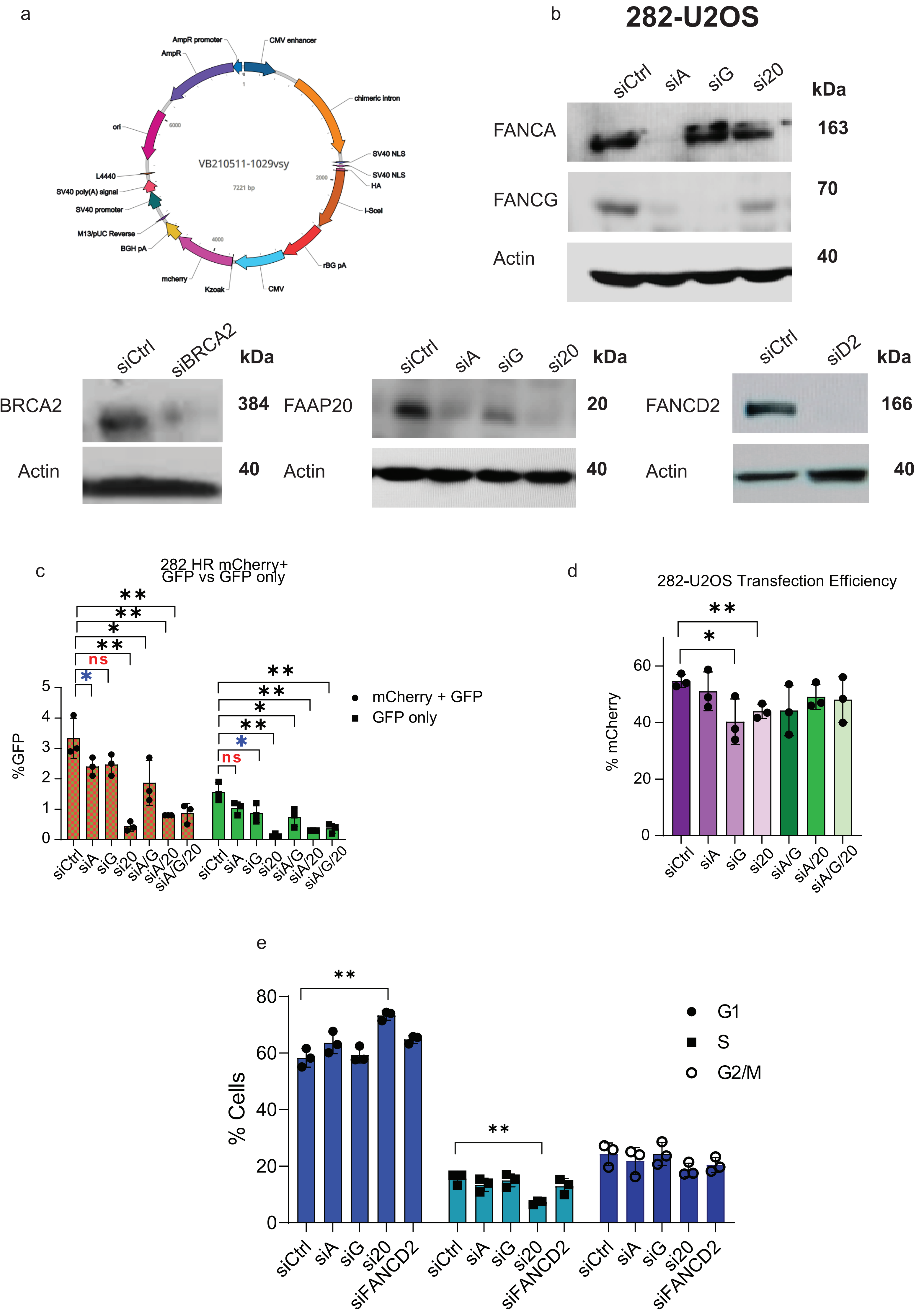
a
AmpR promoter
CMV enhancer
AmpR
chimeric intron
ori
6000
1
VB210511-1029vsy
7221 bp
SV40 NLS
SV40 NLS
HA
2000
L4440
SV40 poly(A) signal
SV40 promoter
I-SceI
M13/pUC Reverse
BGH pA
4000
rBG pA
mcherry
Kzoak
CMV
b
282-U2OS
siCtrl
siA
siG
si20
kDa
FANCA
163
FANCG
70
Actin
40
siCtrl
siBRCA2
kDa
BRCA2
384
Actin
40
siCtrl
siA
siG
si20
kDa
FAAP20
20
Actin
40
siCtrl
siD2
kDa
FANCD2
166
Actin
40
c
282 HR mCherry+
GFP vs GFP only
%GFP
ns
mCherry + GFP
GFP only
siCtrl
siA
siG
si20
siA/G
siA/20
siA/G/20
d
282-U2OS Transfection Efficiency
% mCherry
siCtrl
siA
siG
si20
siA/G
siA/20
siA/G/20
e
% Cells
G1
S
G2/M
siCtrl
siA
siG
si20
siFANCD2

Supplementary Figure 1. Expanded HR and NHEJ DSB repair outcomes

a, Plasmid map of I-SceI expression plasmid containing a constitutively-active mCherry expression cassette. **b**, Western blots showing knockdown of indicated proteins in 282-U2OS cells. Actin is used as a loading control. **c**, measure of GFP+ cells in 282-U2OS with each knockdown condition relative to mCherry-expressing cells (first data set) compared with GFP+ only as a percentage of the entire measured cell population (second data set). Red lettering indicates FANCG knockdown condition, that loses its effect on HR when transfection efficiency is normalized using mCherry-based gating. Blue lettering indicates siFANCA cells that do not show a significant effect on HR until transfection efficiency is normalized using mCherry-based gating. **d**, Measure of mCherry signal as a % of the total cell population in each knockdown condition. This is performed in 282-U2Os cells and corresponds to transfection efficiency. **e**, Measure of cell cycle distribution in 282-U2OS cells with the indicated siRNA knockdown conditions using PI staining For **c**-**e**, bars show mean with error bars as SD. Two-tailed student's t-test were used for statistical analysis where *P<0.05, **P<0.01; n=3

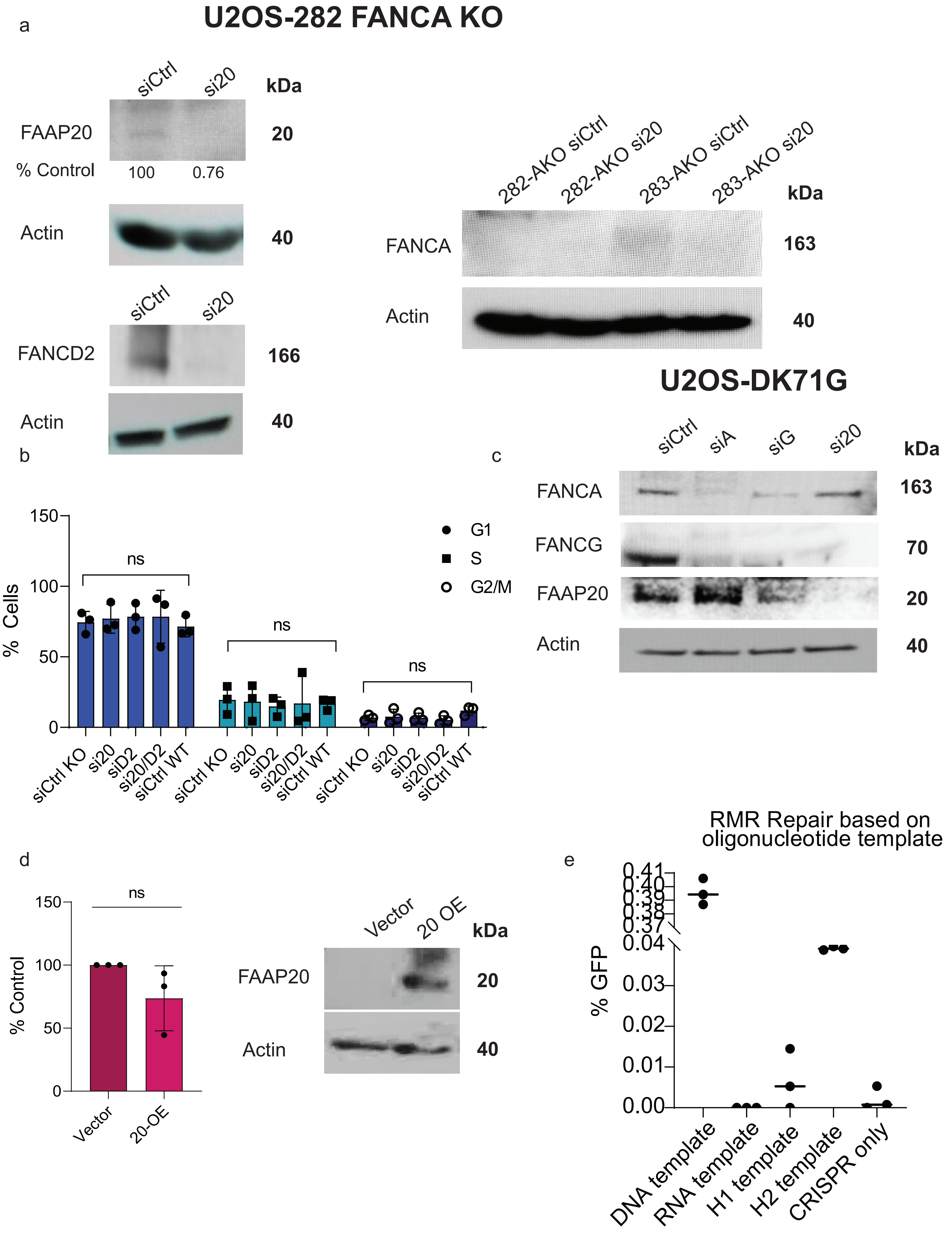

a
U2OS-282 FANCA KO
siCtrl
si20
kDa
FAAP20
20
% Control
100
0.76
Actin
40
siCtrl
si20
FANCD2
166
Actin
40
282-AKO siCtrl
282-AKO si20
283-AKO siCtrl
283-AKO si20
kDa
FANCA
163
Actin
40
U2OS-DK71G
siCtrl
siA
siG
si20
kDa
FANCA
163
FANCG
70
FAAP20
20
Actin
40
b
c
% Cells
150
100
50
0
ns
ns
ns
G1
S
G2/M
siCtrl KO
si20
siD2
si20/D2
siCtrl WT
siCtrl KO
si20
siD2
si20/D2
siCtrl WT
siCtrl KO
si20
siD2
si20/D2
siCtrl WT
d
% Control
150
100
50
0
ns
Vector
20-OE
Vector
20 OE
kDa
FAAP20
20
Actin
40
e
RMR Repair based on oligonucleotide template
% GFP
0.41
0.40
0.39
0.38
0.37
0.04
0.03
0.02
0.01
0.00
DNA template
RNA template
H1 template
H2 template
CRISPR only

Supplementary Figure 2. U2OS 282 FANCA KO and DK71G characterization

a, Western blots showing siRNA knockdown of indicated proteins in 282-U2OS cells with FANCA KO generated by CRISPR-Cas9. Densitometry analysis was used to quantify and compare FAAP20 protein levels in the siCtrl vs siFAAP20-treated 282 FANCA KO cells. Band quantitation is reported as a % of siCtrl. Actin is used as a loading control. **b**, Analysis of cell cycle distribution in 282-U2OS with FANCA KO in addition to the indicated protein knockdown conditions using PI staining. Two-tailed student's t-test was used for statistical analysis where ns=not significant. Bars are shown at the mean value; n=3 **c**, Western blots showing siRNA knockdown of indicated proteins in U2OS-DK71G. Actin is used as a loading control. **d**, Measure of GFP+ events in DK71G indicating successful RMR repair, shown as % of siCtrl with the indicated protein overexpression conditions. Western blots confirming FAAP20 overexpression is also shown. **e**, measure of repair events observed with each oligonucleotide template, showing how repair efficiency drops precipitously with increasing amounts of RNA content in the repair template. n=3

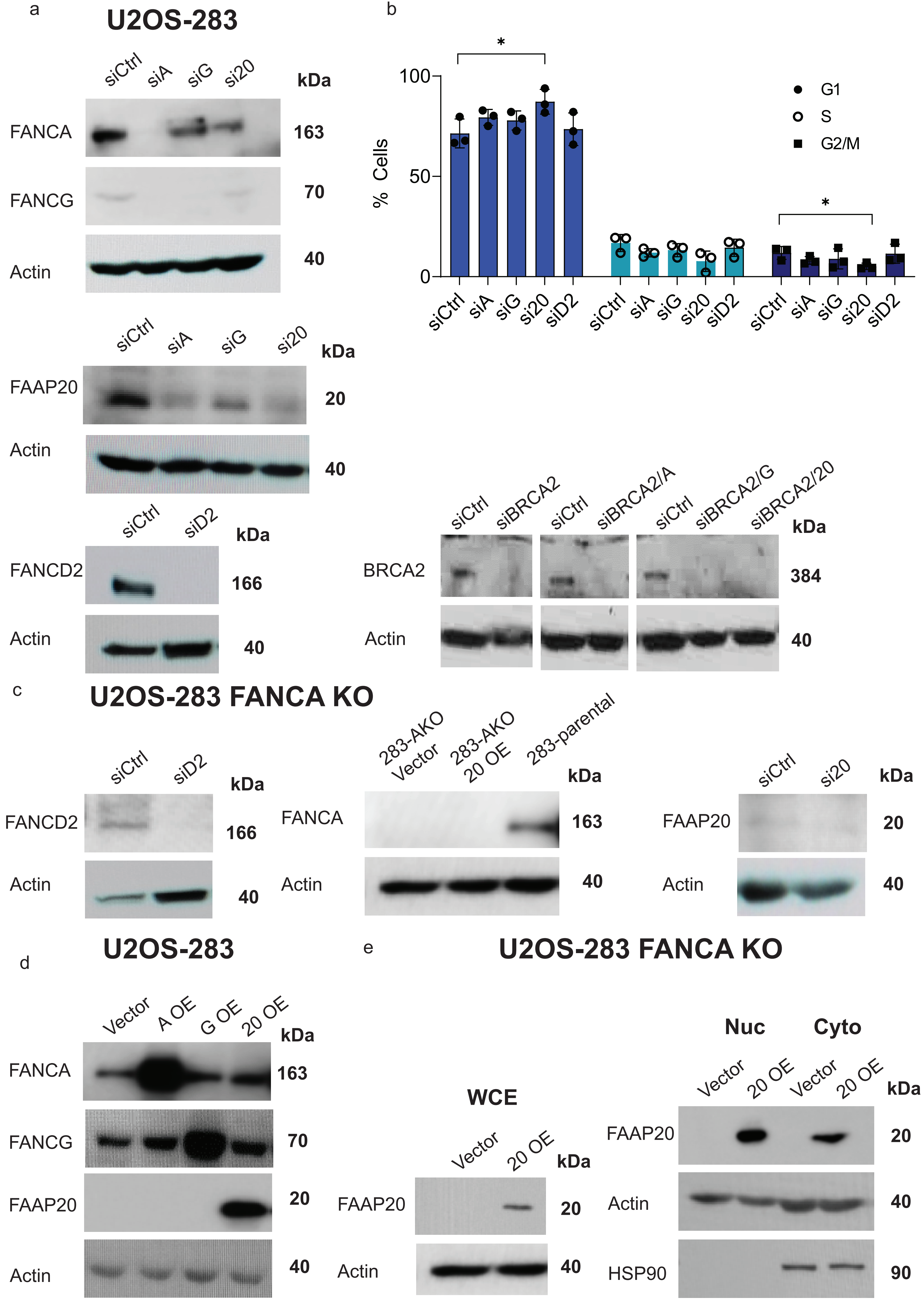
a
U2OS-283
siCtrl
siA
siG
si20
kDa
FANCA
163
FANCG
70
Actin
40
b
100
50
0
% Cells
*
G1
S
G2/M
siCtrl
siA
siG
si20
siD2
FAAP20
20
FANCD2
166
siBRCA2
siBRCA2/A
siBRCA2/G
siBRCA2/20
BRCA2
384
c
U2OS-283 FANCA KO
283-AKO Vector
283-AKO 20 OE
283-parental
d
U2OS-283
Vector
A OE
G OE
20 OE
e
U2OS-283 FANCA KO
WCE
Nuc
Cyto
HSP90
90

Supplementary Figure 3. U2OS 283 WT and FANCA KO confirmation

a, Western blots showing knockdown of indicated proteins in 283-U2OS cells. Actin is shown as a loading control. **b**, analysis of cell cycle distribution in 283-U2OS cells with the indicated knockdown conditions, using PI staining. Bars indicate mean values. Statistical analysis was done using two-tailed Student's t-test where *P<0.05 and error bars show SD, n=3. **c**, Western blots showing knockdown of indicated proteins in 283 FANCA KO cells. Actin is shown as a loading control. **d**, Western blots showing protein levels in 283 cells with indicated proteins overexpressed. Actin is shown as a loading control. **e**, Western blots showing indicated protein knockdown and overexpression conditions in whole cell extracts, as well as nuclear and cytosolic fractions. For subcellular fractionation, HSP90 (cytosolic protein) was used as a fractionation control while Actin was used as a loading control.

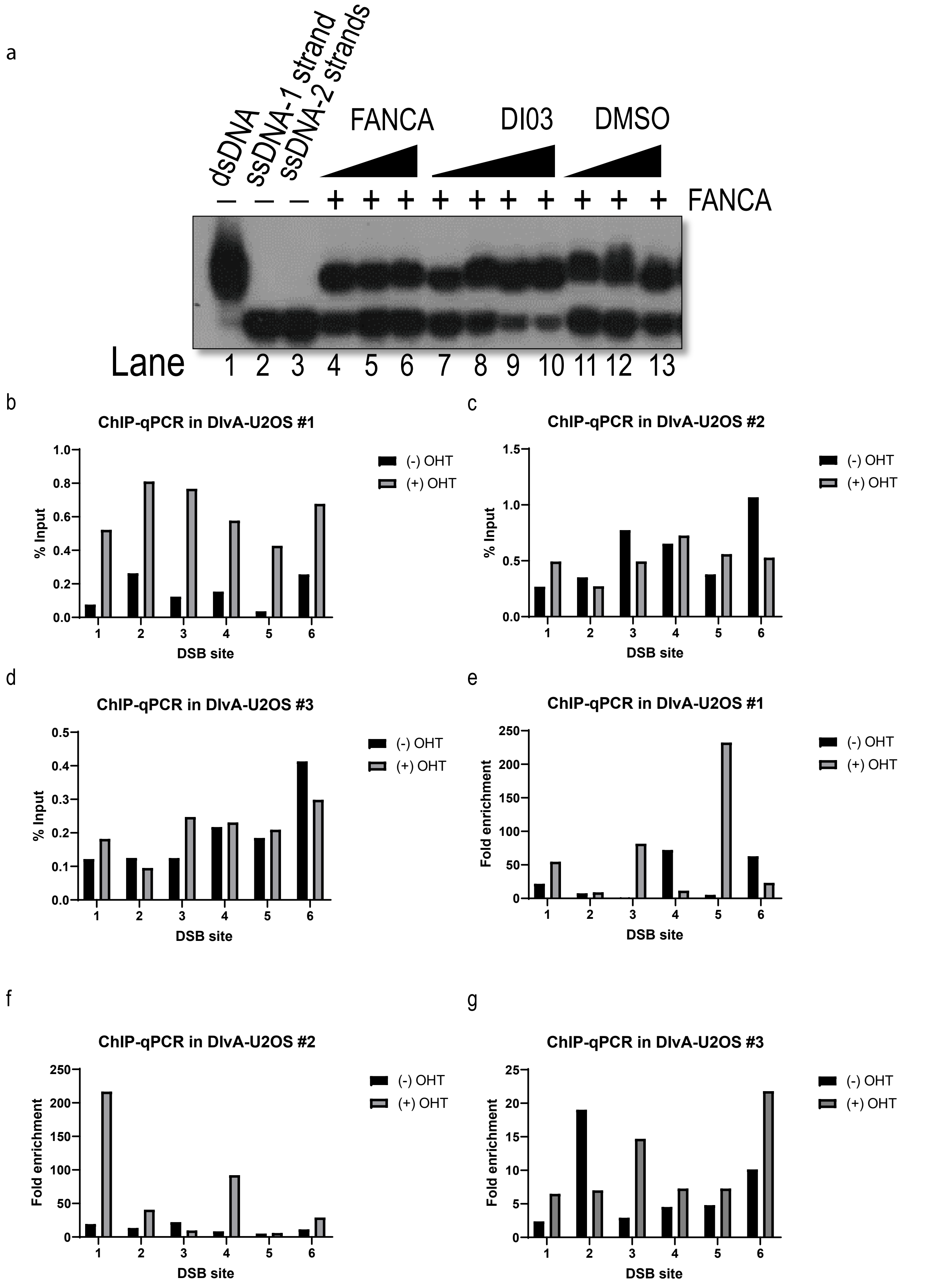

a
dsDNA
ssDNA-1 strand
ssDNA-2 strands
FANCA
DI03
DMSO
− − − + + + + + + + + + + FANCA
Lane 1 2 3 4 5 6 7 8 9 10 11 12 13
b
ChIP-qPCR in DIvA-U2OS #1
% Input
DSB site
(-) OHT
(+) OHT
c
ChIP-qPCR in DIvA-U2OS #2
% Input
DSB site
(-) OHT
(+) OHT
d
ChIP-qPCR in DIvA-U2OS #3
% Input
DSB site
(-) OHT
(+) OHT
e
ChIP-qPCR in DIvA-U2OS #1
Fold enrichment
DSB site
(-) OHT
(+) OHT
f
ChIP-qPCR in DIvA-U2OS #2
Fold enrichment
DSB site
(-) OHT
(+) OHT
g
ChIP-qPCR in DIvA-U2OS #3
Fold enrichment
DSB site
(-) OHT
(+) OHT

Supplementary Figure 4. Biochemical nature of FANCA and FAAP20's nucleic acid processing functions

a, single-strand annealing assay with FANCA protein and DI03 to test for any nonspecific inhibition of FANCA's biochemical activity. Lane 1: positive pre-annealed duplex control, lane 2: ssDNA only, lane 3: No protein control with both oligonucleotide strands, lanes 4-7: 30, 40, 50, 60 nM FANCA without drug or vehicle to show proficient annealing by FANCA, lanes 8-10: fixed concentration of FANCA (60 nM), with increasing concentrations of DI03 (37.5, 75, 150 μM) spanning the concentration used for cell-based assays, lanes 11-13: volume-matched DMSO control. n=1 **b-d**, each of 3 individual repeats of ChIP-qPCR experiments in DIvA-U2OS cells using a FANCA specific antibody and calculating qPCR results using the "% Input" method. Each graph is n=1. **e-g**, using the same experimental qPCR data from the first three figures (a-c) but calculations were done using the "Fold enrichment method." For each graph, n=1

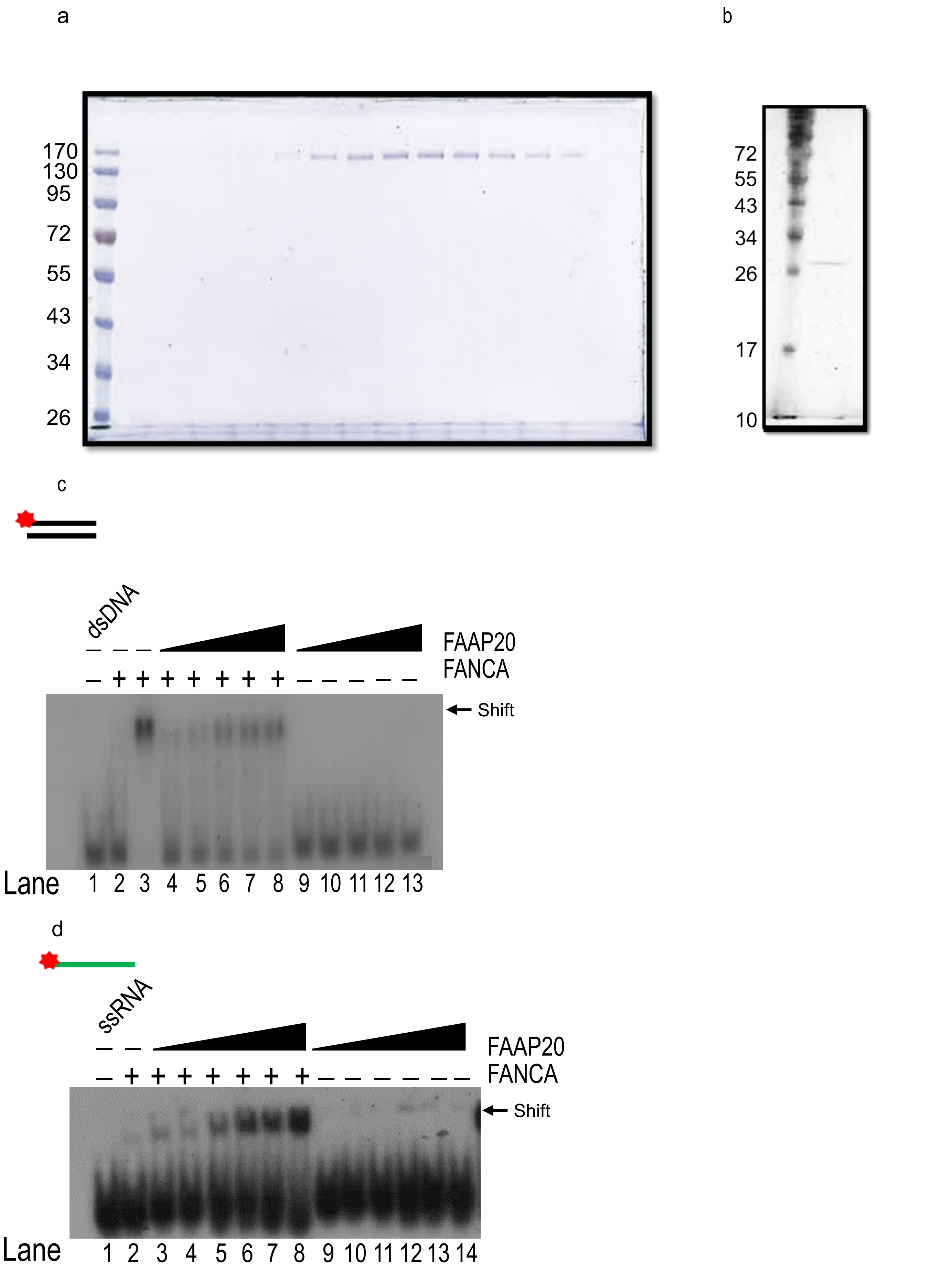

a
170
130
95
72
55
43
34
26
b
72
55
43
34
26
17
10
c
dsDNA
FAAP20
FANCA
– + + + + + + + – – – – –
Shift
Lane 1 2 3 4 5 6 7 8 9 10 11 12 13
d
ssRNA
FAAP20
FANCA
– + + + + + + + – – – – – –
Shift
Lane 1 2 3 4 5 6 7 8 9 10 11 12 13 14

Supplementary Figure 5. FANCA binds to damaged chromatin

a, Coomasie staining of pure, recombinant human native FANCA protein. **b**, Silver staining of pure, recombinant human FAAP20 protein with a C-terminal 6xHis tag. **c**, dsDNA EMSA of recombinant FAAP20 protein titrated against fixed, suboptimal FANCA protein or alone and 1 nM substrate. Lane 1: No protein (PBS/10%BSA); lane 2: suboptimal FANCA alone: 10 nM; lane 3: positive control of high concentration FANCA: 100 nM; lanes 4-8: FAAP20: 15-75 nM in 15 nM increments, and FANCA: 10 nM; lanes 9-13: FAAP20 only: 15-75 nM. n=1. **d**, ssRNA EMSA of recombinant FAAP20 protein titrated against fixed, suboptimal FANCA protein or alone and 1 nM substrate. Lane 1: No protein (PBS/10%BSA); lane 2: suboptimal FANCA alone: 10 nM; lanes 3-8: FAAP20: 1-25 nM (1, 5, 10, 15, 20, 25 nM), and FANCA: 10 nM; lanes 9-14: FAAP20 only: 1-25 nM. n=1

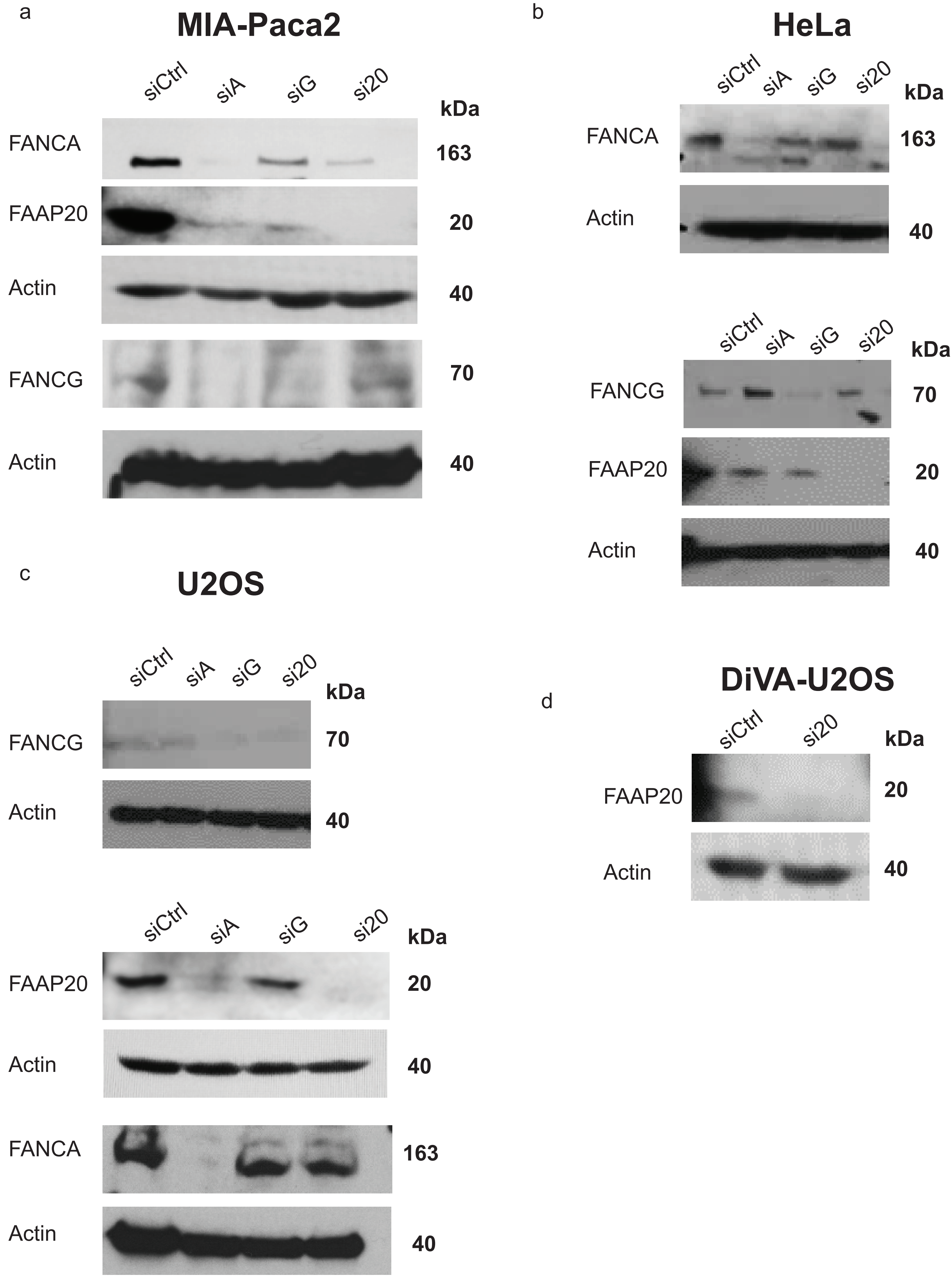
a
MIA-Paca2
siCtrl
siA
siG
si20
kDa
FANCA
163
FAAP20
20
Actin
40
FANCG
70
Actin
40
b
HeLa
siCtrl
siA
siG
si20
kDa
FANCA
163
Actin
40
siCtrl
siA
siG
si20
kDa
FANCG
70
FAAP20
20
Actin
40
c
U2OS
siCtrl
siA
siG
si20
kDa
FANCG
70
Actin
40
siCtrl
siA
siG
si20
kDa
FAAP20
20
Actin
40
FANCA
163
Actin
40
d
DiVA-U2OS
siCtrl
si20
kDa
FAAP20
20
Actin
40

Supplementary Figure 6. Protein expression and stability in multiple cell lines

a, Western blots in MIA PaCa-2 cells showing the indicated protein knockdown with siRNA. **b**, Western blots in HeLa cells showing the indicated protein knockdown with siRNA **c**, Western blots in U2OS cells showing the indicated protein knockdown with siRNA. **d**, Western blots in DiVA-U2OS cells showing FAAP20 protein KD compared to siCtrl. For all westerns in this figure, Actin is used as a loading control.

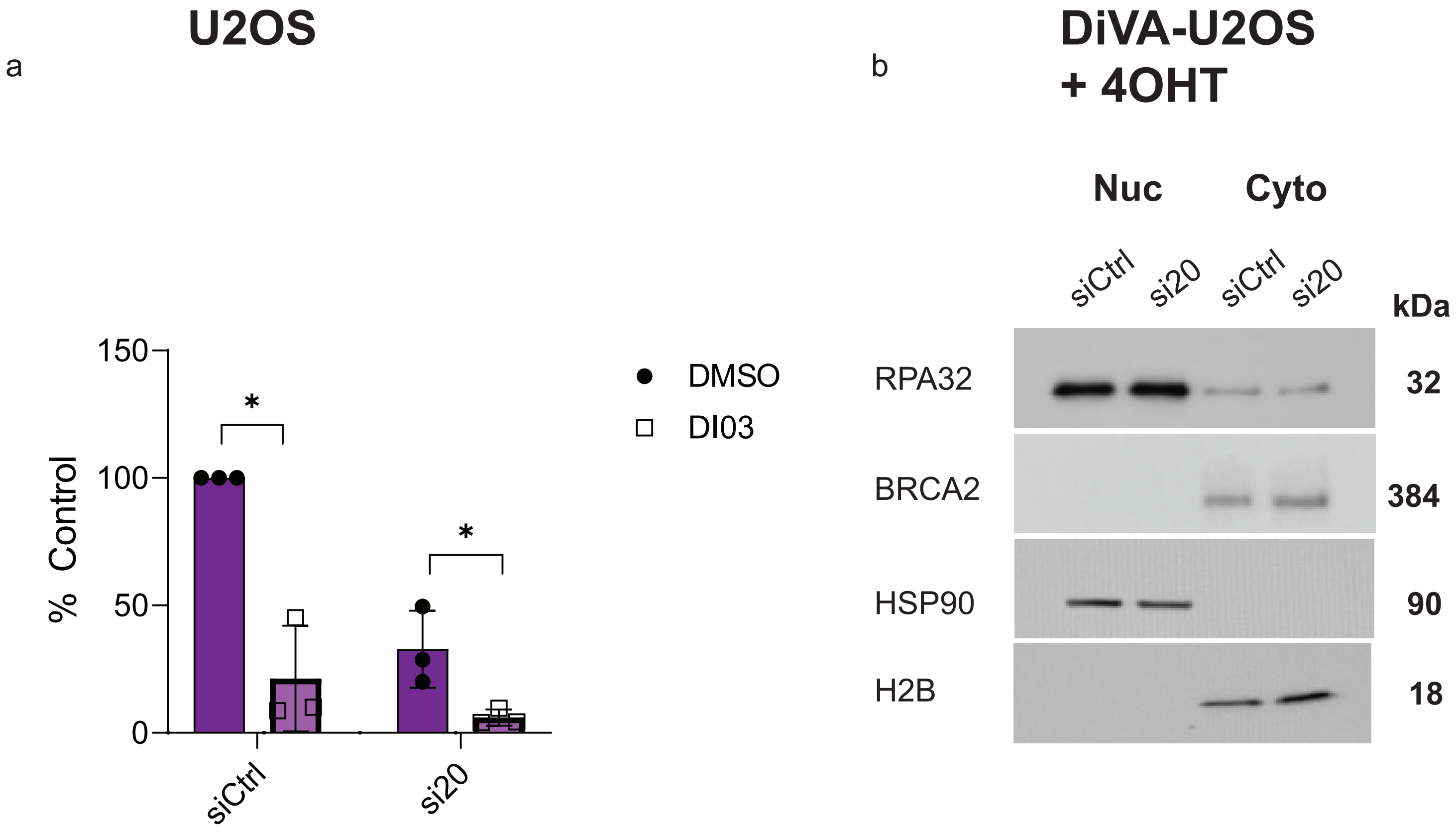
U2OS
a
150
100
50
0
% Control
*
*
siCtrl
si20
DMSO
DI03
DiVA-U2OS
+ 4OHT
b
Nuc
Cyto
siCtrl
si20
siCtrl
si20
kDa
RPA32
32
BRCA2
384
HSP90
90
H2B
18

Supplementary Figure 7. DSB repair implications of FAAP20 loss

a, Cell proliferation assay in U2OS cells after indicated KD and treatment with either DI03 (RAD52 inhibitor) or DMSO control. Results are reported as % DMSO siCtrl, *P<0.05, n=3. **b**, Western blots in DiVA-U2OS cells showing indicated proteins in the nuclear and cytosolic fractions after FAAP20 knockdowns. DiVA-U2OS cells were treated with 4-OHT 3 hours before harvesting and preparing fractionated extracts. HSP90 is used as a cytosolic fractionation/loading control while H2B is used as a nuclear fractionation/loading control.

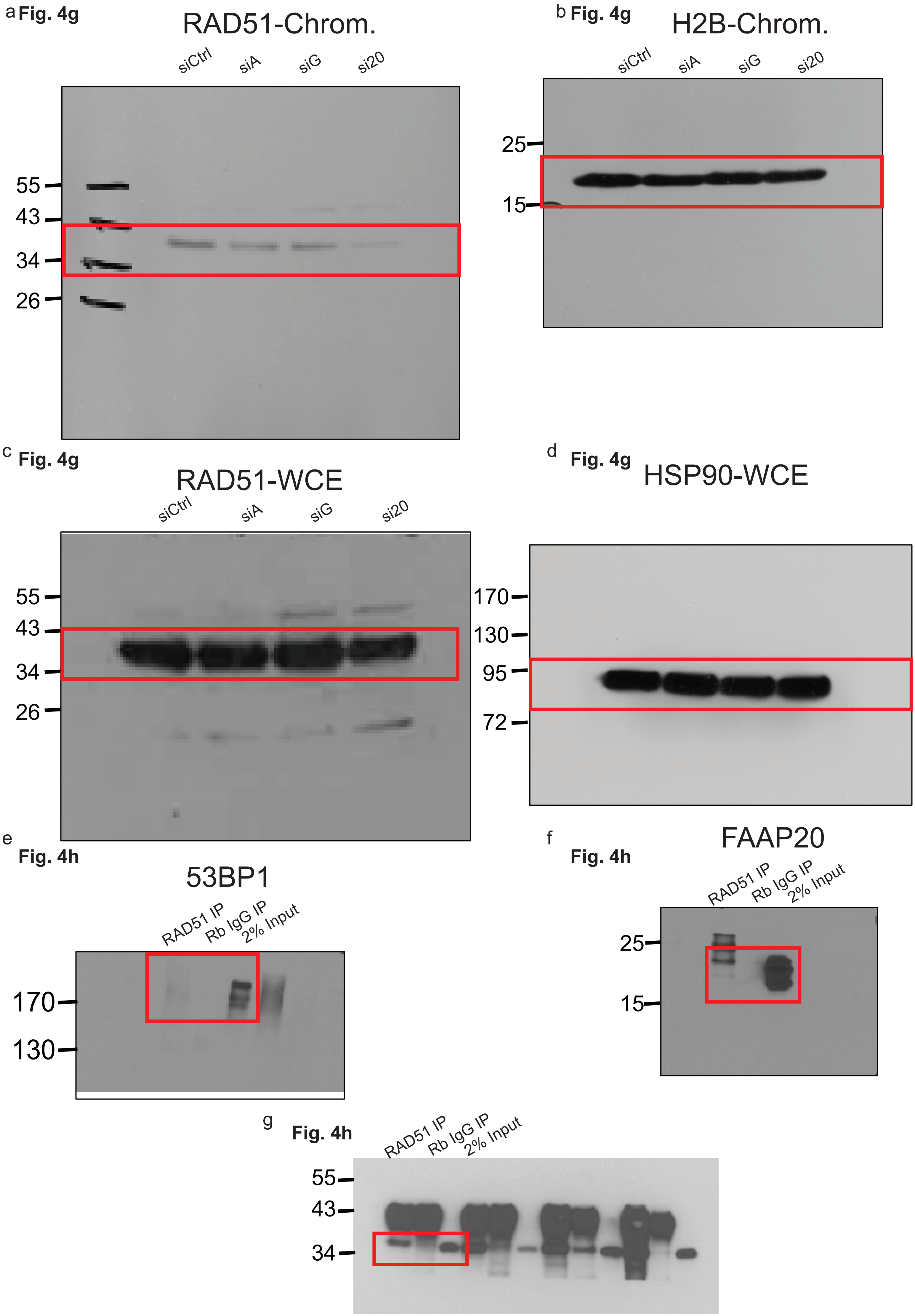

a Fig. 4g
RAD51-Chrom.
siCtrl
siA
siG
si20
55
43
34
26
b Fig. 4g
H2B-Chrom.
siCtrl
siA
siG
si20
25
15
c Fig. 4g
RAD51-WCE
siCtrl
siA
siG
si20
55
43
34
26
d Fig. 4g
HSP90-WCE
170
130
95
72
e Fig. 4h
53BP1
RAD51 IP
Rb IgG IP
2% Input
170
130
f Fig. 4h
FAAP20
RAD51 IP
Rb IgG IP
2% Input
25
15
g Fig. 4h
RAD51 IP
Rb IgG IP
2% Input
55
43
34

282-U2OS

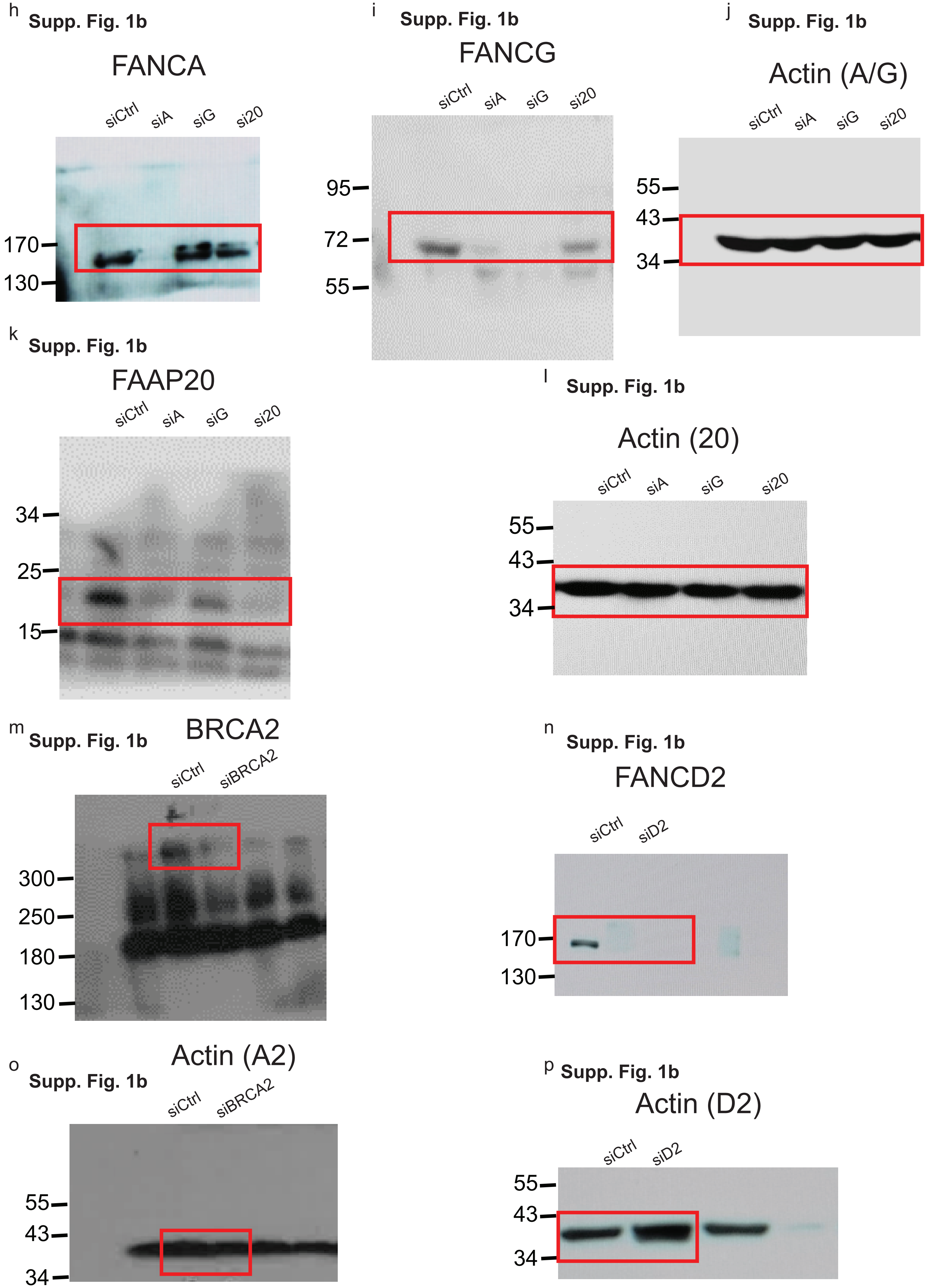

h Supp. Fig. 1b
FANCA
siCtrl siA siG si20
170
130
i Supp. Fig. 1b
FANCG
siCtrl siA siG si20
95
72
55
j Supp. Fig. 1b
Actin (A/G)
siCtrl siA siG si20
55
43
34
k Supp. Fig. 1b
FAAP20
siCtrl siA siG si20
34
25
15
l Supp. Fig. 1b
Actin (20)
siCtrl siA siG si20
55
43
34
m Supp. Fig. 1b
BRCA2
siCtrl siBRCA2
300
250
180
130
n Supp. Fig. 1b
FANCD2
siCtrl siD2
170
130
o Supp. Fig. 1b
Actin (A2)
siCtrl siBRCA2
55
43
34
p Supp. Fig. 1b
Actin (D2)
siCtrl siD2
55
43
34

282-U2OS A KO

q **Supp. Fig. 2a**

FAAP20

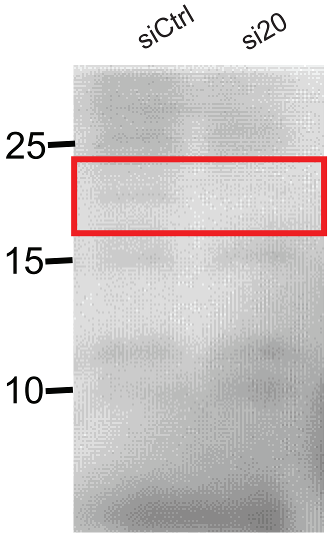

r **Supp. Fig. 2a**

Actin (20)

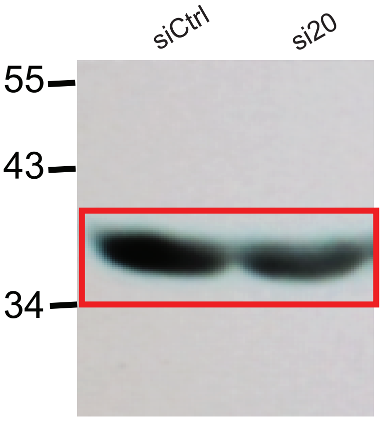

s **Supp. Fig. 2a**

FANCD2

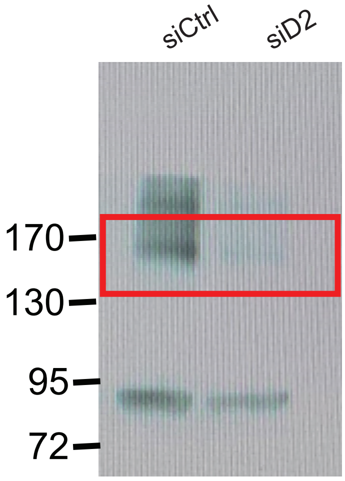

t **Supp. Fig. 2a**

Actin (D2)

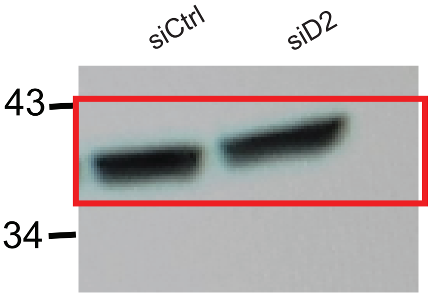

u **Supp. Fig. 2a**

FANCA

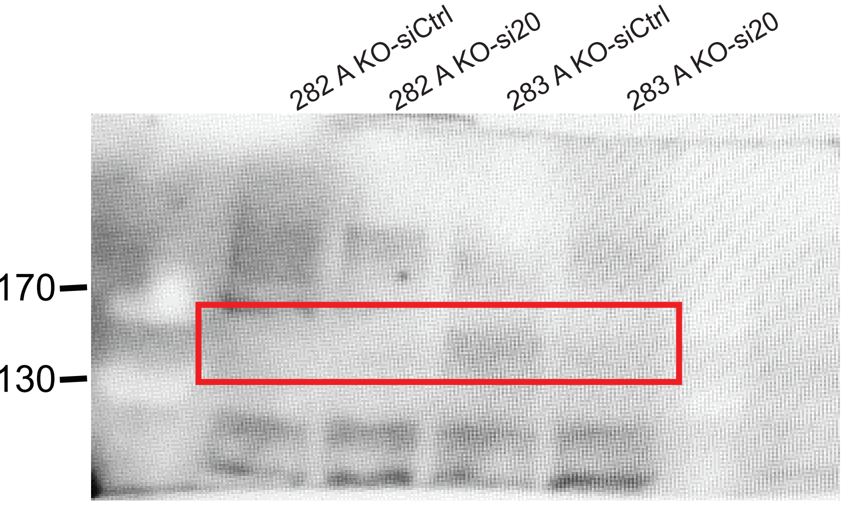

v **Supp. Fig. 2a**

Actin (A)

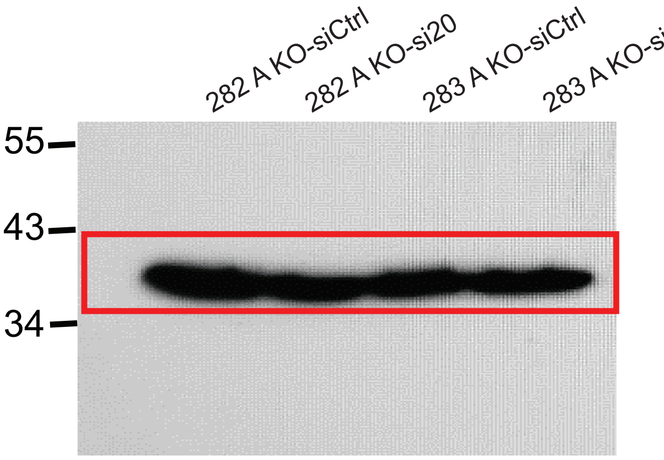

U2OS-DK71G

w **Supp. Fig. 2c**

FANCA

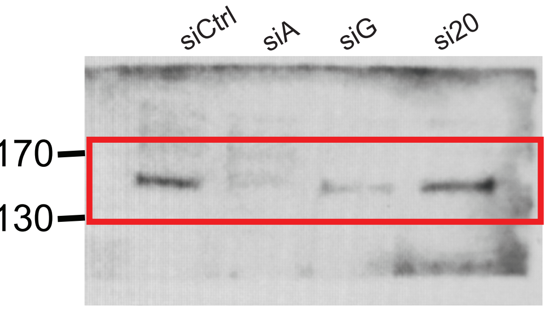

x **Supp. Fig. 2c**

FANCG

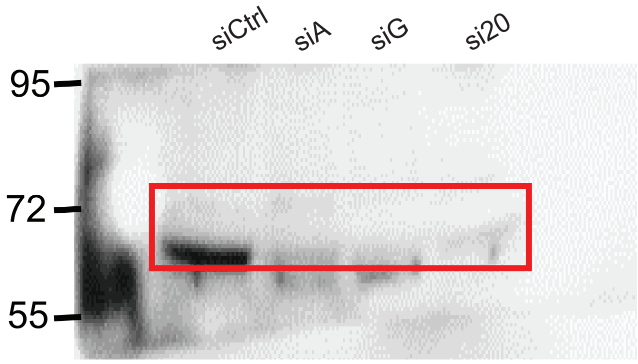

y **Supp. Fig. 2c**

FAAP20

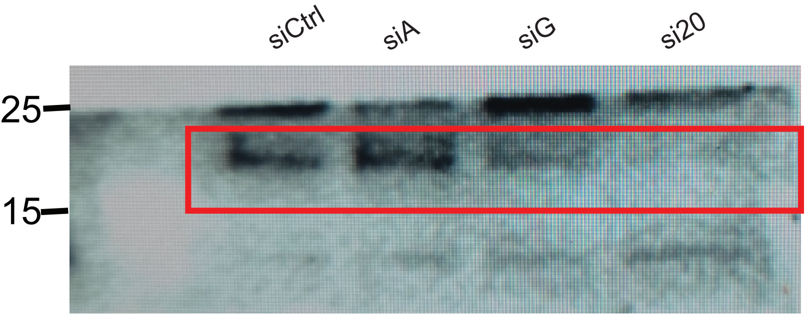

z **Supp. Fig. 2c**

Actin (A/G/20)

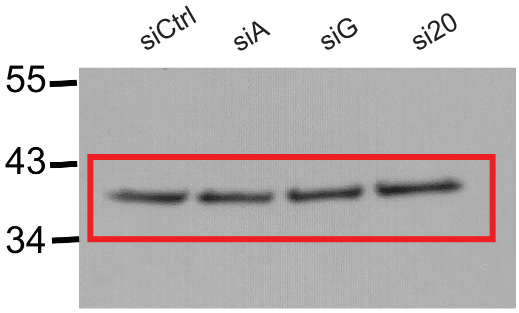

aa **Supp. Fig. 2d**

FAAP20

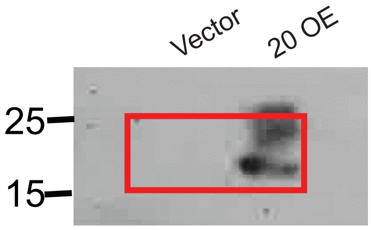

bb **Supp. Fig. 2d**

Actin (20)

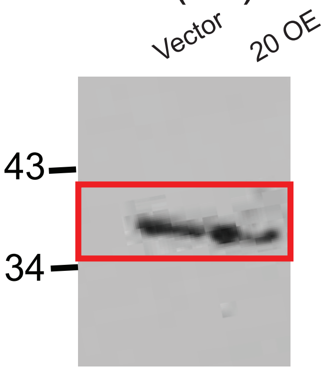

U2OS-283

cc **Supp. Fig. 3a**

FANCA

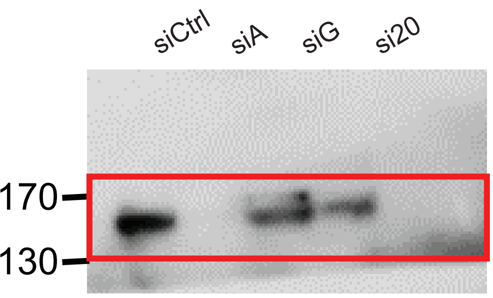

dd **Supp. Fig. 3a**

FANCG

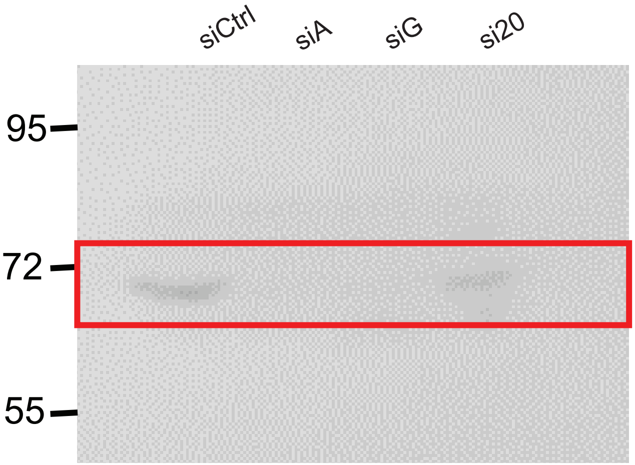

ee **Supp. Fig. 3a**

FAAP20

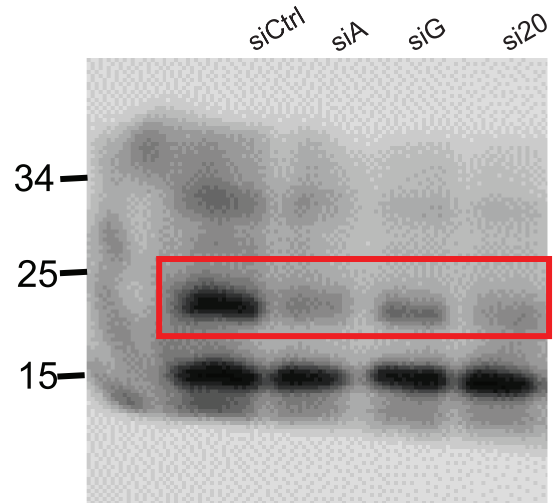

ff **Supp. Fig. 3a**

Actin (A/G/20)

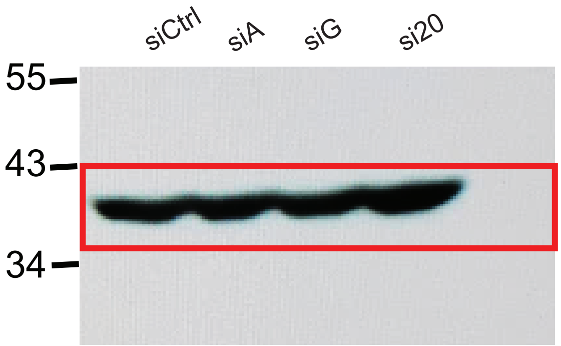

gg **Supp. Fig. 3a**

FANCD2

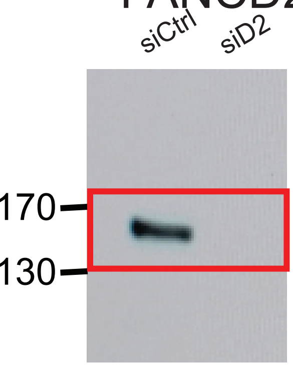

hh **Supp. Fig. 3a**

Actin (D2)

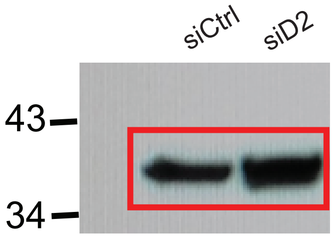

ii **Supp. Fig. 3a**

Actin (20)

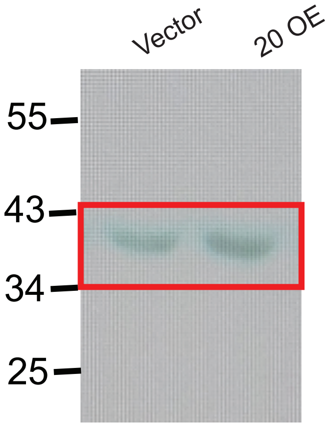

jj **Supp. Fig. 3a** BRCA2

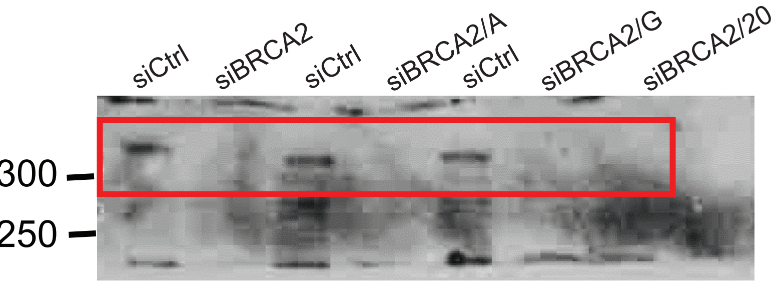

kk **Supp. Fig. 3a** Actin (A2)

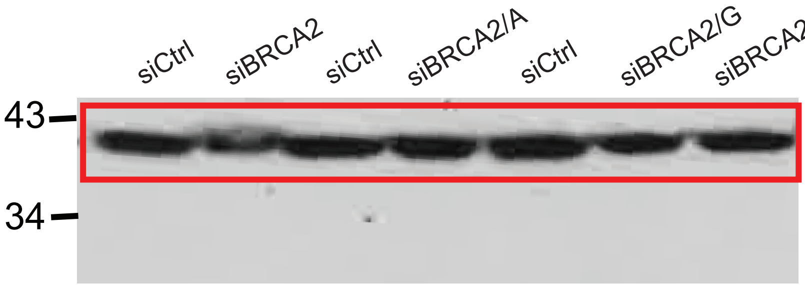

U2OS-283 FANCA KO

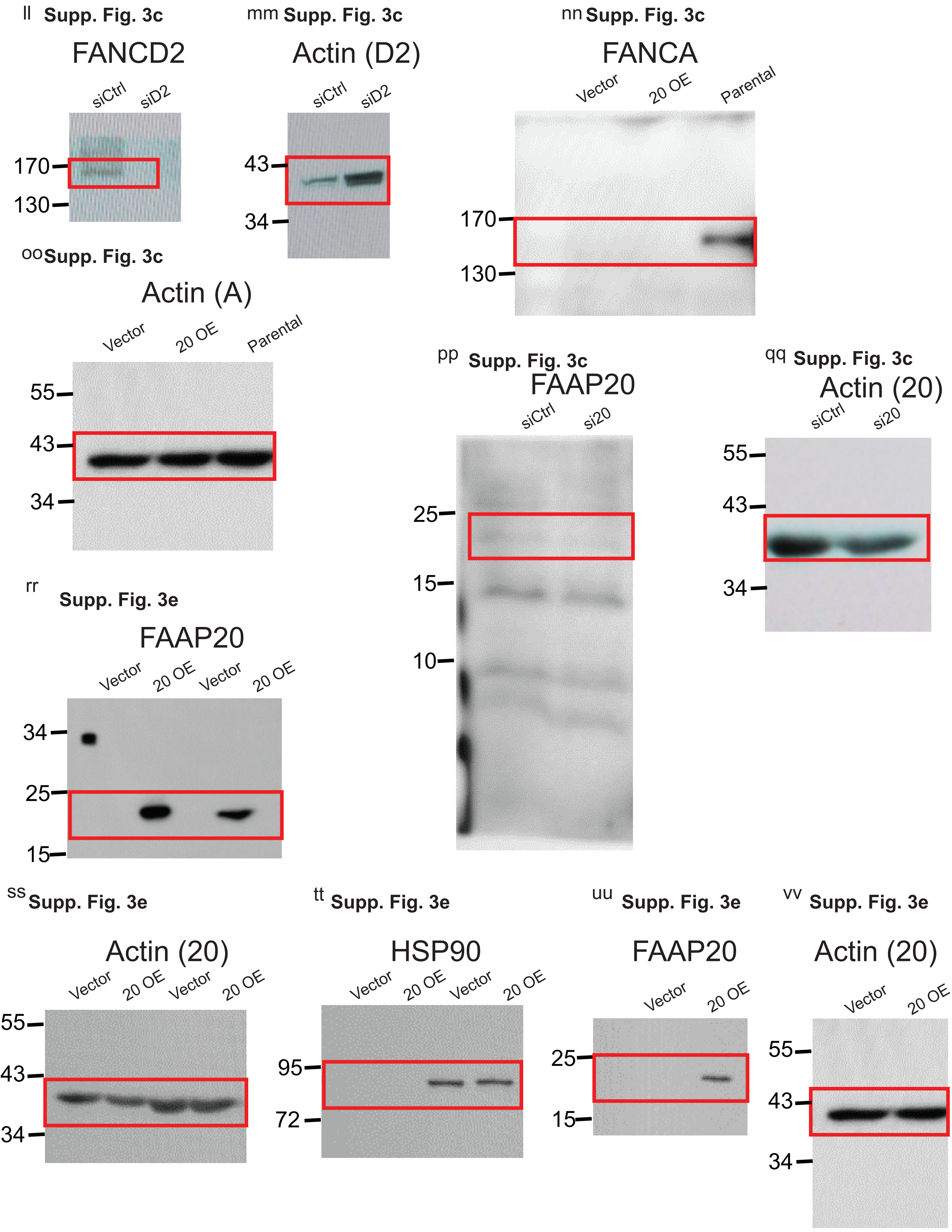

U2OS-283

ww **Supp. Fig. 3d**

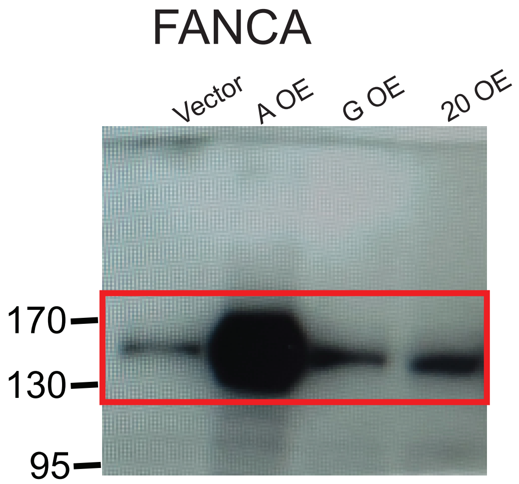

xx **Supp. Fig. 3d**

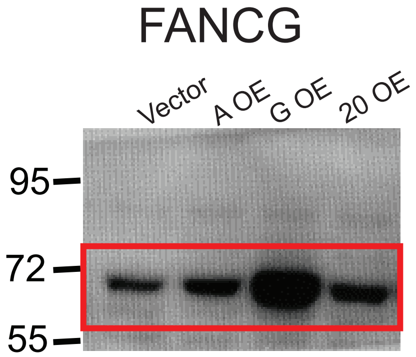

yy **Supp. Fig. 3d**

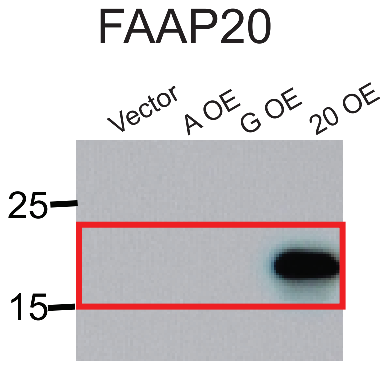

zz **Supp. Fig. 3d**

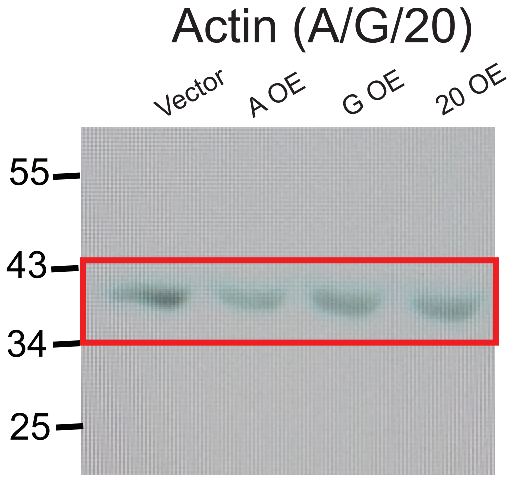

MIA-Paca2

aaa **Supp. Fig. 6a**

FANCA

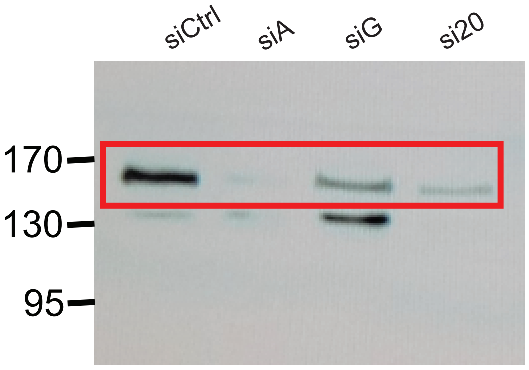

bbb **Supp. Fig. 6a**

FAAP20

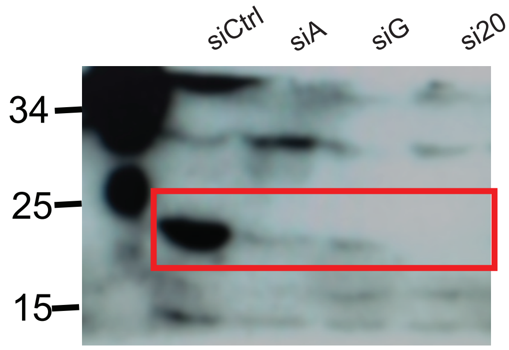

ccc **Supp. Fig. 6a**

Actin (A/20)

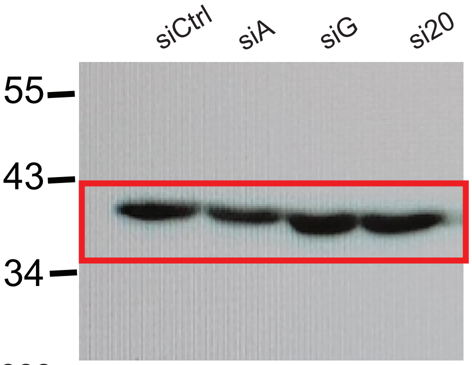

ddd **Supp. Fig. 6a**

FANCG

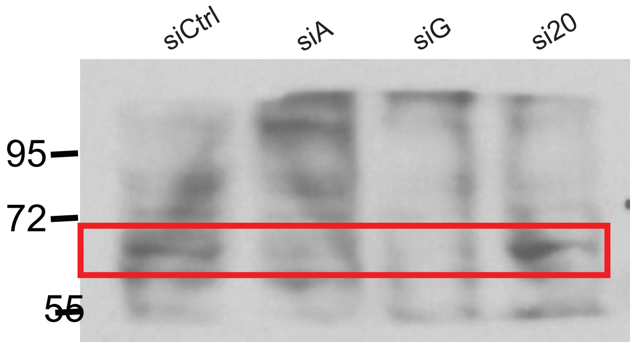

eee **Supp. Fig. 6a**

Actin (G)

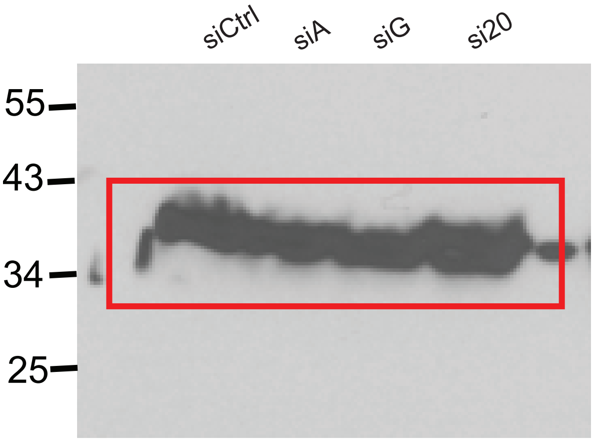

HeLa

fff **Supp. Fig. 6b**

FANCA

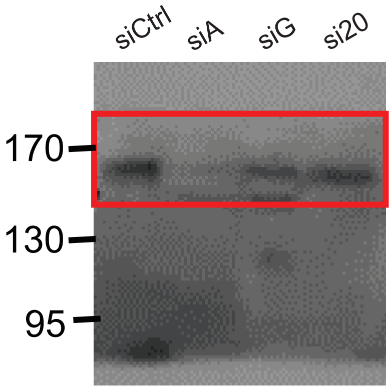

ggg **Supp. Fig. 6b**

Actin (A)

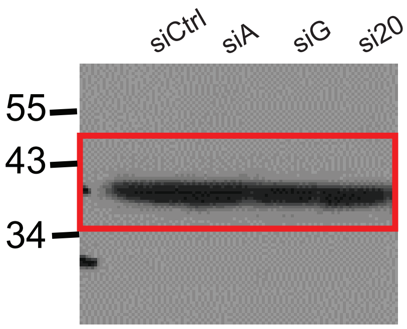

hhh **Supp. Fig. 6b**

FANCG

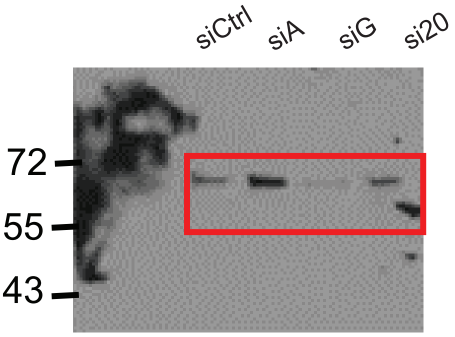

iii **Supp. Fig. 6b**

FAAP20

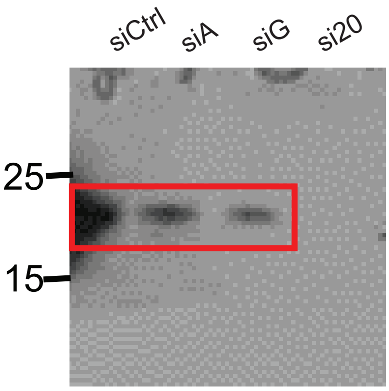

jjj **Supp. Fig. 6b**

Actin (G/20)

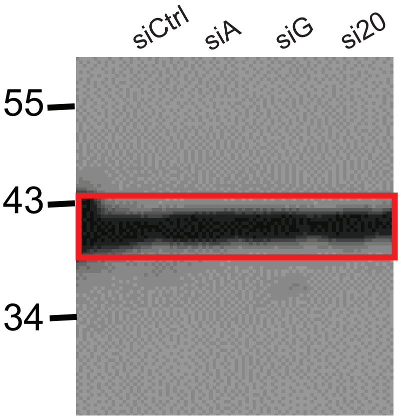

U2OS

kkk Supp. Fig. 6c

FANCG

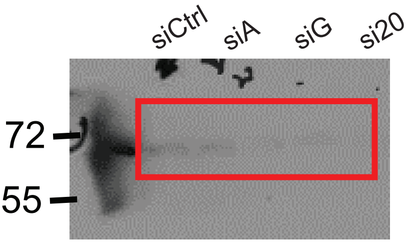

lll Supp. Fig. 6c

Actin (G)

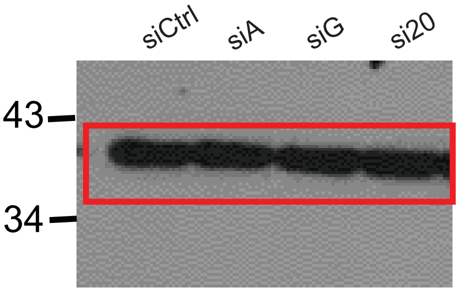

mmm Supp. Fig. 6c

FAAP20

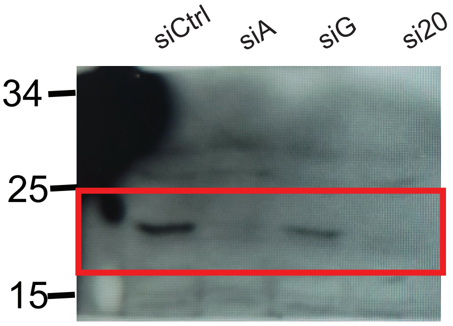

nnn Supp. Fig. 6c

Actin (20)

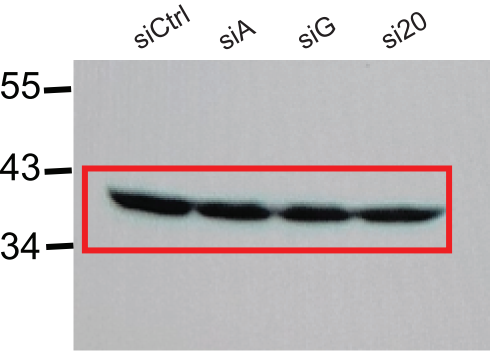

ooo Supp. Fig. 6c

FANCA

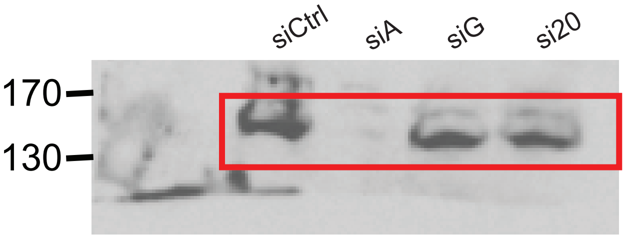

ppp Supp. Fig. 6c

Actin (A)

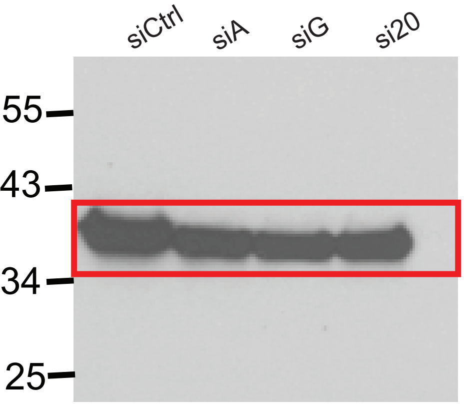

DiVA-U2OS

qqq Supp. Fig. 6d

FAAP20

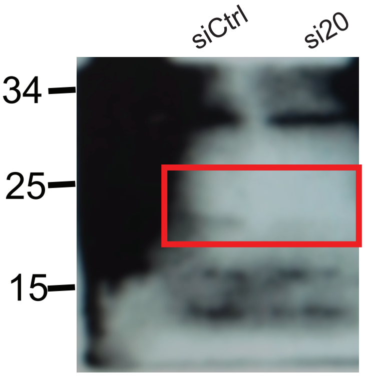

rrr Supp. Fig. 6d

Actin (20)

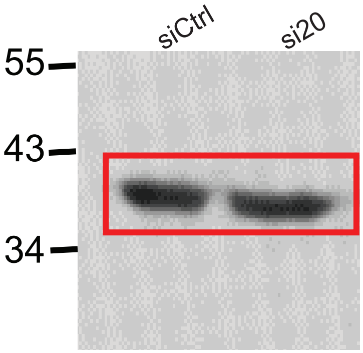

DiVA-U2OS + 4OHT

sss **Supp. Fig. 7b**

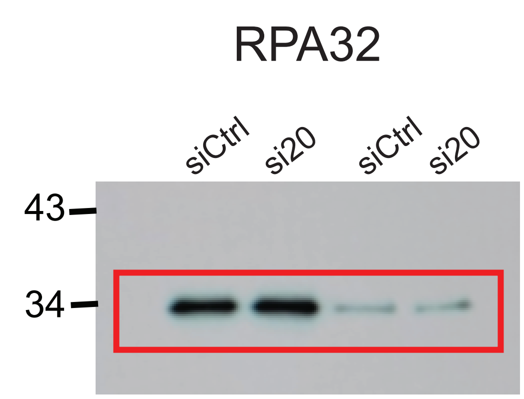

ttt **Supp. Fig. 7b**

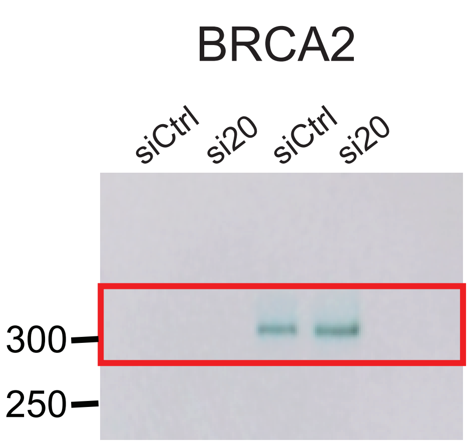

uuu **Supp. Fig. 7b**

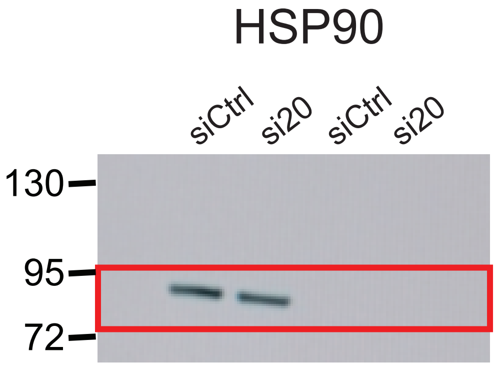

vvv **Supp. Fig. 7b**

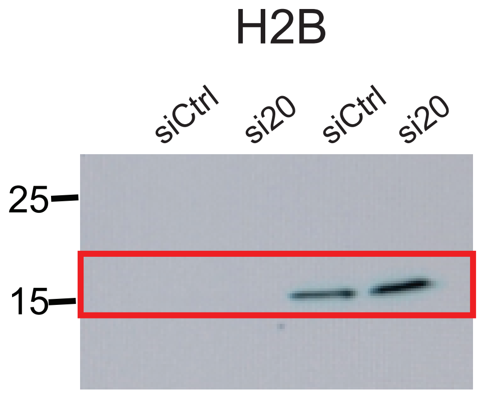

Supplementary Figure 8. Unprocessed Western blots.

Western blots shown correspond to figures 4g, 4h, Supp. Fig. 1b, Supp. Fig. 2a, Supp. Fig. 2c, Supp. Fig. 2d, Supp. Fig. 3a, Supp. Fig. 3c, Supp. Fig. 3e, Supp. Fig. 3d, Supp. Fig. 6a, Supp. Fig. 6b, Supp. Fig. 6c, Supp. Fig. 6d, Supp. Fig. 7b. Western membranes were trimmed around the desired M.W prior to development, and/or were developed using film which resulted in the loss of the M.W ladder on the membrane. However, prior to trimming/developing, the ladder location was marked on the membrane using pencil, allowing the size marker to be added back digitally after scanning.

Supplementary table 1. Oligonucleotide sequences

EMSA ssDNA 61-mer	GACGCTGCCGAATTCTACCAGTGCCTTGCTAGGACATCTTTGCCCACCTGCAGGTTCACCC
SSA substrate-1	TACGTTGTAAAACGACGGCCAGTGAATTCGAGCTCGGTACCCGGAGATCCTCTAGAGTCGACCTGCAGTGGCTT
SSA substrate-complement	ATGCAACATTTTGCTGCCGGTCACTTAAGCTCGAGCCATGGGCCTCTAGGAGATCTCAGCTGGACGTCACCGAA
Splayed arm substrate-1	**AGGTCTCGACTAACTCTAGTCGTTGTTCCA**CCCGTCCACCCGACGCCACCTCCTG
Splayed arm complement	**GCTGGCACGGTCGGATTTAAAGTTA**GGGCAGGTGGGCTGCGGTGGAGGACG
Strand exchange substrate	GCTGGCACGGTCGGATTTAAAGTTATCCAGAGCTGATTGAGATCAGCAACAAGGTGGGCAGGTGGGCTGCGGTGGAGGACG
EMSA ssRNA 61-mer	GACGCUGCCGAAUUCUACCAGUGCCUUGCUAGGACAUCUUUGCCCACCUGCAGGUUCACCC
Δ7-reporter CRISPR sgRNA sequence-5’	ACCACCCTGACCTACGGCTA
Δ7-reporter CRISPR sgRNA sequence-3’	GGGTAGCGGCTGAAGCAATG
Δ7-RMR ssDNA repair template	T*C*GGGGTAGCGGCTGAAGCACTGCACGCCGTAGGTCAGGGTGGTC*A*C
Δ7-RMR H1 repair template	T*C*GGGGTAGCGGCUGAAGCACTGCACGCCGUAGGUCAGGGTGGTC*A*C
Δ7-RMR H2 repair template	T*C*GGGGTAGCGGCTGAAGCACUGCACGCCGTAGGTCAGGGTGGTC*A*C
Δ7-RMR ssRNA template	U*C*GGGGUAGCGGCUGAAGCACUGCACGCCGUAGGUCAGGGUGGUC*A*C
ChIP qPCR primer-1	FW: CCGCCAGAAAGTTTCCTAGA/REV: CTCACCCTTGCAGCACTTG
ChIP qPCR primer-2	FW: CCGTCCGTTACGTAGAATGC/REV: GGGCGGGGATTATGTAATTT
ChIP qPCR primer-3	FW: GAGGAGCGCAGGACACTG/REV: CCAATTAGAGACCACCCGTTT
ChIP qPCR primer-4	FW: TCCCCTGTTTCTCAGCACTT/REV: CTTCTGCTGTTCTGCGTCCT
ChIP qPCR primer-5	FW: ATCGGGCCAATCTCAGAGG/REV: GCGACGCTAACGTTAAAGCA
ChIP qPCR primer-6	FW: GGAAGGAGGGGCTACTAGGG/REV:GAAAGCCCCATTCAGTTTGA

Bold: splayed arm portion

Underline: Ribonucleotides

*= Phosphorothioated bases
