# Supplementary material for: Fanconi anemia associated protein 20 (FAAP20) plays an essential role in homology-directed repair of DNA double-strand breaks
Source: Commun Biol. 2023 Aug 24;6:873. doi: 10.1038/s42003-023-05252-9 (PMC10449828; doi:10.1038/s42003-023-05252-9)
Supplement: Supplementary file 3 — Description of Additional Supplementary Files [file 42003_2023_5252_MOESM3_ESM.pdf]

Description of Additional Supplementary Files

File name: Supplementary Data
Description: Graph data point values are present in the Supplementary Data file
