# Supplementary material for: Fanconi anemia associated protein 20 (FAAP20) plays an essential role in homology-directed repair of DNA double-strand breaks
Source: Commun Biol. 2023 Aug 24;6:873. doi: 10.1038/s42003-023-05252-9 (PMC10449828; doi:10.1038/s42003-023-05252-9)
Supplement: Supplementary file 4 — Supplementary Data [file 42003_2023_5252_MOESM4_ESM.pdf]

Figure 1 source data

1a

siCtrl	100	100	100
siA	80	72.4	65.8
siG	93.3	86.2	51.2
si20	10	13.7	14.6
siBRCA2	4	4	2

1b

siCtrl	100	100	100
si20	7.5	9.6	0
siD2	18.9	17.3	20.3

1c

siCtrl WT	100	100	100
siCtrl + A KO	94	73.1	62.7
si20 + A KO	0	0	0
si20 WT	10	13.7	14.6
siD2 WT	18.9	17.3	20.3
siD2 + AKO	11.3	13.5	11.9

1e

siCtrl	100	100	100
siA	72.3	92.1	138
siG	72.3	71.2	134
si20	23.2	0	48.3

1f

siCtrl	100	100	100
siA	145	103	87.2
siG	117	114	82.3
si20	10.5	5.7	2.7

Figure 2 source data

2a

siCtrl	100	100	100
siA	11	11	33
siG	33	66	22
si20	22	44	11

2b

siCtrl	100	100	100
si20	17.6	18.1	5.2
siD2	11.8	4.5	11.1

2c

siCtrl WT	100	100	100
siCtrl + A KO	17.6	22.7	0
si20 + A KO	0	22.7	0
siD2 + AKO	5.9	0	0

2d

Vector	100	100	100
A-OE	78.8	100	100
G-OE	61	100	90.5
20-OE	163	150	143

2e

siCtrl	100	100	100
siBRCA2	910	983	713
siBRCA2/A	490	286	313
siBRCA2/G	440	817	517
siBRCA2/20	250	317	254

2f

Vector + DMSO	100	100	100
Vector + DI03	10.5	19.2	12.9
A-OE + DI03	14	15.3	9.7
G-OE + DI03	12.3	11.5	16.1
20-OE + DI03	22.8	23.1	12.9

2g	(-) OHT	(-) OHT	(+) OHT	(+) OHT
1	0.08047	0.07229	0.50199	0.54048
2	0.12383	0.56117	0.87097	0.75685
3	0.156897	0.097435	0.573461	1.026286
4	0.198411	0.118411	0.500729	0.667539
5	0.21339	0.006161	0.443177	0.409761
6	0.209752	0.32108	0.595568	0.767689

Figure 4 source data

4a

siCtrl	84.65473	85.42274	75.5287
si20	77.46479	84.53947	81.10092
siA	78.57143	81.43713	75.11013
siG	79.24528	73.95973	77.34139

4b

siCtrl	48.8665	55.71429	54.16667
si20	30.09321	48.74652	43.86874
siA	48.06867	40.23324	48.61751
siG	60.74766	46.66667	46.94656

4c

siCtrl	17.58621	12.93103	20.36364
si20	5.691057	0.44843	5.627706
siA	4.705882	2.259887	4.347826
siG	9.660574	6.407323	9.505208

4e

siCtrl	66.24685	55.71429	59.375
si20	52.72969	71.58774	60.44905
siA	58.79828	56.85131	63.36406
siG	68.01347	46.66667	46.94656

4f	G0/G1	G0/G1	G0/G1	S	S	S	G2/M
siCtrl	64.7	59.6	68.5	16.9	16	15.7	18.1
siA	63	66.3	65.5	22.9	16.2	16	13.5
siG	65.1	66	65.8	17.3	16.5	16.7	17.4
si20	66.3	66.8	66.9	12.4	11.7	12.6	21.1

Figure 4 source data (continued)

4f

G2/M	G2/M
24.3	15.4
17.2	18.2
17.2	17.2
21.5	20.4

Figure 5 source data

5a

siCtrl	100	100	100
siA	77	81.6	79.5
siG	87.1	92	99
si20	41	26.6	59

5b

siCtrl	100	100	100
siA	42.3	100	95.5
siG	15.4	65.2	71.9
si20	15.4	60.1	39.3

5c

siCtrl	100	100	100
siA	63.9175	59.5092	64.2857
siG	3.0928	14.4172	5.8442
si20	1.2887	7.6687	1.2987

Figure 6 source data

6a	Untreated			0.5 Gy		
siCtrl	100	100	100	80.8	62.4	52.9
si20	41	26.6	59	31	21.4	37.6
6b						
siCtrl	100	100	100	23.3	94.2	58.1
si20	15.4	60.1	39.3	2.1	1.4	2.8
6c						
siCtrl untreated	100	100	100			
si20 untreated	19.9	27.7	39.8			
siCtrl 4OHT	66.3	43.3	55.7			
si20 4OHT	4	14	13			
6d	DMSO			PARPi		
siCtrl	100	100	100	8.1	24.7	36.3
si20	46.2	37.6	18.9	1.9	3	10
6e						
siCtrl	100	100	100	64.5	39.2	54
si20	49.6	20.1	28.7	7.9	6	10.3

Supplementary Figure 1 source data

Supp. Fig. 1c	mCherry + GFP			GFP only		
siCtrl	3	2.9	4.1	1.5	1.3	1.9
siA	2.4	2.1	2.7	1.2	0.8	1.1
siG	2.8	2.5	2.1	1.2	0.8	0.6
si20	0.3	0.4	0.6	0.1	0.1	0.2
siA/G	1.6	1.3	2.7	0.8	0.4	1
siA/20	0.8	0.8	0.8	0.3	0.3	0.3
siA/G/20	1.1	0.5	1	0.5	0.2	0.4

Supp. Fig. 1d			
siCtrl	57.3	52.8	54
siA	58.7	45.5	48.9
siG	49.3	37.7	33.9
si20	43.5	41.7	46.7
siA/G	53.5	35.7	43.6
siA/20	54.2	46	47
siA/G/20	56	39.9	48.4

Supp. Fig. 1e	G0/G1			S			G2/M
siCtrl	58.2	55.1	61.6	13.3	16.8	16.8	27.2
siA	60.1	67.7	63	15.4	14	10.9	24
siG	57.8	57.6	62.5	12.5	17	15.4	28.6
si20	71.4	74	74.4	6.44	7.65	7.58	21.4
siFANCD2	65.6	65.8	63.2	9.99	13.5	15.3	23.3

Supplementary Figure 1 source data (Continued)

Supp. Fig. 1e

25.8	19.6
16.3	25.1
23.6	20.7
17.5	17.2
18.2	19.7

Supplementary Figure 2 source data

Supp. Fig. 2b

siCtrl KO	81.1	66	76.3	9.22	29.1	20.1	8.76
si20	88.8	69.9	72.5	4.39	29.5	20.5	3.21
siD2	88.1	69.1	78.2	7.61	20.6	16.2	4.91
si20/D2	87	56.9	91.3	4.55	39	7.21	8.42
siCtrl WT	66.7	67.8	79.7	19.4	19.1	11.9	13.9

Supp. Fig. 2d

Vector	100	100	100
20-OE	83.2	44.5	93.4

Supp. Fig. 2e

DNA template	0.386887	0.406081	0.394302
RNA template	0	0	0
H1 template	0	0.014475	0.005269
H2 template	0.039662	0.03911	0.03875
CRISPR only	0	0.005292	0.00075

Supplementary Figure 2 source data (continued)

Supp. Fig. 2b

6.67	4.95
6.19	13.4
10.5	5.17
4.58	2.52
13.4	8.15

Supplementary Figure 3 source data

Supp. Fig. 3b	G1/G0		S			G2/M	
siCtrl	66.7	67.8	79.7	19.4	19.1	11.9	13.9
siA	82.9	75.1	80.2	9.95	14	11.8	6.64
siG	78.8	82.1	72.7	15.7	9.52	14.4	4.65
si20	85.9	93.9	81.8	9.42	2	11.7	4.21
siD2	65.9	72.5	82.1	16.5	17.3	9.5	16.9

Supplementary Figure 3 source data (continued)

13.4	8.15
10.3	7.43
7.51	14.4
4.37	7.07
8.26	9.23

Supplementary Figure 4 source data

Supp. Fig. 4b

	(-) OHT	(+) OHT
1	0.076252	0.52193
2	0.26278	0.810525
3	0.123873	0.766803
4	0.153566	0.577114
5	0.036321	0.426888
6	0.256482	0.676859

Supp. Fig. 4c

1	0.268288	0.492636
2	0.351563	0.271419
3	0.774779	0.494893
4	0.653016	0.726242
5	0.377668	0.559363
6	1.068206	0.527968

Supp. Fig. 4d

1	0.121744	0.18199
2	0.125167	0.095078
3	0.124878	0.247459
4	0.217426	0.230887
5	0.184957	0.209535
6	0.4133	0.298388

Supp. Fig. 4e

1	21.78103	54.75808
2	7.674113	9.000468
3	1.443929	81.57188
4	72.25372	11.51147
5	5.35171	232.3249
6	62.90052	23.18307

Supp. Fig. 4f

1	19.20398	216.7668
2	13.43881	40.83309
3	22.23869	9.724787
4	8.456144	92.1982
5	5.022248	5.95871
6	11.43195	28.97359

Supp. Fig. 4g

Supplementary Figure 4 source data (continued)

1	2.38943	6.483023
2	19.02731	7.004749
3	2.921414	14.706
4	4.521078	7.276947
5	4.800995	7.276947
6	10.13776	21.8062

Supplementary Figure 7 source data

Supp. Fig. 7a	DMSO			DI03		
siCtrl	100	100	100	45.2	8.7	10
si20	49.6	20.1	28.7	9.7	4	4.3
