# Supplementary material for: Fanconi anemia associated protein 20 (FAAP20) plays an essential role in homology-directed repair of DNA double-strand breaks
Source: Commun Biol. 2023 Aug 24;6:873. doi: 10.1038/s42003-023-05252-9 (PMC10449828; doi:10.1038/s42003-023-05252-9)
Supplement: Supplementary file 1 — Peer Review File [file 42003_2023_5252_MOESM1_ESM.pdf]

nature portfolio

Peer Review File

Fanconi anemia associated protein 20 (FAAP20) plays an essential role in homology-directed repair of DNA double•strand breaks

Open Access This file is licensed under a Creative Commons Attribution 4.0 International License, which permits use, sharing, adaptation, distribution and reproduction in any medium or format, as long as you give appropriate credit to the original author(s) and the source, provide a link to the Creative Commons license, and indicate if changes were made. In the cases where the authors are anonymous, such as is the case for the reports of anonymous peer reviewers, author attribution should be to 'Anonymous Referee' followed by a clear attribution to the source work. The images or other third party material in this file are included in the article's Creative Commons license, unless indicated otherwise in a credit line to the material. If material is not included in the article's Creative Commons license and your intended use is not permitted by statutory regulation or exceeds the permitted use, you will need to obtain permission directly from the copyright holder. To view a copy of this license, visit http://creativecommons.org/licenses/by/4.0/.

Reviewers' comments:

Reviewer #1 (Remarks to the Author):

In this manuscript, the authors report the roles of FAAP20, a direct binding partner of FANCA and a component of the Fanconi anemia (FA) core complex necessary for the repair of interstrand crosslinks (ICLs), in modulating distinct types of homology-directed repair of DNA double-strand breaks (DSBs). Mostly using siRNA-mediated knockdown of FAAP20, FANCA, FANCG, or in combination, they show that FAAP20 has a FA core complex-independent function in DNA/RNA template-mediated DSB repair, works with FANCA to support the single-strand annealing (SSA) repair pathway, and promotes RAD51 loading in response to irradiation to augment HR repair, which is associated with radiosensitivity and PARP inhibitor sensitivity.

Overall, this is an interesting story that proposes a new role of FAAP20 outside the canonical FA DNA ICL repair, which presumably works independently of the FA core complex that functions as a ubiquitin E3 ligase complex to trigger FANCD2 monoubiquitination. The in vitro biochemical assays to demonstrate the stimulatory role of FAAP20 in FANCA-dependent strand exchange and annealing assays are novel and convincing, providing important insight into how the FAAP20-FANCA heterodimer may participate in SSA. However, a significant number of other cellular assays that involve siRNA knockdown are confusing, of poor quality, and performed without proper controls. Essentially, the multiple proposed roles of FAAP20, both claimed FANCA-independent and -dependent, are not well supported by the cellular phenotypes of a broad HR defect in FAAP20-depleted cells. The FAAP20 phenotypes are pleiotropic, which are often inconsistent, depending on the experimental conditions that are used, especially only one FAAP20 siRNA is employed throughout the study. Formatting could be much improved for better flowing of the story, and there are many cases where graphs are not presented in a correct manner. More specific points are presented below.

1. One of the major claims of this manuscript is that there is a FANCA-independent role of FAAP20 in template-mediated HR repair. The FANCA and FAAP20 constitute a heterodimer within the FA core complex, in which their stability is inter-dependent, so this new idea needs to be well substantiated. This also indicates that there should be a pool of FAAP20 that exists independently of FANCA while retaining its stability. To corroborate this idea, there should be a clear Western blot showing that siRNA FANCA leads to complete FANCA depletion, in which condition there is still residual FAAP20 in the same sample, but this information is missing in Figure 1. Blots in Figures 1 and S1 are of poor quality and fail to show the interrelation of FAAP20, FANCA, and FANCG stability one another when each gene is knocked down. Instead of displaying multiple low-quality immunoblots that only confirm target gene depletion, the authors should show an integrated image that visualizes the impact of each knockdown to other counterpart (e.g. FANCA KD -> FAAP20 level along with FANCA itself). How much FAAP20 is left after complete FANCA depletion is important to support the FAAP20-specific function, if any.

Moreover, the FANCA-independent FAAP20 phenotypes should be confirmed by at least two independent siRNA FAAP20. Its effect to cell cycle needs to be tested to exclude the non-specific effect of FAAP20 depletion and cell viability issues. Nonspecific toxicity of a single siRNA appears to be a big concern especially this siRNA causes reduced survival (e.g., in clonogenic survival; Fig. 6 compared to other FANC genes, FANCA and FANCG); therefore, it is not due to impairment of the FA pathway, but more likely caused by a nonspecific effect. Additionally, no information is provided on how long cells were transfected with siRNA, which can negatively affect the viability.

2. A bigger conceptual problem is that the idea regarding the various roles of FAAP20, i.e., FANCA-independent template-mediated repair, FANCA-dependent SSA, IR-inducible RAD51 foci formation, and NHEJ, is disjointed and does not help interpret the cellular phenotypes exhibited upon FAAP20 depletion. Especially, Figure 1 (i.e., template repair) may well be caused by an artifact of insufficient knockdown, nonspecific toxicity, or cell cycle problems, etc. There is no evidence to support its unique

role in this repair process at this moment. Even the FANCA-independent role of FAAP20 is true, there is no explanation on how the stability could be maintained or how it is recruited to damage sites. Is it polyubiquitination mediated as previously reported? Again, all the works are done using an artificial reporter assay with a single knockdown, and the physiological implication of this observation is completely lacking.

In this sense, the authors may consider removing Figure 1 and focusing on the role of FAAP20 in assisting the function of FANCA in SSA repair, which is much more convincing as it is supported by clear biochemical data. Refocusing the story into the role of FAAP20 in promoting SSA via FANCA regulation may be a better way of presenting data as an immediate follow-up of the authors' previous report (Benitez et al., 2018).

There are many cases of confusing and inconsistent data;

3. Figure S1c: why does mCherry+GFP induce higher %GFP positivity compared to GFP only? Also, why does siRNA A/20 together induce higher %GFP than siRNA FAAP20, which is not consistent with the proposed model. Again, insufficient knockdown or nonspecific effects of multiple siRNA transfection could be an issue.

4. Figure 2a: why is control 0.9 while all other graphs are fixed at 1.0? Also, why does siRNA A/G/20 rescue the phenotype of siRNA A/20? Unless there is a specific negative role of FANCG in this process, a non-specific effect of multiple siRNA transfection can be the reason for this.

5. Figure 2b: both FAAP20 and FANCG are proposed to promote the FANCA-dependent SSA repair. Then why does A/G-OE (overexpression) fail to increase the SSA event unlike the 20-OE? This result makes reliability of this assay questionable.

6. Figure 2c: This mutant study suggests that FAAP20 promotes SSA exclusively via FANCA. The level of FANCA needs to be checked to determine whether the positive role of FAAP20 is to increase FANCA levels. However, if so, it is contradictory to Figure 2b, where FANCA overexpression itself is not sufficient to increase SSA. Furthermore, it does not make sense as A/G-OE fails to increase SSA events in Figure 2b while FAAP20 alone can increase them.

7. Figures 5d and 5e should appear before Figure 5a and 5b when flow of the manuscript is considered.

8. Figure 6a and 6b: co-depletion of FANCA and FAAP20 rescues the cytotoxic effect of FAAP20 knockdown, suggesting that FANCA and FAAP20 are antagonistic in this condition, and that FANCA is deregulated in the absence of FAAP20. The same trend is noted in overexpression studies. However, this is in contrast to the overall claim of the story, and there is no explanation for this negative regulation.

9. Figure S4a and S4b: The NHEJ phenotypes are very mild and do not add to the main point of how FAAP20 promotes HR. If FAAP20 also promotes NHEJ, it does not make sense how HR can be positively regulated since HR and NHEJ are generally antagonistic one another.

10. Figure 6d; description in the main text in page 11 does not make sense. There is no rescue.

11. Some of the speculations to explain the phenotype in Result section should be moved to Discussion (e.g., page 8). On the other hand, Discussion is too heavily focused on reiterating the experimental results.

12. Y-axis labeling is incorrect in lots of graph that display DNA repair efficiency. (%) control cannot be between 0 to 1. It should be "repair efficiency (relative to control)" with a scale of 0-100; This

mistake has been noted in numerous cases, e.g., Figure 1a, 1b, 1f, 2a, 2b, 2c, 4b. Similarly, (%) control should be changed to "fold change", e.g., Figure 4a. These need to be corrected throughout the manuscript.

13. The manuscript is too dense with too much details. Extensive editing is recommended.

Reviewer #2 (Remarks to the Author):

Zhang etc. reported the previously unknown functions of FAAP20, involved in HR, SSTR, and SSA. These are important DNA-damage repair mechanisms, which can be activated by radiation or certain chemicals. Defects in HR, SSTR and/or SSA can all lead to cancer development and progression. HR, SSTR and/or SSA can be considered as the potential targets for cancer therapy, as inhibiting the repair proteins or the pathways could lead to the accumulation of DNA damage and cells death in cancer.

As authors realized, FAAP20 is known for being involved in the Fanconi anemia pathway, a DNA repair pathway that is activated in response especially to DNA interstrand cross-links (ICL). FAAP20 is one of the components of the FA core complex, which is responsible for the recognition and repair of DNA ICL. To some extent, HR, SSTR and SSA are also involved in ICL repair for completely resolving ICL DNA damage. However, Zhang etc. through sophisticated, in vitro biochemical assays, as well as demonstrating actual HR, SSTR or SSA events inside cells, concluded that FAAP20 could directly participate in these repair mechanisms, independent of the canonical FA signaling pathway. Therefore, the reported work is important not only for enhancing our understanding of in-depth DNA damage repair mechanisms but also possessing significant translation impact on chemotherapy resistance or sensitivity.

Here, this reviewer would suggest conducting another supplementary experiment to further strengthen the unique roles that FAAP20 plays, which is FAAP20-ChIP (as done for FANCA ChIP shown in Extended data Fig 4), through using cells treated with IR, compared to the cells treated with ICL agents (UV), in time-dependent manner. Briefly, collecting cell samples at 2, 4, 6h post IR treatment; similarly for collecting ICL-carrying cells. The dynamics of FAAP20 binding to the DNA substrate under two conditions would further support the novel points of the work. However, this is optional as authors have provided the sufficient amount of information to support the novel findings.

In addition, there is a typo for missing Figure number in noting the Extended Data at the end of Result Section

A minor revision for the MS.

Reviewer #3 (Remarks to the Author):

Remarks to the Author：
This manuscript by Professor Zhang and colleagues reports data suggesting that FAAP20 has multiple important roles in several HDR repair pathways. It reports that besides widespread belief, FAAP20 has a marked role in HR after DSB that is not associated with an ICL and is not epistatic with its FA binding partner FANCA. This is a nice compelling study, quite thoroughly done using many complementary techniques from cell-based and in vitro assays to in vivo work and cytogenetics. The findings are novel or at least not widely appreciated.
However, there are some data quality issues that need to be addressed. In some cases, the data do not support individual conclusions.

My comments：

1. The results that prove the function of FAAP20/FANCA/FANCG were based on one siRNA oligo, which is not convincing. It's better to use at least two siRNA oligos or siRNA pool.

2. The mechanism of SSA reduction caused by overexpression has not been further studied, and the relevant content was not presented in the final model. Was it caused by the increase of downstream protein recruitment? It is better to carry out further experimental verification.

3. Although the paper believed that FAAP20 could participate in the effect of HDR without the effect of FANCA, it was only proved by the FACS results of U2OS. On the contrary, in vitro biochemical experiments and drug sensitivity experiments have proved that FANCA and FANCG may play a certain role in the HDR.

4. Although the possible link between FaAP20 and FANCA has been confirmed, the upstream and downstream proteins of FAAP20 in the pathway are not clear. Some experiments on the interaction between FAAP20 and FANCA during HDR can be added. Or further study of the function between BRCA1 and FAAP20. Or do some experiments to study the specific effects of FAAP20 on RAD51 and the relationship between FAAP20 and BRCA2. Also, do FANCI and FANCD2 play a role in this?

5. Some Western blot results were too ugly, especially the bands of FANCG in fig1. were unclear, and the effect of siRNA cannot be determined. Therefore, the experimental results are not credible, and western should be optimized.
In overexpression experiment, the protein expression level of different samples should be adjusted as consistent as possible, which can reduce the influence of protein levels on the experimental results.

6. In the cell colony experiment, the results of U2OS and Hela were not completely consistent. The authors can try other cell lines, such as HCT116 or normal cell line RPE1, for further verification.

7. Scar bar was not labeled in immunofluorescence figure of Fig5

UNIVERSITY OF MIAMI
MILLER SCHOOL
of MEDICINE

Yanbin Zhang, PhD
Professor of Biochemistry & Molecular Biology
Department of Biochemistry & Molecular Biology
University of Miami Miller School of Medicine
1011 NW 15th Street
Gautier Building Room 311
Miami, FL 33136

June 2, 2023

Dear reviewers,

We are pleased to resubmit our manuscript entitled: “Fanconi anemia associated protein 20 (FAAP20) plays an essential role in homology-directed repair of DNA double-strand breaks.” We would like to thank the reviewers for their helpful suggestions and commentary. In this revised manuscript, we have carefully addressed all concerns. We believe that this version of our manuscript is greatly improved. Major changes to the main text are highlighted in yellow. We would like to highlight some of the major concerns that have been addressed:

1. The use of only one siRNA molecule: In our study we have always used an siRNA pool of (4) different siRNA molecules for each targeted protein. From the concerns listed by multiple reviewers, we can see that this was not clearly stated in our initial submission. As seen below, we have added this statement in several places within the manuscript and have reiterated the use of siRNA pools in all of our experiments within this letter.
2. The quality of western blots: We have redone most western blots to obtain better quality figures. In addition, we have added an explanation about the challenges in obtaining FA protein western blots in Reviewer #3’s concern #5.
3. Inconsistencies in cellular assays: We have added colony formation/cell proliferation assays in additional cell lines (MIA PaCa-2) and have reported consistent findings for FAAP20 KD conditions. Cell assays showing KD of multiple proteins simultaneously (A/20, A/G, A/G/20) have been removed due to consistency issues.

Specific points (major and minor) have all been addressed and are listed below. Responses are highlighted by italic font.

Reviewer #1
Concern #1: One of the major claims of this manuscript is that there is a FANCA-independent role of FAAP20 in template-mediated HR repair. The FANCA and FAAP20 constitute a heterodimer within the FA core complex, in which their stability is inter-dependent, so this new idea needs to be well substantiated. This also indicates that there should be a pool of FAAP20 that exists independently of FANCA while retaining its stability. To corroborate this idea, there should be a clear Western blot showing that siRNA FANCA leads to complete FANCA depletion, in which condition there is still residual FAAP20 in the same sample, but this information is missing in Figure 1. Blots in Figures 1 and S1 are of poor quality and fail to show the interrelation of FAAP20, FANCA, and FANCG stability one another when each gene is knocked down. Instead of displaying multiple low-quality immunoblots that only confirm target gene depletion, the authors should show an integrated image that visualizes the impact of each

knockdown to other counterpart (e.g. FANCA KD -> FAAP20 level along with FANCA itself). How much FAAP20 is left after complete FANCA depletion is important to support the FAAP20-specific function, if any.

__Response:__ *We agree that this claim needs to be well-substantiated. We have added the suggested western blots to* ***Extended Data Figure 1b, Extended Data Figure 2a and c****,* ***Extended Data Figure 3a****,* ***and Extended Data Figure 6a, b, and c****. We have redone most of these blots to obtain better quality images. From these western blots, we do observe a population of FAAP20 in FANCA KD cells, even though it is reduced compared to the control. We believe that this pool of FAAP20 is sufficient to carry out DNA repair activities. Also, the true amount of FAAP20 present in these cells may be obscured by technical limitations, i.e lack of commercially available FAAP20 antibody with high binding affinity and specificity. Additionally, we have performed western blots for FAAP20 in 282-U2OS cells with FANCA KO using CRISPR/Cas9 and included densitometry analysis to show that FAAP20 protein is present in FANCA KO cells and is subsequently depleted with siFAAP20 siRNA KD. This western blot with densitometry analysis is located in Extended Data Figure 2a.*
Moreover, the FANCA-independent FAAP20 phenotypes should be confirmed by at least two independent siRNA FAAP20.

__Response:__ *We agree this is an excellent point that needs full clarification, and we understand that could have seriously impacted the validity of the work. Therefore, we want to clarify that for every protein knockdown in every experiment, we always used an siRNA pool consisting of (4) distinct targeting siRNAs for each gene. We do see that this was not clearly stated in the manuscript, and we apologize for not doing so. We have added this information to the second paragraph of the Results section and have also stated this in the Methods section under "Cell culture."*

Its effect to needs to be tested to exclude the non-specific effect of FAAP20 depletion and cell viability issues. Nonspecific toxicity of a single siRNA appears to be a big concern especially this siRNA causes reduced survival (e.g., in clonogenic survival; Fig. 6 compared to other FANC genes, FANCA and FANCG); therefore, it is not due to impairment of the FA pathway, but more likely caused by a nonspecific effect. Additionally, no information is provided on how long cells were transfected with siRNA, which can negatively affect the viability.

__Response:__ *We appreciate the concern, and we agree that toxicity of a single siRNA is possible. We addressed this concern by using a pool of siRNA for every experiment. Therefore, we believe the loss of colony formation is due to a specific function of FAAP20 that is separable from its FANCA function and likely has to do with its role in HR and not the FA pathway. These assays support our claim that FAAP20's HR role is not redundant with FANCA, as observed by the difference in colony formation. Loss of colony formation is a common readout for HR dysfunction, as HR proteins have prominent roles in protecting the replication fork from stress. Therefore, defects in HR can present as a loss of ability to proliferate, as seen in the colony formation experiment. Because pools were used for all proteins and all KD conditions were performed in the same way, the lack of effect for FANCA and FANCG can add validity to the specific role of FAAP20. The siRNA transfection conditions including how long the cells were transfected were added to the Methods section under "Cell culture."*

__Concern #2:__ A bigger conceptual problem is that the idea regarding the various roles of FAAP20, i.e., FANCA-independent template-mediated repair, FANCA-dependent SSA, IR-

inducible RAD51 foci formation, and NHEJ, is disjointed and does not help interpret the cellular phenotypes exhibited upon FAAP20 depletion.

<u>Response:</u> *We agree that the role of FAAP20 is confusing, so the NHEJ figure was removed because this is hard to explain and does not show a major effect. However, we believe that measurement of RAD51 irradiation-induced foci is a standard readout for HR activity based on the literature:*
1. Wang, Z., et al., The Homologous Recombination Repair Pathway is Associated with Resistance to Radiotherapy in Nasopharyngeal Carcinoma. Int J Biol Sci, 2020. 16(3): p. 408-419.

2. Powell, S.N. and L.A. Kachnic, Therapeutic exploitation of tumor cell defects in homologous recombination. Anticancer Agents Med Chem, 2008. 8(4): p. 448-60.

3. O'Dea, R. and C. Santocanale, Non-canonical regulation of homologous recombination DNA repair by the USP9X deubiquitylase. J Cell Sci, 2020. 133(3).

We also feel that FAAP20's roles in SSTR and SSA fit within the umbrella of HDR and provide clues into the placement of FAAP20 in this pathway.

Especially, Figure 1 (i.e., template repair) may well be caused by an artifact of insufficient knockdown, nonspecific toxicity, or cell cycle problems, etc. There is no evidence to support its unique role in this repair process at this moment. Even the FANCA-independent role of FAAP20 is true, there is no explanation on how the stability could be maintained or how it is recruited to damage sites.

<u>Response:</u> *We really appreciate the great level of scrutiny. We agree that artifacts are always a reason for concern. We have vetted this concern in several different ways. First we only analyze live cells by flow cytometry that way any nonspecific toxicity would be gated out from the analyzed cell population. Then we analyzed cell cycle distribution in (2) of our GFP-reporter cell lines and saw that only FAAP20 KD (Not FANCA or FANCG) causes G1 accumulation, it has been documented (Kostyrko, K., et al., A role for homologous recombination proteins in cell cycle regulation. Cell Cycle, 2015.* ***14****(17): p. 2853-61.) that HR deficits can result in G1 accumulation, therefore this provides further support for a specific effect of FAAP20 in HR. These data have been added in* ***Extended Data Figure 2b*** *and* ***Extended Data Figure 3b****.*

*The question as to whether FAAP20 is stable in the absence of FANCA is an important question.To throughly test the stability of FAAP20 in the absence of FANCA we utilized cell lines where we performed FANCA knock out using CRISPR-CAS9 to fully eliminate the contribution of residual FANCA protein. We find that FAAP20 is clear and present in FANCA KO cells at steady state showing that the loss of FANCA does not completely ablate the presence of FAAP20. When we performed the siFAAP20 KD in this FANCA KO cell line there is well over a 100 fold difference between the FAAP20 protein levels between the siCtrl and the siFAAP20. This is consistent with the residual FAAP20 band in the western blots from other work reporting on the stability of FAAP20 in the absence of FANCA (*Ali, A.M., et al., *FAAP20: a novel ubiquitin-binding FA nuclear core-complex protein required for functional integrity of the FA-BRCA DNA repair pathway.* Blood, 2012. **119**(14): p. 3285-94.*). We would like to note that this work (Ali et. al) did not compare these FAAP20 levels between a FANCA KD and a FAAP20 KD nor did it evaluate any functional capacity of the remaning FAAP20 after FANCA loss, as this was not the purpose of their paper. Here, we are showing not only is there a substantial*

amount of FAAP20 after loss of FANCA but the remaning amount is enough to facitite the HDR function as seen by the loss of HR in the FAAP20 KD but not the FANCA KO. We also find that FAAP20 can be stably overexpressed and found in nuclear protein extracts in FANCA KO cells, as shown in ***Extended Data figure 3e****. This is because FAAP20 is recruited to damage in an RNF8-dependent and ubiquitin-dependent manner, not in a FANCA-dependent manner. FAAP20 is important for FANCA damage recruitment, however, indicating that FAAP20 acts upstream of FANCA (Yan, Z., et al., A ubiquitin-binding protein, FAAP20, links RNF8-mediated ubiquitination to the Fanconi anemia DNA repair network. Mol Cell, 2012.* ***47****(1): p. 61-75).*

Is it polyubiquitination mediated as previously reported?

Response: *It is likely ubiquitin mediated as the C-term UBZ domain is required for FAAP20 chromatin recruitment. To test this, a mutation would need to be made in the C-term UBZ domain that ablates ubiquitin binding, and this mutant would need to be stably expressed in a FAAP20 deficient cell line to see if this mutant can rescue HR-deficient phenotypes caused by the loss of FAAP20. Due to the excessive time and effort that this would require, we cannot complete these experiments, but we feel that this would be an interesting direction to explore in a following study.*

Again, all the works are done using an artificial reporter assay with a single knockdown, and the physiological implication of this observation is completely lacking.

Response: *We thank the reviewer for their insight, and we strongly agree that there are substantial limitations that currently exist to interrogate the roles of various proteins in the different DNA repair pathways. While there are some more antiquated methods such as those seen in (Donoho G, Jasin M, Berg P. Analysis of gene targeting and intrachromosomal homologous recombination stimulated by genomic double-strand breaks in mouse embryonic stem cells. Mol Cell Biol. 1998 Jul;18(7):4070-8. doi: 10.1128/MCB.18.7.4070. PMID: 9632791; PMCID: PMC108991.) that rely on southern blot and lack single cell resolution of FACS, we are using what is currently considered the standard approach to investigate the role of a protein in the different DNA repair pathways as seen in numerous other well cited works (Howard, S.M., D.A. Yanez, and J.M. Stark, DNA damage response factors from diverse pathways, including DNA crosslink repair, mediate alternative end joining. PLoS Genet, 2015. 11(1): p. e1004943.; Gunn, A. and J.M. Stark, I-SceI-based assays to examine distinct repair outcomes of mammalian chromosomal double strand breaks. Methods Mol Biol, 2012. 920: p. 379-91.; Stark, J.M., et al., Genetic steps of mammalian homologous repair with distinct mutagenic consequences. Mol Cell Biol, 2004. 24(21): p. 9305-16.). We do recognize that even these experimental techniques characterizing HR proteins are extremely limited. Therefore, we conducted extensive supporting experiments that look at PARPi sensitivity, radiosensitivity, and DI03 sensitivity that are established physiological implications of HR deficiency. They also serve as clinical applications because they show how cancer cells use FAAP20 to overcome these strategies that target the DNA damage response.*

In this sense, the authors may consider removing Figure 1 and focusing on the role of FAAP20 in assisting the function of FANCA in SSA repair, which is much more convincing as it is

supported by clear biochemical data. Refocusing the story into the role of FAAP20 in promoting SSA via FANCA regulation may be a better way of presenting data as an immediate follow-up of the authors' previous report (Benitez et al., 2018).

Response: *This is an excellent recommendation and a path that we would have pursued if we were not able to establish the stability of FAAP20 in the absence of FANCA and show that a substantial amount of FAAP20-dependent HR continues to be present and valid in FANCA KO cells. We fully agree any such finding alone is not convincing. We continued in this work to show that role of FAAP20 in HR occurs after the recruitment of RPA, after the recruitment of BRCA2, and before the recruitment of RAD51. We also go on to provide supporting evidence in the form of colony formation assays. If FAAP20 is indeed required for a substantial percentage of HR, then there should be a correspondingly large impact on the ability for the cells to proliferate when only knocking down FAAP20, and we have demonstrated that across multiple cell lines. We also provide support that it is HR that FAAP20 is participating in through using PARPi as well as DI03 where is it well established that HR defects cause additional loss of cell proliferation in the presence of PARPi and DI03. Given that FAAP20 is showing strong prognostic value in cancer in the clinical setting we believe that it is important that our well-supported findings are made available for consideration as the community continues to investigate the role of FAAP20 in DNA repair. While we have removed a sizeable number of other findings from the work, we believe the finding of FAAP20 in HR is important and would like to be able to share this data publicly; we also believe it is related to its role in SSA, albeit not an identical function, but should be published together. As HDR deficits are becoming more are more relevant we believe more work is needed that looks at the comprehensive role of proteins under the HDR umbrella.*

<u>Concern #3</u>: Figure S1c: why does mCherry+GFP induce higher %GFP positivity compared to GFP only?

Response: *This is an excellent point the reviewer notices here and we would like to take this opportunity to point out the importance of this difference. The cell populations representing "GFP only" includes cells that were not transfected but are not able to be excluded because there is not way to differentiate them. The % is higher in mCherry+GFP because we are first gating only on transfected cells by looking at the mcherry + (transfected cells) and not the total cell population. We are then looking at the GFP+ populations only within the transfected cells therefore eliminating cells that were GFP- because they were not transfected. This improvement we made to the assay not only helps accurately determine the percentage of GFP+ cells that are present, but also helps eliminate changes to transfection efficiency that could result from any of our experimental conditions, thereby reducing the potential presence of artifacts.*

Also, why does siRNA A/20 together induce higher %GFP than siRNA FAAP20, which is not consistent with the proposed model. Again, insufficient knockdown or nonspecific effects of multiple siRNA transfection could be an issue.

Response: *We thank the reviewer for this insightful question. We believed that the loss of A and 20 may have resulted in some changes in pathway choice that was functioning as a compensatory mechanism. We found that without further investigation and potentially epistasis*

analysis those findings are not currently necessary for this study. This data has been removed from the manuscript.

<u>Concern #4</u>: Figure 2a: why is control 0.9 while all other graphs are fixed at 1.0?

<u>Response:</u> *We thank the reviewer for bringing this to our attention. This was an error that has been corrected.*

Also, why does siRNA A/G/20 rescue the phenotype of siRNA A/20? Unless there is a specific negative role of FANCG in this process, a non-specific effect of multiple siRNA transfection can be the reason for this.

<u>Response:</u> *Thank you for carefully considering this. While we have consistently observed this result in our experiments, we agree that it is hard to explain. Due to our inability to identify a mechanism for this occurrence, we found these data are not needed for the current work and we have removed this data from the manuscript.*

<u>Concern # 5:</u> Figure 2b: both FAAP20 and FANCG are proposed to promote the FANCA-dependent SSA repair. Then why does A/G-OE (overexpression) fail to increase the SSA event unlike the 20-OE? This result makes reliability of this assay questionable.

<u>Response:</u> *We appreciate the reviewer bringing attention to this matter as we find it to be an important point. While FANCG and FAAP20 both promote FANCA-dependent SSA repair it does not necessitate that they are both limiting factors. Many biochemical processes that require multiple proteins rarely occur such that all proteins are equally rate-limiting or necessary. FANCG and FAAP20 share a biochemical function: the ability to stimulate substrate binding and processing activities of FANCA. However, because they are different proteins with different domains, we also believe it is possible that they have different cellular contexts in which they are stimulating FANCA and could therefore have separable roles. This hypothesis can be supported by the following findings by others and ourselves:*

-The AG20 subcomplex was not able to stimulate FANCI/FANCD2 monoubiquitination in vitro, in contrast with the other subcomplexes of the FA core complex (van Twest, S., et al., Mechanism of Ubiquitination and Deubiquitination in the Fanconi Anemia Pathway. Mol Cell, 2017. ***65****(2): p. 247-259.)*

-The molecular structure of A/G has been solved both alone and in the context of the entire FA core, however the structure of FANCA/FAAP20 has not been solved yet. This means there is also no evidence that FAAP20 binds FANCA simultaneously with FANCG or that FAAP20 has the same effect as FANCG when bound to FANCA.

-From our own studies in this manuscript: our in vitro biochemistry reactions show that the addition of both FANCG and FAAP20 to FANCA at the same time does not cause a synergistic increase in activity. This means that binding of FANCG to FANCA is dispensable for stimulating FANCA activity when FAAP20 is already present. If only one protein is needed for FANCA stimulation at a time, this raises the question of why both proteins are able to perform the same biochemical activity with the same binding partner? Our explanation is that these roles are not meant to be redundant, but separable with FANCG-stimulation taking place in a different context than FAAP20-stimulation.

*-With BRCA2 loss and SSA upregulation (**Figure 2e**), simultaneous FAAP20 knockdown decreases SSA significantly while FANCG knockdown does not, further strengthening that FAAP20 may have a more prominent role in SSA than FANCG and is one example of a cellular condition where FAAP20-stimulation of FANCA is preferred over FANCG.*

<u>Concern # 6a:</u> Figure 2c: This mutant study suggests that FAAP20 promotes SSA exclusively via FANCA. The level of FANCA needs to be checked to determine whether the positive role of FAAP20 is to increase FANCA levels. However, if so, it is contradictory to Figure 2b, where FANCA overexpression itself is not sufficient to increase SSA. Furthermore, it does not make sense as A/G-OE fails to increase SSA events in Figure 2b while FAAP20 alone can increase them.

<u>Response:</u> *This is very constructive. We evaluated the impacted levels of FANCA and find that FAAP20 overexpression increases FANCA protein level, and this is now shown in **Extended Data Figure 3d**. We also find FANCA overexpression does not detectably increase FAAP20 levels. This indicates that overexpression of FAAP20 vs FANCA are not equivalent.*

<u>Concern # 6b:</u> However, if so, it is contradictory to Figure 2b, where FANCA overexpression itself is not sufficient to increase SSA. Furthermore, it does not make sense as A/G-OE fails to increase SSA events in Figure 2b while FAAP20 alone can increase them.

<u>Response:</u> *This is an interesting point that we are excited to address. Similar to what we mentioned in the beginning part of the response to concern #5 it is unlikely that FANCA, FANCG, and FAAP20 are all limiting factors in the context of SSA. Given that the main focus of this work is to demonstrate that FAAP20 is a critical player in HDR we believe that promotion of FANCA-dependent SSA resulting from the overexpression of FAAP20 but not FANCA or FANCG strongly supports this claim. The point that FAAP20 but not A, G, or A/G – OE is sufficient to increase SSA is very consistent with our updated data (due to the reviewer's suggestion) showing that FAAP20-OE increases FANCA levels but FANCA-OE does not increase FAAP20 levels*

Concern # 7: Figures 5d and 5e should appear before Figure 5a and 5b when flow of the manuscript is considered.

<u>Response:</u> *We agree, and this change has been made. Thank you.*

<u>Concern # 8:</u> Figure 6a and 6b: co-depletion of FANCA and FAAP20 rescues the cytotoxic effect of FAAP20 knockdown, suggesting that FANCA and FAAP20 are antagonistic in this condition, and that FANCA is deregulated in the absence of FAAP20. The same trend is noted in overexpression studies. However, this is in contrast to the overall claim of the story, and there is no explanation for this negative regulation.

<u>Response:</u> *This is a great point. We realize that this result requires more explanation. After careful consideration, similar to the other studies regarding double and triple knockdown, we have decided that this work does not need those studies, so we have removed it from the manuscript.*

Concern # 9: Figure S4a and S4b: The NHEJ phenotypes are very mild and do not add to the main point of how FAAP20 promotes HR. If FAAP20 also promotes NHEJ, it does not make sense how HR can be positively regulated since HR and NHEJ are generally antagonistic one another.

Response: *We agree that this result is difficult to interpret and does not support the main findings of the manuscript, so we have removed it.*

Concern # 10: Figure 6d; description in the main text in page 11 does not make sense. There is no rescue.

Response: *We thank the reviewer for requesting this clarification as it could have been confusing to the reader. In accordance with the other data sets of this nature we found that it is not required for support of the findings in this work, and this has been removed.*

Concern #11: Some of the speculations to explain the phenotype in Result section should be moved to Discussion (e.g., page 8). On the other hand, Discussion is too heavily focused on reiterating the experimental results.

Response: *We greatly appreciate the reviewer suggestions here as we also feel that the suggestions greatly improve the readability. We substantially revamped the Results and discussion section. We have trimmed back the Results section and moved the speculation to the Discussion. We have also removed much of the Discussion section pertaining to reiteration of the experimental results.*

Concern # 12: Y-axis labeling is incorrect in lots of graph that display DNA repair efficiency. (%) control cannot be between 0 to 1. It should be "repair efficiency (relative to control)" with a scale of 0-100; This mistake has been noted in numerous cases, e.g., Figure 1a, 1b, 1f, 2a, 2b, 2c, 4b. Similarly, (%) control should be changed to "fold change", e.g., Figure 4a. These need to be corrected throughout the manuscript.

Response: *We have corrected these errors for all graphs.*

Concern # 13: The manuscript is too dense with too much details. Extensive editing is recommended.

Response: *We appreciate the constructive comments from this reviewer, and we understand this concern and have extensively edited and removed extraneous details from both the Results and Discussion sections.*

Reviewer #2

Concern #1: Here, this reviewer would suggest conducting another supplementary experiment to further strengthen the unique roles that FAAP20 plays, which is FAAP20-ChIP (as done for FANCA ChIP shown in Extended data Fig 4), through using cells treated with IR, compared to the cells treated with ICL agents (UV), in time-dependent manner. Briefly, collecting cell

samples at 2, 4, 6h post IR treatment; similarly for collecting ICL-carrying cells. The dynamics of FAAP20 binding to the DNA substrate under two conditions would further support the novel points of the work. However, this is optional as authors have provided the sufficient amount of information to support the novel findings.

Response: *We thank this reviewer for the excellent suggestion. While we were not able to perform the suggested experiment, in Figure 6c we perform cell proliferation assays after FAAP20 KD in DiVA-U2OS with or without treatment with 4-OHT. Since 4-OHT induces DSB damage specifically through induction of the AsiSI nuclease, this allows us to see the effect of FAAP20 KD on cell growth in cells experiencing DSBs only and not ICLs. As seen in* ***Figure 6c****, we do see that loss of FAAP20 sensitizes cells to DSB damage which supports our hypothesis about FAAP20's prominent role in DSB repair that is independent of ICLs.*

Concern #2: In addition, there is a typo for missing Figure number in noting the Extended Data at the end of Result Section

Response: *We thank the reviewer for noticing this. This has been removed.*

Reviewer #3

Concern #1: The results that prove the function of FAAP20/FANCA/FANCG were based on one siRNA oligo, which is not convincing. It's better to use at least two siRNA oligos or siRNA pool.

Response: *We apologize for the lack of clarity and we strongly agree this is a very important detail to clarify and highlight. For all experiments a siRNA pool with (4) siRNAS was always used for each KD in the study. We have added this to the Results section paragraph 2 and to the methods section under "Cell Culture."*

Concern #2: The mechanism of SSA reduction caused by overexpression has not been further studied, and the relevant content was not presented in the final model. Was it caused by the increase of downstream protein recruitment? It is better to carry out further experimental verification.

Response: *We thank the reviewer for these useful insights and suggestions. We agree that this finding needed more delineation. While we were not able to delineate the entire mechanism behind this occurrence, we do believe that FAAP20's ability to increase SSA involves FAAP20's role as a limiting factor in this pathway, and also involves FAAP20's relationship with FANCA. As seen from our KD experiments, both FAAP20 and FANCA strongly support SSA with a shared function (****Fig. 2a and c****). To further evaluate the SSA increase resulting from FAAP20 overexpression we evaluated the protein levels of FANCA following FAAP20 overexpression. When we overexpressed FAAP20 in 283 cells, we see that this increases FANCA protein levels, but we cannot detect similar increases in FAAP20 when FANCA is overexpressed (****Extended Data Fig. 3d and e****). This can explain why FAAP20 overexpression, but not FANCA overexpression is sufficient to increase SSA even though both factors have important roles in this pathway. This is also supported by data (Which we have since removed), showing that overexpression of a FAAP20 mutant variant that is deficient in FANCA-interaction is not able to upregulate SSA. In fact, overexpression of a FANCA binding-deficient FAAP20 variant causes a significant decrease in SSA which may mean this mutant protein acts in a dominant negative*

fashion during SSA. This further shows that FAAP20 increases SSA at least partially through its interaction with FANCA. This relationship is also unidirectional as FANCA overexpression itself is not able to sufficiently increase FAAP20 protein levels.

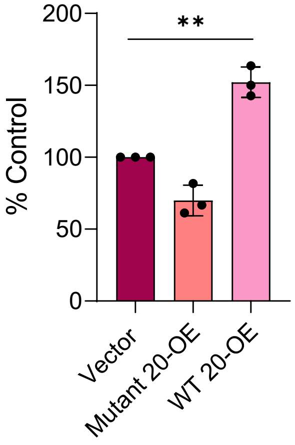

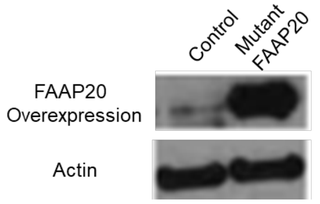

Concern #3: Although the paper believed that FAAP20 could participate in the effect of HDR without the effect of FANCA, it was only proved by the FACS results of U2OS. On the contrary, in vitro biochemical experiments and drug sensitivity experiments have proved that FANCA and FANCG may play a certain role in the HDR.

Response: *From our experiments, FANCA depletion does cause a decrease in HR, but FAAP20 causes a bigger decrease. Our previous in vitro studies have shown that FANCG stimulates FANCA's biochemical strand annealing/exchange activity but how/where this activity is utilized in HR has not been defined so far. Also, from our current experiments in this manuscript, we do see that both FANCA and FANCG affect RAD51 IRIF, which does also point to a role for these proteins in certain cases of HR. We would not like to make claims against these potential roles of FANCA/FANCG, we would only like to claim, based on our data, that the role of FAAP20 does not exclude FANCA/FANCG but may have additional significance. This is based on FACS analysis in 2 different HR assays (**Figure 1**), G1 accumulation of cells with FAAP20 KD but not FANCA/FANCG KD (**Extended Data Fig. 2b and 3b**), and consistent significant loss of cell proliferation across 3 different cancer cell lines seen with FAAP20 KD only (Figure 5).*

Concern #4: Although the possible link between FAAP20 and FANCA has been confirmed, the upstream and downstream proteins of FAAP20 in the pathway are not clear. Some experiments on the interaction between FAAP20 and FANCA during HDR can be added. Or further study of the function between BRCA1 and FAAP20.

Response: *We agree this is important to the study. We performed IF staining for BRCA1 after FAAP20 KD and after 2 Gy IR treatment and did not see a significant change in BRCA1 IRIF (**Figure 4b**).*

Or do some experiments to study the specific effects of FAAP20 on RAD51 and the relationship between FAAP20 and BRCA2.

Response: *This is a great suggestion that drastically improves the study. In addition to IF staining showing that FAAP20 depletion reduces RAD51 IRIF, we also performed coIP using anti-RAD51 antibody in HEK293t extracts after FAAP20 overexpression, and we see that RAD51 specifically pulls down FAAP20 protein, showing that these proteins form an interaction (**Figure 4h**). We also performed nuclear fractionation and western blotting in DiVA-U2OS cells after FAAP20 KD and DSB induction by 4-OHT treatment. We did not see any detectable differences in BRCA2 nuclear recruitment in siCtrl vs siFAAP20-treated DiVA-U2OS cells (Extended Data Fig. 7b). We also did not see consistent, detectable changes in nuclear RPA with FAAP20 KD (**Extended Data Fig. 7b**). Together, we believe these results place the role of FAAP20 directly before RAD51 damage loading and may facilitate RAD51 damage loading itself. But FAAP20 does not seem to act upstream in HR pathway decisions by influencing BRCA1 or BRCA2 damage recruitment.*

Also, do FANCI and FANCD2 play a role in this?

Response: *We appreciate this insightful inquiry and suggestions. We have performed the cell-based GFP recombination assays in 282 and 283-U2OS WT and FANCA KO backgrounds with FANCD2 KD and comparison with FAAP20 KD (**Figure 1b and c; Figure 2b and c**). We see that FANCD2 KD causes a greater decrease in HR than FANCA, so its role does not appear redundant with FANCA. However, FAAP20 KD causes an even more significant decrease in HR compared with FANCD2. We believe this is because FAAP20 and FANCD2 are acting together in most cases of HR, but FAAP20 seems to have a slightly more prominent role. Yet both proteins seem more prominent in HR than FANCA. In the case of SSA (283 cells), however, FANCD2 KD does not cause a significant difference in SSA than FAAP20 KD, which is also not significantly different than FANCG KD or FANCA KD/KO. This further suggests that the FA pathway, or at least multiple FA proteins, has a common function in carrying out the SSA pathway.*

Concern #5: Some Western blot results were too ugly, especially the bands of FANCG in fig1. were unclear, and the effect of siRNA cannot be determined. Therefore, the experimental results are not credible, and western should be optimized.
In overexpression experiment, the protein expression level of different samples should be adjusted as consistent as possible, which can reduce the influence of protein levels on the experimental results.

Response: *We greatly appreciate this reviewer's in depth concern and heavily addressed them with a great deal of importance. We have redone many of the westerns showing FANCA/FANCG/FAAP20 KD **(Extended Data Fig. 1, 2, and 3**). In our FA field, it is known that obtaining good-quality western blots for FA proteins is quite challenging due to the lack of good quality antibodies and low protein abundance in cells. In some fortuitous cases, there are very good antibodies for frequently studied proteins such as FANCD2 and FANCA. However, for FANCG and FAAP20 proteins, there are very few commercially available antibodies and the available antibodies have poor affinity and specificity. Some examples in other articles with similar FA protein western blots include:*

1. *Velimezi, G., et al., Map of synthetic rescue interactions for the Fanconi anemia DNA repair pathway identifies USP48. Nat Commun, 2018.* ***9****(1): p. 2280. (****Figure 3a and 6a-FANCC****)*
2. *Huang, Y., et al., Modularized functions of the Fanconi anemia core complex. Cell Rep, 2014.* ***7****(6): p. 1849-57. (****Figure 2a-FANCG, and Figure 3a-FANCL; Figure 4d-FANCL****)*
3. *Bick, G., et al., Coordination of the recruitment of the FANCD2 and PALB2 Fanconi anemia proteins by an ubiquitin signaling network. Chromosoma, 2017.* ***126****(3): p. 417-430. (****Figure 6a and d-FAAP20****)*

In the case of FAAP20, most studies do not study endogenous FAAP20 due to the difficulty in detecting this protein, so exogenous tagged FAAP20 (FLAG, His, HA, etc.) is usually utilized instead:

1. *Nagareddy, B., A. Khan, and H. Kim, Acetylation modulates the Fanconi anemia pathway by protecting FAAP20 from ubiquitin-mediated proteasomal degradation. J Biol Chem, 2020.* ***295****(40): p. 13887-13901.*
2. *Wang, J., et al., Prolyl isomerization of FAAP20 catalyzed by PIN1 regulates the Fanconi anemia pathway. PLoS Genet, 2019.* ***15****(2): p. e1007983.*
3. *Wojtaszek, J.L., et al., Ubiquitin recognition by FAAP20 expands the complex interface beyond the canonical UBZ domain. Nucleic Acids Res, 2014.* ***42****(22): p. 13997-4005.*

Due to the difficult nature of studying these endogenous proteins, we kindly ask our western blot data to be assessed based on the publication standard within the FA field as a whole.

<u>Concern #6</u>: In the cell colony experiment, the results of U2OS and Hela were not completely consistent. The authors can try other cell lines, such as HCT116 or normal cell line RPE1, for further verification.

<u>Response:</u> *We thank the reviewer for this excellent suggestion. MIA PaCa-2 cancer cell lines were added for further validation, because according to the Human Protein Atlas, FAAP20 protein is highly expressed in these cells which may indicate that these cells have a certain dependency on FAAP20 protein. We have also removed as much inconsistent colony formation data as possible.*

<u>Concern #7:</u> Scar bar was not labeled in immunofluorescence figure of Fig5

<u>Response:</u> *Scale bars have been added to this figure. Thank you.*

Again, thank you for your input and re-consideration of this manuscript.

Sincerely yours,

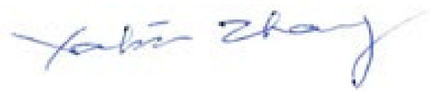

Yanbin Zhang

REVIEWERS' COMMENTS:

Reviewer #1 (Remarks to the Author):

The authors addressed most of the key concerns and significantly revised the manuscript. They removed a considerable amount of data that were not of high quality or not consistent, and thus now the manuscript is much clear and concise.

Reviewer #2 (Remarks to the Author):

Authors have addressed the critical concerns. This reviewer gives no more concerns or suggestions to further revise the current version of the manuscript.

Reviewer #3 (Remarks to the Author):

In the revised manuscript, the author answers some questions raised earlier by further experiments. Particularly, it shows that the independent function of FAAP20 in HDR is downstream of the 53BP1 and BRCA1 DSB repair choice process. And it may be closely related to the process of RAD51 loading into DNA, which is very important.
However, there are still some problems
1. Wrong marked in fig2. f
2. Wrong legend in fig2. G
3. The siFAAP20 knockdown efficiency showed in Extended fig2 wasn’t good.
4. Based on the hypothetical model showed in fig7, FAAP20 was participate in three downstream processes of HDR. According to the experimental results in the paper, it is convincing that FAAP20 and FANCA participate in SSA together, but there may be some doubts about the other two processes. Maybe, it would be better to further study the relationship between FAAP20 and RAD51

Dear Dr. Chong and Reviewers,

We are pleased to submit a final revision of our manuscript entitled: “Fanconi anemia associated protein 20 (FAAP20) plays an essential role in homology-directed repair of DNA double-strand breaks.” We would like to give our deepest thanks once again to the reviewers for their suggestions that have greatly improved the manuscript. We take the quality and readability of our work very seriously and would like to respond to the remaining concerns raised by Reviewer #3:

Reviewer #1:

No remaining concerns

Reviewer #2:

No remaining concerns

Reviewer #3:

Concern #1: Wrong marked in fig2. f

Response: *We thank the reviewer for their careful observation. We have reviewed this figure panel and ensured its accuracy.*

Concern #2: Wrong legend in fig2. G

Response: *We once again thank this reviewer for their observation. A new figure legend description has been added for Fig. 2g*

Concern #3: The siFAAP20 knockdown efficiency showed in Extended fig2 wasn’t good.

Response: *We are grateful for this high level of scrutiny in our western blots showing siRNA knockdown of FAAP20. We understand and agree that this figure could be a bit difficult to discern. Due to low protein abundance of FAAP20 and poor antibody availability, we often obtain grainy/high background images due to long exposure times needed for detection. To improve visualization of knockdown efficiency, we have sharpened the bands and increased brightness equally across the entire image. This allows the band in the siCtrl lane to appear more clearly, while it is still apparent that there is no detectable band in the si20 lane. We believe that it is now much easier to compare the first lane with the fourth lane and observe a clear difference in the presence of a FAAP20 protein band in the control conditions, but not the knockdown.*

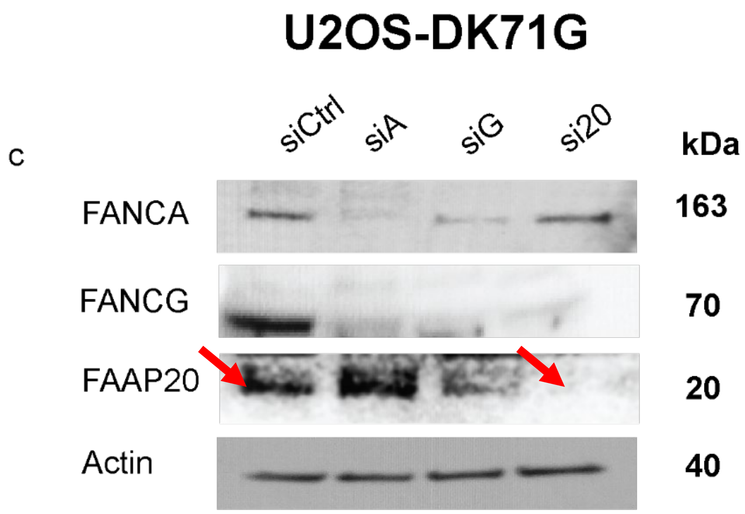

Concern #4: Based on the hypothetical model showed in fig7, FAAP20 was participate in three downstream processes of HDR. According to the experimental results in the paper, it is convincing that FAAP20 and FANCA participate in SSA together, but there may be some doubts about the other two processes. Maybe, it would be better to further study the relationship between FAAP20 and RAD51

Response: *This is a great observation. While we were able to show that FAAP20 knockdown causes a significant loss of canonical HR, SSTR, and SSA, only the SSA pathway appeared to be redundant with FANCA loss. Based on this result, we show FANCA association with FAAP20 only in the depiction of the SSA pathway in our model. We believe that this result is important in itself, even though further work is needed to elucidate more specific mechanisms. We are also very curious about the relationship between FAAP20 and RAD51 but given the space and time constraints associated with publishing our work, we plan to explore this mechanism further in a future study. In this manuscript, our goal was to establish a general role of FAAP20 in HDR, and then determine more specific mechanistic details in subsequent manuscripts. We hope the reviewer can understand our decision to organize these research topics into separate publications.*

Thank you for your consideration.
